# Global prevalence and ethnic variation of pathogenic BRCA1/2 variants in breast cancer: a systematic review and meta-analysis

**DOI:** 10.1186/s12967-026-07997-3

**Published:** 2026-03-12

**Authors:** Najeeb Ullah Khan, Huijun Lei, Jinzhen Fu, Ruijiao Lei, Xukai Chen, Sana S. Alqarni, Tianhui Chen

**Affiliations:** 1 Institute of Biotechnology and Genetic Engineering, The University of Agriculture Peshawar, Peshawar, 25130 Pakistan; 2 Department of Cancer Prevention, Zhejiang Cancer Hospital, Hangzhou, 310022 China; 3 Hangzhou Institute of Medicine (HIM), Chinese Academy of Sciences, Hangzhou, 310018 China; 4 Postgraduate Training Base Alliance of Wenzhou Medical University, Zhejiang Cancer Hospital), Wenzhou, 325000 China; 5 Department of Clinical Laboratory Science, College of Applied Medical Science, King Saud University, Riyadh, 11421 Saudi Arabia

**Keywords:** *BRCA1/2* mutation, Single nucleotide polymorphisms, Ethnicity, Breast cancer, Pathogenic, Global population

## Abstract

**Background:**

The breast cancer (BC) susceptibility genes 1 (*BRCA1*) and BC susceptibility genes 2 (*BRCA2*) are critical genes associated with hereditary breast cancer, and their mutation prevalence might greatly vary across different ethnic populations. This systematic review and meta-analysis evaluated global ethnic variation in *BRCA1/2* mutation prevalence among breast cancer (BC) patients.

**Methods:**

We searched five databases for studies published between 2015 and 2025 that reported *BRCA1/2* mutations in BC patients across various ethnic groups. 45 studies met the inclusion criteria, comprising about 44,000 BC patients. Data were stratified into two categories: (1) the frequency of all reported variants (including high-frequency polymorphisms) to assess global reporting patterns, and (2) the estimated clinical prevalence of confirmed Pathogenic and Likely Pathogenic (PLP) variants (excluding benign polymorphisms) for cancer risk assessment in broad ethnic categories (Asian, Chinese, Black/African descent, Hispanic/Latino, Middle Eastern/North African, European, Ashkenazi Jewish, and others).

**Results:**

The prevalence of *BRCA1/2* mutations in BC patients displayed substantial global variability. Heterogeneity was high (I² >95%, *p* < 0.001), reflecting diverse study populations and designs. The frequency of all reported variants varied substantially, reaching up to 17% in specific subgroups due to the inclusion of common polymorphisms. However, after strict filtering, the clinical prevalence of PLP variants ranged from < 1% to 5% in most ethnic groups, aligning with expected population risk.

**Conclusion:**

Ethnicity significantly influences *BRCA1/2* mutation distribution among BC patients globally. These findings underscore the importance of population-tailored genetic testing approaches and the necessity of including underrepresented groups in genetic research to enhance risk assessment and personalized cancer care.

**Supplementary Information:**

The online version contains supplementary material available at 10.1186/s12967-026-07997-3.

## Introduction

The breast cancer (BC) susceptibility genes 1 (*BRCA1*) and BC susceptibility genes 2 (*BRCA2*) are highly penetrant tumor suppressor genes whose germline mutations pose significantly augmented risks of BC in women around the globe [1]. *BRCA1*, located on chromosome 17q21, and *BRCA2*, on 13q12.3, are capable of encoding proteins of 1863 and 3418 amino acids in length, respectively. These proteins play a critical role in cellular damage response (DDR) and function compliantly to facilitate the repair of DNA double-strand breaks (DSBs) as a main component of the high-fidelity homologous recombination repair (HRR) pathway [2]. Loss-of-function mutation in both genes can result in HR deficiency (HRD), which means that BC cells could only rely on error-prone repair pathways, leading to the accumulation of DSBs, heightened genomic instability, and, possibly, BC development. Clinically BRCA-associated tumors show unique susceptibility to DNA-damaging therapies such as platinum drugs and Poly (ADP-ribose) Polymerase (PARP) inhibitors and potential resistance towards cyclin-dependent kinase 4 and 6 (CDK4/6) inhibitors [3, 4]. Hence, timely identification of BRCA1/2 status is an essential guide in clinical practice for strategizing effective therapeutic as well as prophylactic approaches for BRCA mutation carriers [5].

Pathogenic variants, including single-nucleotide polymorphisms (SNPs) in *BRCA1* and *BRCA2*, are found in approximately 5–10% of all mammary cancer cases [1, 6]. Healthy females who carry a deleterious *BRCA1* or *BRCA2* mutation pose a lifetime estimated 80% risk of developing BC by the age of ~ 70–80 years [7]. Women carrying these pathogenic *BRCA1/2* mutations develop BC at a younger age and are associated with a more aggressive BC type, such as that of triple-negative breast cancer (TNBC). The frequency of BRCA mutations in BC patients can be diverse depending on the patient’s ethnic composition and inclusion criteria. For instance, BRCA mutations are found in approximately 1–3% of patients with supposedly sporadic BCs, whereas the prevalence rises to ~ 10–20% in patients with early onset or familial breast cancer [8]. Similarly, the rate of pathogenic BRCA variant mutation was found to be different based on their self-identified ethnicity, for example, ~ 6.6% in Asian Indian patients, 9.2% in Black African patients, 18.1% in Europeans, 13.2% in patients of mixed ancestry ethnicity, all living in the same country, South Africa [9]. This evidence suggests that although BRCA mutations contribute substantially to the breast cancer risk globally, their prevalence is not uniform across global populations. Differences in the founder mutations, genetic dispositions, and genetic testing of patients influence the detected frequency in any ethnic group.

A notable feature is observed in the mutation spectra of *BRCA1/2*, specifically its association with ethnicity. Over the past few years, researchers have identified numerous recurrent pathogenic *BRCA1/2* variants that originated from common ancestors, known as founder mutations, which are present in individuals from specific ethnicities or geographical areas. For instance, three founder mutations (BRCA1 c.68_69 delAG, *BRCA1* c.5266dupC, and *BRCA2* c.5946delT) in the Ashkenazi Jewish population are highly prevalent, with a carrier frequency of 2% in that population [10]. Similarly, other groups such as Icelander, French Canadian, Dutch, and Slavic populations of Eastern Europe have their characteristic mutations underscoring the strong founder effect in that geographical area [10]. Recurrent SNPs such as c.181T > G (p.Cys61Gly) are observed in Central/Eastern European, and many other common mutation variants are observed in certain populations, like those of Mexican Americans, etc. [10]. In groups like the South African population, at least five BRCA founder variants are reported across major ethnic lineages, including.1374del, *BRCA1* c.2641G > T, *BRCA2* c.7934del, BRCA2 c.5771_5774del, and *BRCA2* c.582G > A [11–13]. Moreover, recent studies demonstrated that approximately 38% of pathogenic *BRCA1/2* variants noted in a large Chinese group were unique to that population [14].

In summary, *BRCA1/2* mutations are clinically vital in BC and display remarkable variation in frequency across global populations. Evidence suggests that ethnicity can impact not only the probability of carrying a BRCA mutation but also the specific mutation present. Comprehending these ethnic differences is crucial for improving genetic risk assessment, tailoring a personalized screening approach, and ensuring that diagnostic tests and prophylactic interventions are suitably directed to diverse populations. However, the current literature exhibits that many populations remain underrepresented in BRCA research, leaving gaps in the picture of global mutation patterns. Therefore, the present systematic review is undertaken to comprehensively examine *BRCA1* and *BRCA2* mutations in BC patients globally, with the primary aim of identifying the ethnic association of BRCA variants SNPs across diverse populations. By thoroughly collecting and assessing data from studies conducted globally, we aim to clarify how the prevalence of *BRCA1/2* mutation correlates with ethnicity and to offer evidence-based insights that can inform more justifiable genetic testing and personalized therapy in BC patients.

## Materials and methods

### Study design and PRISMA guidelines

This study was designed to explore the prevalence of *BRCA1* and *BRCA2* variants across BC patients of diverse ethnicities. The study adhered to the Preferred Reporting Items for Systematic Reviews and Meta-Analyses (PRISMA) 2020 guidelines to ensure methodological accuracy, transparency, and reproducibility. The primary aim was to calculate the prevalence of *BRCA1* and *BRCA2* variants, focusing on SNPs, and to categorize these variants based on prevalence thresholds to guide clinical and research management.

### Search strategy and data sources

**Fig. 1:**
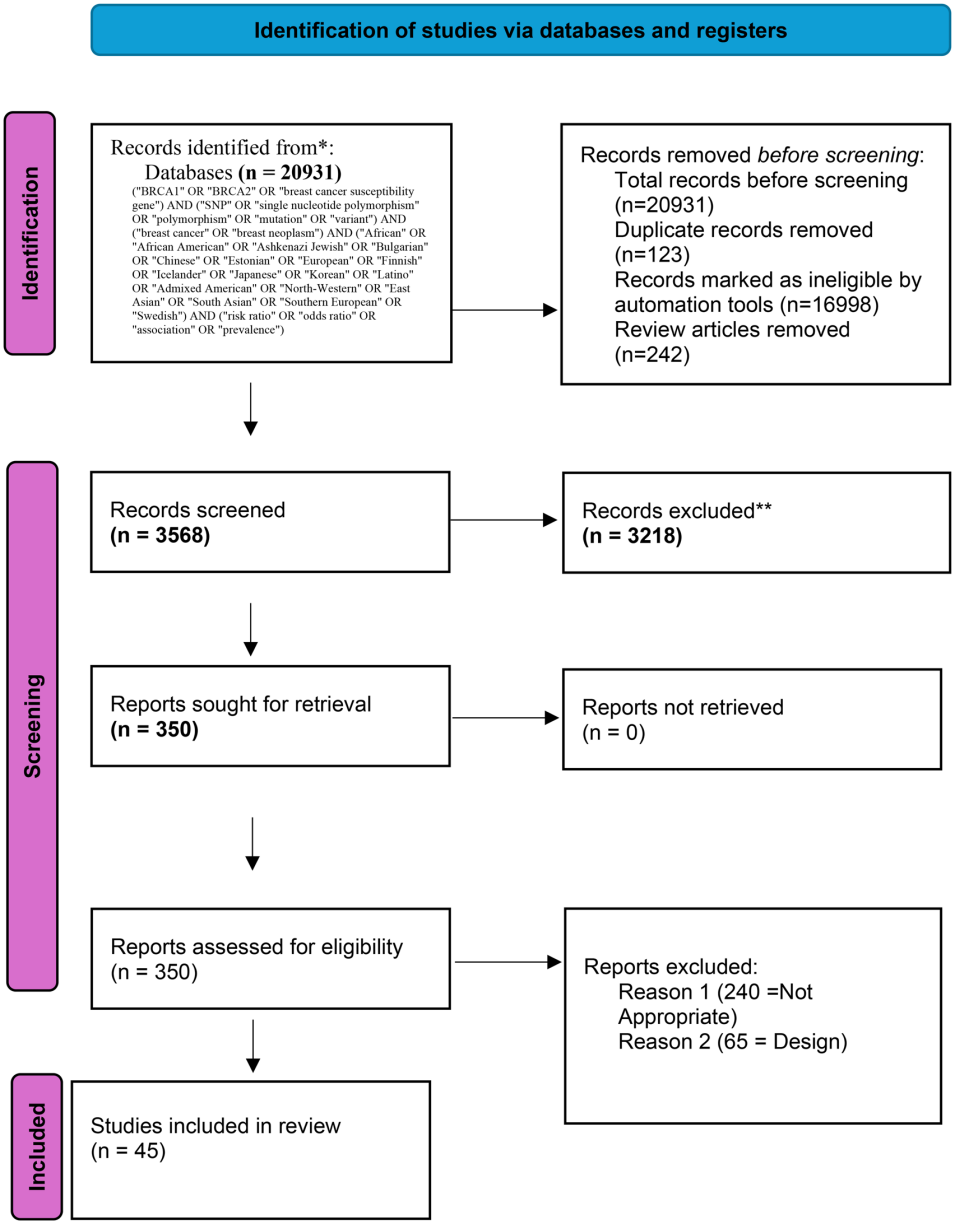
PRISMA flow chart

A systematic literature search was performed across PMC, PubMed, Embase, Scopus, and Web of Science databases from June 20, 2015, to June 20, 2025, to identify relevant articles. The search strategy utilized a combination of Medical Subject Headings (MeSH) and free-text terms, including “*BRCA1*,” “*BRCA2*,” “breast cancer susceptibility gene,” “mutation,” “SNP,” “single nucleotide polymorphism,” “polymorphism,” and “variant” for genetic factors; “breast cancer” and “breast neoplasm” for the disease; and ethnic/geographic terms such as “African,” “African American,” “Ashkenazi Jewish,” “Bulgarian,” “Chinese,” “Estonian,” “European,” “Finnish,” “Icelander,” “Japanese,” “Korean,” “Latino,” “Admixed American,” “North-Western,” “East Asian,” “South Asian,” and “Southern European.” Outcome-related terms included “risk ratio,” “odds ratio,” “association,” and “prevalence.” Boolean operators (AND, OR) were used to combine terms, with filters applied to restrict results to peer-reviewed, English-language studies involving human subjects. The search identified 20,931 records, which were manually screened from reference lists of included studies and relevant reviews to ensure comprehensive coverage, as documented in the PRISMA flow chart (Fig. 1).

### Inclusion and exclusion criteria

The studies were deemed eligible if they were published in the English language, peer-reviewed, original research articles containing case control, cohort, or cross-sectional studies, that reported *BRCA1* or *BRCA2* mutations in breast cancer patients and provided information regarding frequency and prevalence, odds ratio, SNP associations stratified by ethnic or geographical populations. Exclusion criteria comprised non-human or in vitro studies, review articles, meta-analyses, commentaries, letters to the editor, protocols, case reports, or studies not comprising specific information on *BRCA1/BRCA2* mutations or ethnic/geographic details. After removing the duplicate article (*n* = 123), automated tools excluded 16998 records based on the title and abstracts of the articles, followed by the exclusion of 242 review articles, resulting in 3568 records for full text eligibility. Thereafter, 3568 records were subjected to manual screening for titles and abstracts. Of these, a total of 3218 records were excluded due to their irrelevance. The remaining full-text 350 articles were assessed based on the inclusion criteria. No records were excluded due to full-text retrieval issues. The full text articles were thoroughly read and evaluated, and 305 studies were excluded, 240 articles due to inappropriate focus (no prevalence or frequency information of *BRCA1* and *BRCA2* mutation or association with ethnicity and 65 due to the study design (no mention of SNPs or an appropriate number of mutation carriers). Finally, 45 articles were included in the current systematic review that met all the eligibility criteria (Supplementary Table S1).

### Data extraction process

Data was extracted by two author reviewers via a standardized electronic form developed in R (version 4.4.1) to ensure the information is consistent with minimal errors. Extracted information included study characteristics (author, publication year, country, abstract, sample size), population details (ethnicity, geographic area), *BRCA1/BRCA2* mutation info (mutation type, e, g. Missense, frameshift etc., mutation impact, pathogenic, likely pathogenic, benign, etc.) and risk of bias scores based on the Joanna Briggs Institute (JBI) Critical Appraisal Checklist. Any inconsistencies between the reviewers were resolved via consultation with a third author reviewer. R scripts were utilized to automate data organization into a structured CSV file, assisting in subsequent assessment and ensuring reproducibility.

### Methodological quality assessment

The risk of bias assessment tool, developed by Hoy et al. [15], was used to evaluate the quality of individual studies. The tool has 10 items: four items were related to external validity in terms of how well the study sample represents the study population, and the remaining six items were related to internal validity of the studies. Total quality scores for each study were obtained by summing the binary response scores (Yes = 1 or No = 0) that ranged from 0 to 10. As per Hoy et al. [15], scores above 7 indicated low risk of bias, while a score between 6 and 7 indicated moderate risk of bias. Those studies scoring less than 6 were considered to have a high risk of bias.

### Ethnicity mapping

The ethnicity data extracted from the studies were grouped using R. Reported ethnicities were classified into broader groups: Chinese (including Chinese, Han Chinese, Chinese Hakka), Asian (including Indian, Korean, Bangladeshi, Malaysian Sarawak, Mauritian), Black or African Descent (Black African, African American, Indian South African, Tanzanian, Kenya), Hispanic/Latino (including Mexican, Columbian/Columbia, Argentinian, Latin American, Brazilian), European (Bulgarian, Polish, Sicily), Middle Eastern/North African (including Lebanese, Saudi Arabian, Jordian, Iranian, Iraqi Kurdish, Bahraini, Moroccan, Omani, Tunisian). Jewish (including Ashkenazi Jewish), and Other Unclassified (including French Canadian, and Turkish). This ethnicity mapping was applied using R scripts with results organized in a CSV file for subsequent analysis (Table S2).

### Variant filtering for SNPs

The extracted data contains all the variants within *BRCA1* and *BRCA2*, which were filtered to focus on single nucleotide variants (SNVs), focusing exclusively on SNPs within both *BRCA1/2* variants. Non-SNP variants (e.g., insertions, deletions, frameshift variants) were excluded using bioinformatics tools in R software. The SNP Stats package recognized and mapped SNP prevalence across all ethnic groups.

### Variant classification and stratification

To ensure clinical validity while preserving the landscape of reported data, we adopted a two-tiered analysis strategy. For the initial landscape analysis, we retained the full dataset of reported variants, including Variants of Uncertain Significance (VUS) and high-frequency benign polymorphisms (e.g., BRCA1 c.3113A > G). This dataset illustrates the ‘raw’ frequency of genetic findings as reported in the literature and highlights potential misclassification trends. Additionally, for the estimation of cancer risk, the dataset was strictly filtered to include only variants classified as Pathogenic or Likely Pathogenic (PLP) (Class 4/5) according to ACMG/AMP guidelines and ClinVar. All benign polymorphisms and VUS were excluded from these specific estimates.

### Variant classification by prevalence

The SNPs were categorized on the basis of their prevalence in BC patients within each ethnic group using a predefined threshold framework to examine clinical and research relevance. The categories were standardized as follows to examine clinical relevance: Low (< 2%), Low-Moderate (2–4%), Moderate (5–29%), High (30–49%), and Extremely High (≥ 50%). These thresholds were applied to distinguish between sporadic variants and potential founder mutations or recurrent polymorphisms. Prevalence was calculated as a fraction of mutation carriers among BC patients per ethnic group, with results collected to emphasize the ethnic specificity and global patterns.

### Statistical analysis

Meta-analyses were conducted using R (version 4.4.1) with the meta and metafor packages. Pooled prevalence estimates were calculated using a random-effects model with the inverse variance method and logit transformation to account for heterogeneity. Heterogeneity was assessed using the I² statistic and Cochran’s Q test (I² >50% indicated substantial heterogeneity). Due to the inconsistent reporting of clinical covariates (e.g., ER/PR status, mean age, family history criteria) across the included studies, meta-regression with clinical variables was not feasible. Consequently, sources of heterogeneity were investigated primarily through subgroup analysis by ethnicity (e.g., Chinese, Asian, Black or African Descent, Hispanic/Latino, Middle Eastern/North African, Jewish) and geographic region. Additionally, data were pooled by broad ethnic category using a random-effects model. Stratification by specific recruitment criteria (e.g., separating high-risk from unselected cohorts) was not feasible for all ethnic groups due to the limited number of studies in underrepresented regions. The SNP Stats package mapped SNP distributions, with results exported to CSV files. Forest plots visualize pooled prevalence. Publication bias was assessed using funnel plots (Supplementary Fig. S1). Prediction intervals were not calculated as the high heterogeneity rendered them uninformative. Sensitivity analyses evaluated the impact of studies with a high risk of bias (JBI score < 5) on pooled estimates. All outputs were saved as CSV files for transparency.

## Results

### Characteristics of the study

The data were collected from 45 studies conducted between 2015 and 2025, investigating mutational variants including SNPs within *BRCA1* and *BRCA2* genes, across diverse ethnic populations. The studies provided 844 Mutation variants, comprising 447 *BRCA1* variants and 397 *BRCA2* variants, derived from 44,344 BC patients tested for *BRCA1* and 18,675 for BRCA2 mutations worldwide (Supplementary Tables S2 & S3). These studies employed various types of molecular detection techniques, including Sanger sequencing, Next-generation sequencing (NGS), Multiple ligation dependent Probe Amplification, and PCR-based assays, ensuring robust detection of BRCA variants. As shown in Table 1 (Also see Supplementary Table S4), the dataset included a wide range of ethnicities (classified under 8 broad ethnic populations; Asian, European, Black or African descendants, Middle eastern/North African, Hispanic/Latino, Ashkenazi Jewish, Chinese and others) and cohort sizes, from small ethnic groups (e.g., 3 Jewish patients tested for *BRCA1*) to a large group of 26,751 Chinese patients tested for the BRCA1 mutation. The data from these 45 studies were put into analysis to calculate the overall prevalence of BRCA1 and BRCA2 variants in BC patients across different ethnicities. A total of 402 Pathogenic/Likely Pathogenic (PLP) variants (195 BRCA1 and 207 BRCA2) were identified from the pooled cohort of 44,344 breast cancer patients. Note that while 844 total variants were reported across studies, approximately half were re-classified as benign polymorphisms or VUS and excluded from the primary clinical prevalence estimates.

**Table 1:** Original publications of *BRCA1/2* variation in different ethnicities

S. no.	Author	Ethnicity	Published year	Cases analyzed	Broad Ethnic Classification
1	Dodova RI et al. [16]	Bulgarian	2015	200	European
2	Francies FZ et al. [17]	South African	2015	108	Black or African descendants
3	El Saghir NS et al. [18]	Lebanese	2015	250	Middle eastern/North African
4	Villarreal-Garza C et al. [19]	Mexican	2015	190	Hispanic/Latino
5	Pal T et al. [20]	Black Women (Florida)	2015	369	Black or African descendants
6	Abdikhakimov A et al. [21]	Uzbek	2016	67	Other/Unclassified
7	Bu R et al. [22]	Saudi Arabia	2016	818	Middle eastern/North African
8	Yoon KA et al. [23]	Korean	2017	328	Asian
9	Ricks-Santi L et al. [24]	African American	2017	31	Black or African descendants
10	Walsh T et al. [25]	Ashkenazi Jewish	2017	3	Ashkenazi Jewish
11	Yang XR et al. [26]	Malaysian (Sarawak)	2017	467	Asian
12	Briceño-Balcázar I et al. [27]	Colombian	2017	853	Hispanic/Latino
13	Fang M et al. [28]	Chinese	2018	71	Chinese
14	Liang Y et al. [29]	Chinese	2018	595	Chinese
15	Abdel-Razeq H et al. [30]	Jordanian	2018	100	Middle eastern/North African
16	Shah ND et al. [31]	Indian	2018	79	Asian
17	Abulkhair O et al. [32]	Saudi Arabian	2018	310	Middle eastern/North African
18	Wang T et al. [33]	Chinese	2019	82	Chinese
19	Al Hannan F et al. [34]	Bahraini	2019	25	Middle eastern/North African
20	Khalili-Tanha G et al. [35]	Iranian (South Khorasan)	2019	40	Middle eastern/North African
21	Shen M et al. [36]	Chinese	2019	54	Chinese
22	Geredeli C et al. [37]	Turkish	2019	99	Other/Unclassified
23	Cortés C et al. [38]	Columbian	2021	201	Hispanic/Latino
24	Wu Y et al. [39]	Han Chinese	2020	23,481	Chinese
25	Mahfoudh W et al. [40]	Tunisia	2019	33	Middle eastern/North African
26	Millan Catalan O et al. [41]	Latin American	2019	252	Hispanic/Latino
27	Nishat L et al. [42]	Bangladeshi	2019	65	Asian
28	Behl S et al. [43]	French-Canadians	2020	555	European
29	Hur JY et al. [44]	Korean	2020	2720	Asian
30	Bakkach J et al. [45]	Morrocon	2020	33	Asian
31	Abu-Helalah M et al. [46]	Jordan	2020	192	Middle eastern/North African
32	Abdel-Razeq H et al. [30]	Jordan	2021	616	Middle eastern/North African
33	Solano AR et al. [47]	Argentina	2021	443	Hispanic/Latino
34	Szczerba E et al. [48]	Polish	2021	75	European
35	Bang YJ et al. [49]	Korean	2021	4215	Asian
36	Stella S et al. [50]	Sicily	2022	376	European
37	Brahim SM et al. [51]	Mauritania	2022	137	Asian
38	Rweyemamu LP et al. [52]	Tanzanian	2023	100	Black or African descendants
39	Melki R et al. [53]	Morocco	2023	184	Asian
40	Rioki JN et al. [54]	Kenya	2023	6	Black or African descendants
41	Zhang et al. [55]	Chinese Hakka	2023	72	Chinese
42	Yu S et al. [56]	Chinese	2024	2216	Chinese
43	Hassan AN et al. [57]	Iraqi Kurdish	2024	70	Middle eastern/North African
44	Al Amri WS et al. [58]	Omani	2024	1336	Middle eastern/North African
45	Rojas LXR et al. [59]	Colombian	2025	307	Hispanic/Latino

### Risk of Bias

**Fig. 2:**
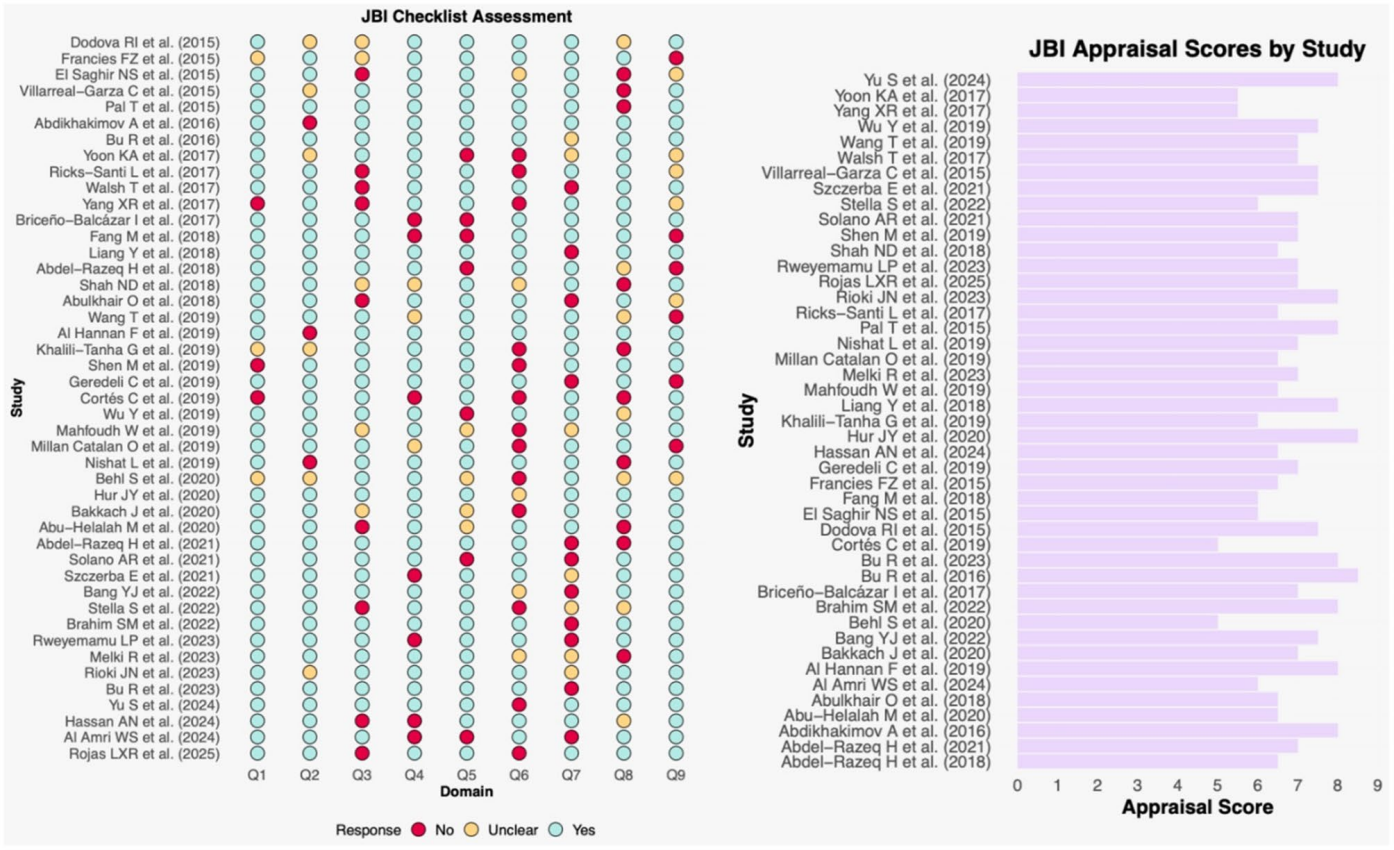
Risk of bias plot illustrating the methodological quality assessment of the 45 included studies

Of the 45 studies included in our study, 15 studies were found to have low risk of bias [16, 19–22, 29, 34, 39, 44, 48, 49, 51, 54–56], 26 had moderate risk of bias [17, 18, 24, 27, 28, 30, 30–33, 35–37, 40–42, 45–47, 50, 52, 53, 57–59], and 4 had high risk of bias [23, 26, 38, 43] (Fig. 2).

### Landscape of reported variant frequency of reported variants *BRCA1* and *BRCA2* across different ethnicities

**Fig. 3:**
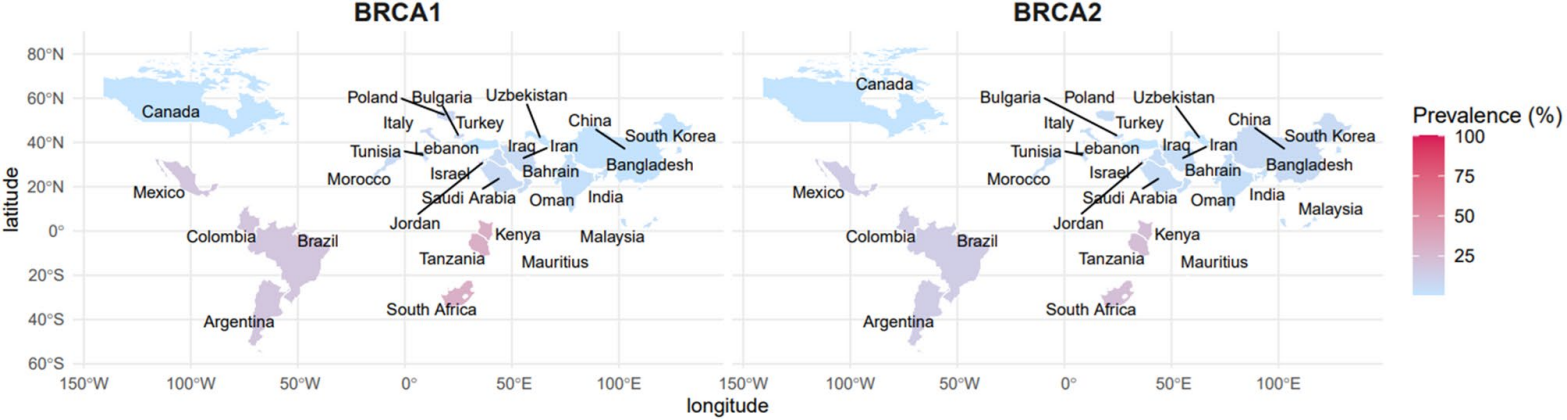
Geographical distribution of reported *BRCA* variant frequencies. Heatmap illustrating the density of reported genetic findings by region. Darker colors indicate higher reported frequencies, which may include population-specific polymorphisms

**Fig. 4:**
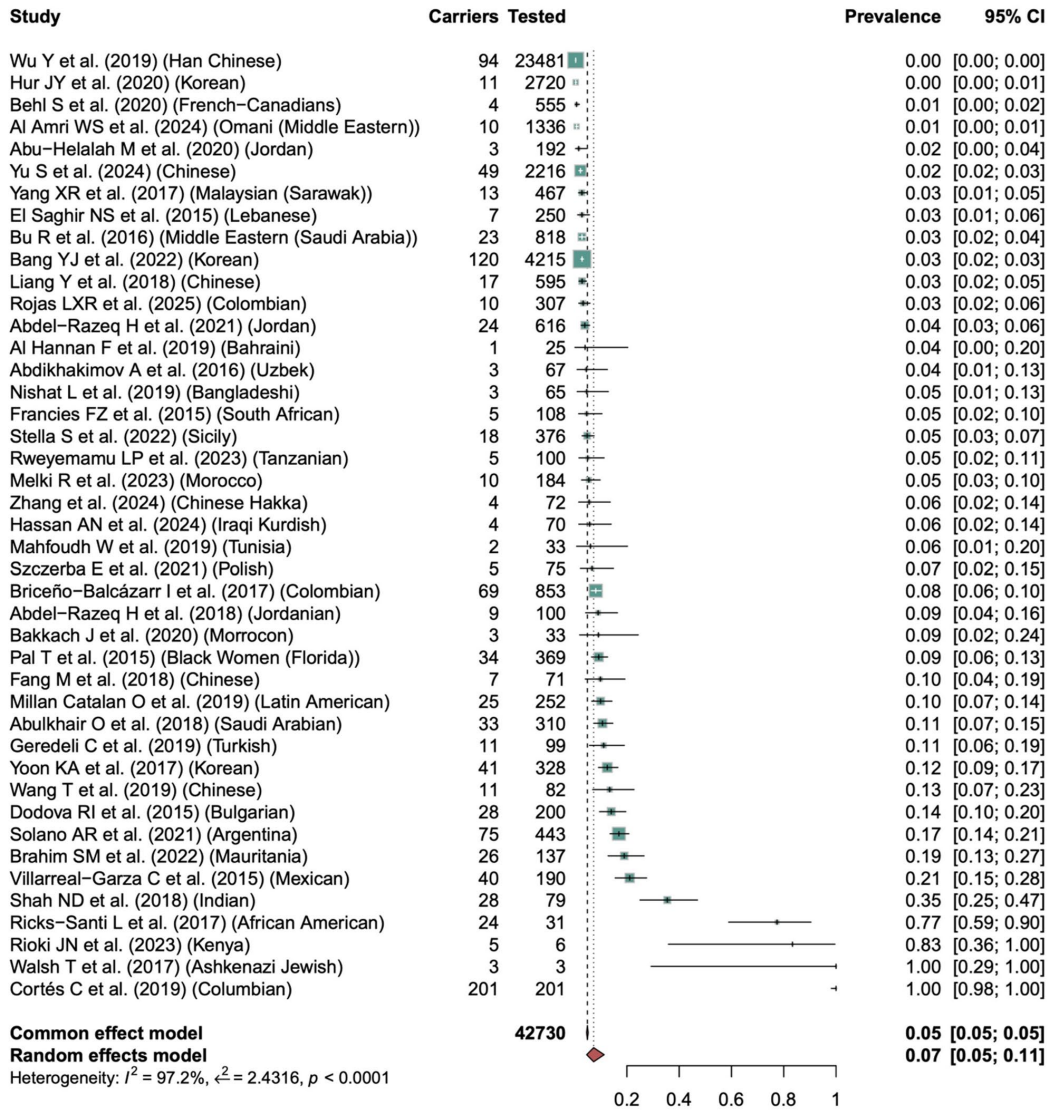
Frequency of all reported BRCA1 variants (Including Polymorphisms) across ethnic groups. Forest plot showing the pooled frequency of all variants reported in the included studies

**Fig. 5:**
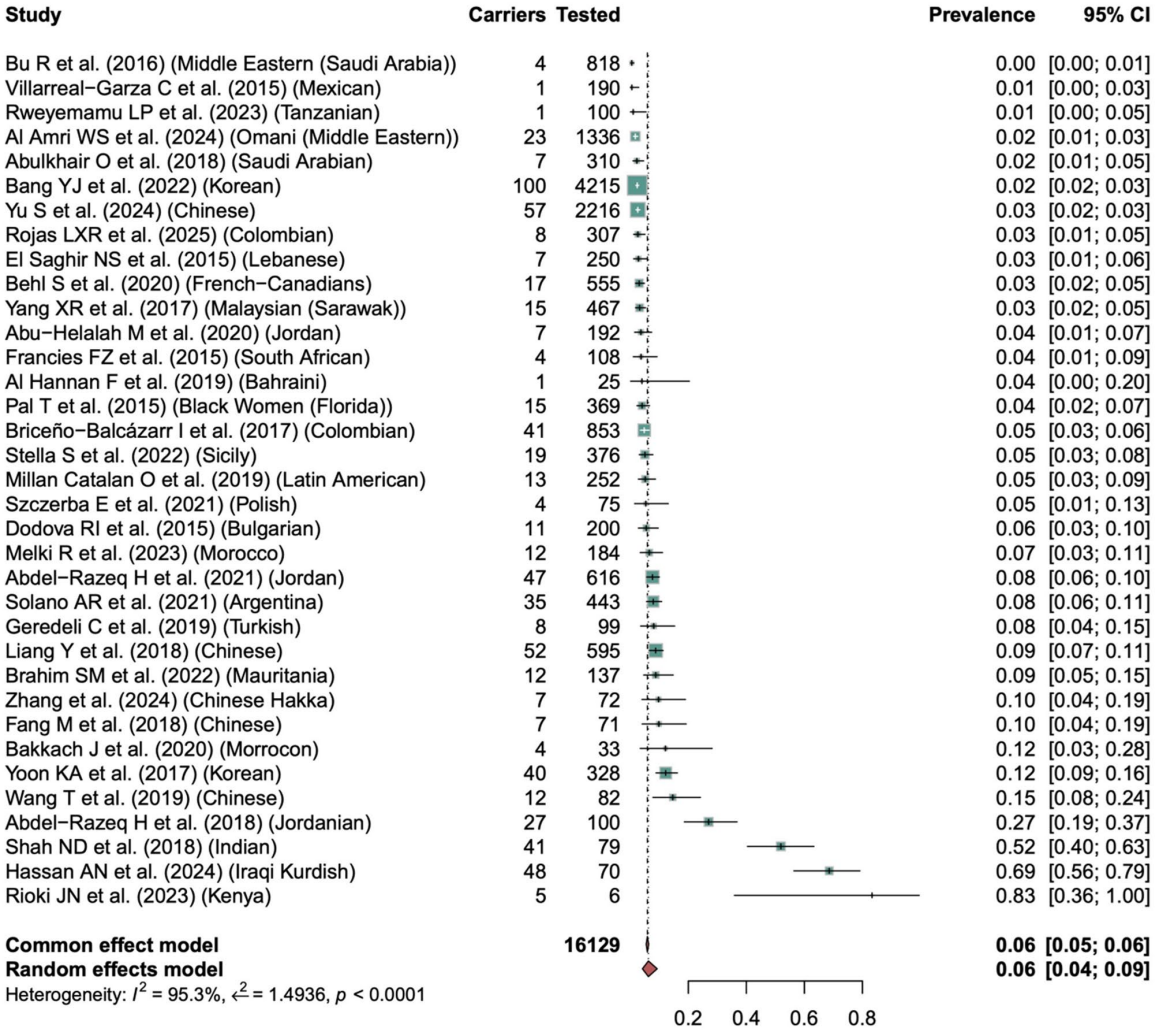
Frequency of all reported BRCA2 Variants (Including Polymorphisms) across ethnic groups. Forest plot showing the pooled frequency of all variants reported in the included studies

Data from 45 studies, covering diverse ethnic populations, were analyzed and represented as global map (Fig. 3) and forest plots (Figs. 4 and 5) showing the pooled frequency of all reported *BRCA1* and *BRCA2* variants. Figure 3 shows the global *BRCA1* and *BRCA2* mutation prevalence by geographical locations. These estimates ranged from < 1% in unselected cohorts to over 17% in specific subgroups (e.g., African or Asian studies). It is critical to note that these higher frequencies reflect the total burden of reported findings, which often includes common benign polymorphisms, and do not necessarily represent cancer risk.

As shown in Fig. 4, from studies reporting *BRCA1* mutation cases, the overall pooled prevalence of *BRCA1* among BC patients ranged widely from 0.00 to 1.00. The heterogeneity across studies was significant (I^2^ = 97.2%, τ² = 2.4316, *p* < 0.0001), indicating substantial variability in mutation frequency between populations. Most of the studies, especially those that involved large, unselected BC patients, reported low *BRCA1* prevalence rates below 5%. For instance, Behl S et al. (2020) reported a prevalence of 1% among Han Chinese, Abu Halalah M et al. (2020) reported 2% in Jordanians, and Abdikhakimov A et al. (2016) documented 4% in Uzbekians. Moderate prevalence (5–15%) was observed in the studies from South Africa, Colombia, Tunisia, Morocco, Jordan, etc., including Francies FZ et al. (2015), Zhang et al. (2024), Pal T et al. (2015), Dodova RI et al. (2015), Mahfoudh W et al. (2019), and Abulkhair O et al. (2018), indicating regional recurrence or founder effects. The highest prevalencerates were found in smaller, high-risk BC patients. For instance, Solano AR et al. (2021) reported a 21% *BRCA1* carrier rate among BC patients in Argentina, Brahmin SM et al. (2022) reported a 19% carrier rate in BC patients of Mexico, Shah ND et al. (2018) observed 35% in Indian cohort, Ricks Santi L et al. 2017 observed 77% in African American, Rioki JN et al. (2023) found 83% in Kenyan patients, and Walsh T et al. (2017) and Cortis C et al. (2019) reported a full 100% prevalence among Ashkenazi Jewish and Columbian cohorts respectively.

As shown in Fig. 5, the *BRCA2* variant prevalence analysis included 36 studies, and the overall pooled prevalence of *BRCA2* among BC patients ranged from 0.00 to 0.83. Similar to BRCA1, the heterogeneity across studies was substantial (I² = 95.3%, τ² = 1.4936, *p* < 0.0001), suggesting high variability in mutation frequency between populations. Most of the studies reported low *BRCA2* prevalence rates below 5%. For instance, Villarreal-Garza et al. (2015) reported a prevalence of 1% among Mexican BC patients, AI Amri WS et al. (2024) documented 2% in Omani, Yu S et al. (2024) found 3% in Chinese, and Francies FZ et al. (2015) reported 4% in South African. Moderate prevalence (5–15%) was found in the studies from Colombia, Sicily, Latin America, Bulgaria, Jordan, China, and Morocco etc., including Briceño-Balcázar I et al. (2017), Stella S et al. (2022), Millan Catalan et al. (2019), Dodova R et al. (2015), Abdel-Razeq et al. 2021, Bakkach J et al. 2020, and Wang T et al. (2019) indicating founder effects.

The highest prevalence rates were found in studies with small cohorts. For instance, Abdel-Razeq et al. (2018) reported a 27% *BRCA2* carrier rate among BC patients in Jordan, ShaH ND et al. (2024) observed 52% in an Indian cohort, Hassan AN et al. (2024) found 69% in Iranian and Rioki JN et al. (2023) observed 83% in Kenyan BC patients. These findings highlighted significant population-level variability in the prevalence of *BRCA1* and *BRCA2* variants, attributed to founder effects, regional ancestry, and cohort selection criteria.

### Overall prevalence of filtered *BRCA1* and *BRCA2* variants across ethnicities

**Fig. 6:**
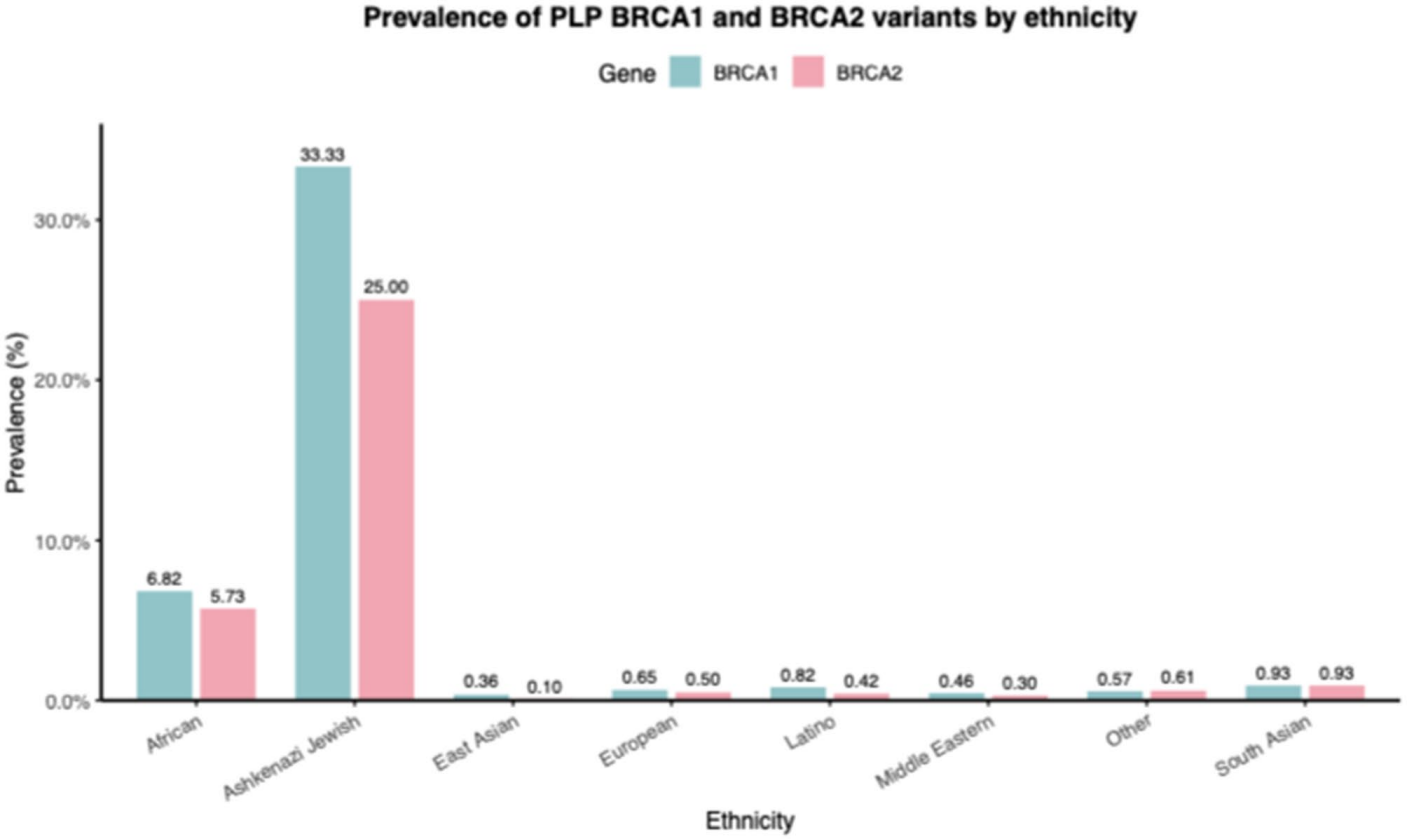
Estimated clinical prevalence of pathogenic/likely pathogenic (PLP) variants only. Bar chart summarizing the pooled prevalence after strict exclusion of benign polymorphisms and VUS. These estimates represent the clinically actionable mutation burden

The dataset was filtered to prioritize SNPs with pathogenic or likely pathogenic impacts and applied a threshold prevalence. The filtered dataset revealed significant ethnic variation in the prevalence of *BRCA1* and *BRCA2* SNPs, as summarized in (Supplementary Tables S5.1-S5.16). For *BRCA1* SNPs, 619 carriers were identified among 19,081 tested individuals, and for *BRCA2*, 617 carriers were identified among 14458BC patients. The overall pooled prevalence of *BRCA1* and *BRCA2* SNPs was calculated in the respective tested individuals and shown in Fig. 5. The Ashkenazi Jewish group displayed the highest prevalence of the *BRCA1* gene (100%) found in a small cohort of 3 BC patients. Black or African descendants showed high prevalence (17.26% for *BRCA1* SNP and 18.94% for *BRCA2* SNP), driven by pathogenic SNPs discussed later. Hispanic/Latino individuals also exhibited elevated prevalence for the *BRCA2* SNP, 11.86% but low prevalence for the *BRCA1* SNP, 2.15%. The Chinese and European populations exhibited a prevalence of 6.18% and 6.14% for *BRCA1*, while 3.51% and 1.11% for *BRCA2* SNPs. Remaining populations, including Asian, Middle Eastern/North African, and Other/Unclassified, demonstrated a prevalence of < 5% for both *BRCA1* and *BRCA2* SNPs (Supplementary Fig. S2). To further estimate the true clinical burden of hereditary breast cancer, we re-analyzed the data to strictly include only PLP (402) variants. As shown in Fig. 6, the true clinical prevalence was significantly lower, ranging from 0.3% to 6.8%, removing the inflation caused by non-pathogenic variants.

### Ethnic specific prevalence and SNP distribution

#### *BRCA1* variants prevalence

**Fig. 7:**
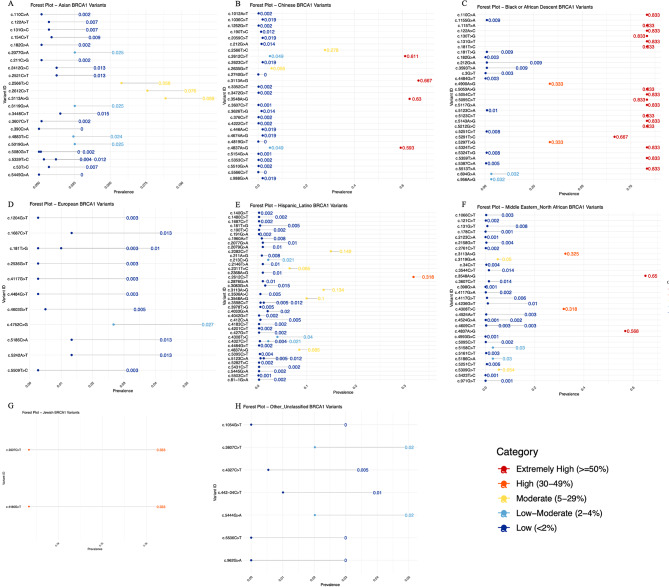
Forest Plot of *BRCA1* variants among various ethnicities. (**A**) Asian (**B**) Chinese (**C**) Black African or African descendants (**D**) European (**E**) Hispanic/Latino (**F**) Middle Eastern (**G**) Jewish (**H**) Unclassified

**Fig. 8:**
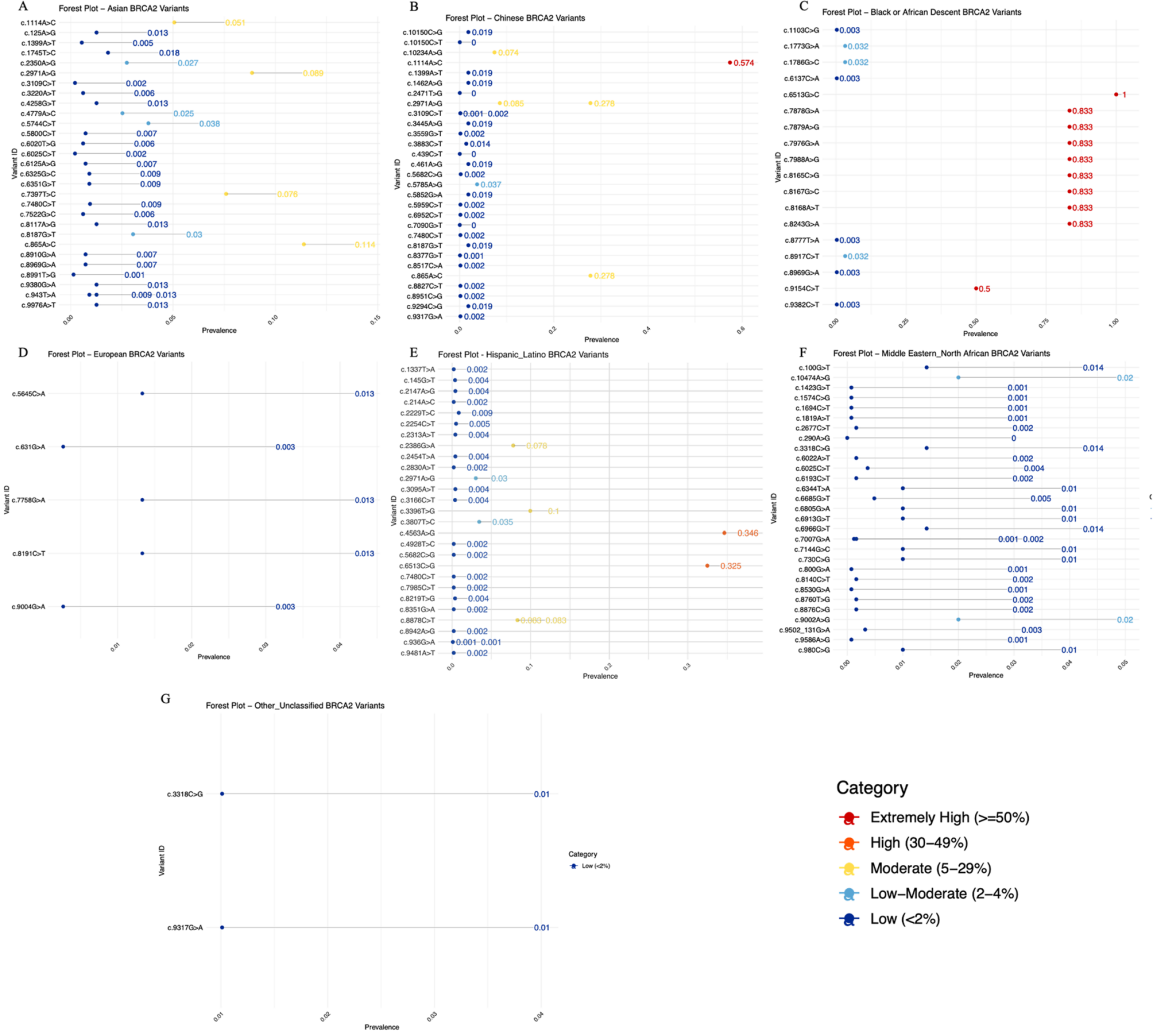
Forest Plot of *BRCA2* variants among various ethnicities. (**A**) Asian (**B**) Chinese (**C**) Black African or African descendants (**D**) European (**E**) Hispanic/Latino (**F**) Middle Eastern (**G**) Unclassified

The SNP distribution and ethnic specific prevalence were presented via forest plot analysis. A total of eight population-based forest plots for *BRCA1* and *BRCA2* SNPs were assessed, representing groups including Asian, Chinese, Black or African Descendants, European, Hispanic/Latino, Jewish, Middle Eastern/North African, and other populations (Figs. 7 and 8). Among BC patients of the Asian group, 24 Unique *BRCA1* SNPs were identified. The most frequent SNP variants included c.3113 A > G (8.9%), c.2612 C > T (7.6%), and c.2566T > C (5.8%), all falling within the moderate prevalence classification (5–29%) (Fig. 7A). Four variants that fell in the category of low-moderate (2–4%) included c.2077G > A, c.3119G > A, c.4883T > C, and c.5019G > A, all showing a prevalence of 2.5% while the remaining 16 SNP variants fell in the category of low (< 2%) prevalence.

As shown in Fig. 7B, among BC patients of the Chinese group, 29 SNPs were identified. The most prevalent SNP variants included c.2612 C > T (61.1%), c.3113 A > G (66.7%), c.3548 A > G (63.0%), and c.4837 A > G (59.3%), all falling under the extremely high category (≥ 50%). Two variants that fell in the category of moderate (5–29%) included c.2566T > C and c.2635G > T, showing a prevalence of 27.8% and 56% respectively. SNPs c.2612 C > T (4.9%) and c.4837 A > G (4.9%) were also seen at low-moderate (2–4%) prevalence levels. The remaining 23 SNP variants fell in the category of low (< 2%) prevalence.

A total of 32 *BRCA1* SNPs were documented in Black African or African descendants. As shown in Fig. 7C, the prevalence of 18 SNPs is extremely high (≥ 50%), suggesting strong founder effects; however, the cohort was very small, containing only 6 BC patients. Two variants that fell in the category of high (30–49%) included c.4900 A > G and c.5297T > G, both showing a prevalence of 33%. Two additional variants, including c.694G > A and c.956 A > G, both showing a prevalence of 3.2%. The remaining 8 SNP variants fell in the category of low (< 2%) prevalence.

In the European group of patients, 12 *BRCA1* SNPs were identified. Most of the variants fell in the category of low (< 2%) including c.181T > G, c.4603G > T, c.1687 C > T, c.5186 C > A, c.5242 A > T, c.1204G > T, c.2536G > T, c.4117G > T, c.4484G > T, c.5509T > C, while two variants fell in the *Low–Moderate* (2–4%) category, includingc.4752 C > G with a prevalence of 2.7% (Fig. 7D).

In the Hispanic/Latino population, a total of 43 *BRCA1* SNPs were identified. One variant, c.2612 C > T (31.8%), fell in the category of high prevalence (30–49%), while 5 other variants like c.2082 C > T (14.9%), c.2311T > C (6.5%), c.3113 A > G (13.4%), c.3548 A > G (10%), and c.4837 A > G (8.5%) fell in the category of moderate (5–29%) prevalence (Fig. 7E).

Further, in the dataset of the Middle Eastern population, 33 variants were identified. Two variants that fell in the extremely high prevalence (≥ 50%), included c.3548 A > G (65%) and c.4837 A > G (56.8%). Additionally, two variants that fell in the High prevalence category (30–49%) included c.3113 A > G and c.4308T > C, showing prevalence of 32.5% and 65% respectively. Two variants, including c.3119G > A and c.5309G > T, had a moderate prevalence (5%), and two other variants, like c.5158 C > T and c.5186 C > A, both had a low-moderate prevalence of 3%. The remaining 25 SNPs had a prevalence < 2% suggesting high allelic diversity (Fig. 7F).

In the Jewish cohort of 3 individuals, only two *BRCA1* SNPs were found, including c.4480G > T and c.3607 C > T, both showing a prevalence of 33.3%, placing them in the *High* category (30–49%). These variants probably reflect known Ashkenazi Jewish founder mutations (Fig. 7G).

Seven variants were reported in unclassified populations, out of which two variants, including c.962G > A and c.5536 C > T, fell under *Low–Moderate* (2–4%) and the remaining 5 under the low prevalence category (Fig. 7H).

#### *BRCA2* variants prevalence

Among BC patients of the Asian group, 30 Unique *BRCA2* SNPs were identified. As shown in Fig. 8A, the most frequent SNPs included c.2971 A > G (87%), c.865 A > C (11.3%), and c.7397T > C (7.5%) and c.1114 A > C (5.0%), all falling within the moderate prevalence classification (5–29%). Four variants that fell in the category of low-moderate (2–4%) included c.2350 A > G, c.8187G > T, c.4779 A > C, and c.5744 C > T, while the remaining 22 SNP variants fell in the category of low (< 2%) prevalence.

As shown in Fig. 8B, among BC patients of the Chinese group, 31 SNPs were identified. The most prevalent SNP variant included c.1114 A > C (57.4%) fell under the extremely high category (≥ 50%). Four variants that fell in the category of moderate (5–29%) included c.2971 A > G, c.10,234 A > G, c.2971 A > G and c.865 A > C, showing a prevalence of 8.5%, 7.4%, 27.7, and 27.7% respectively. SNPs c.5785 A > G (3.7%) were seen at low-moderate (2–4%) prevalence levels. The remaining 23 SNP variants fell in the category of low (< 2%) prevalence.

A total of 18 *BRCA2* SNPs were documented in Black African or African descendants. As shown in Fig. 8C, the prevalence of 10 SNPs was extremely high (≥ 50%) including c.7878G > A, c.8243G > A, c.7976G > A, c.8165 C > G, c.8167G > C, c.8168 A > T, c.7988 A > G and c.7879 A > G, all showing the prevalence of 83.3%, c.9154 C > T showing the prevalence of 50% and c.6513G > C showing the prevalence of 100%, suggesting strong founder effects; however, the cohort was very small, containing only 6 BC patients. Two variants that fell in the category of low-moderate (5–29%) included c.4900 A > G, c.1786G > C, and c.8917 C > T, both showing a prevalence of 3.2%. The remaining 5 SNP variants fell in the category of low (< 2%) prevalence.

In the European group of patients, 5 BRCA2 SNPs were identified. All the variants fell in the category of low (< 2%), including c.8191 C > T, c.5645 C > A, c.7758G > A, c.631G > A, and c.9004G > A (Fig. 8D).

In the Hispanic/Latino population, a total of 29 *BRCA2* SNPs were identified. Two variant, c.4563 A > G (34.6%) and c.6513 C > G (32.4%) fell in the category of high prevalence (30–49%), while 3 other variants like c.2386G > A (7.7%), c.3396T > G (10%), and c.8878 C > T (8.3%) fell in the category of moderate (5–29%) prevalence. Two other variants, like c.2971 A > G and c.3807T > C, fell in the category of low-moderate 2–4% prevalence. The remaining 21 variants fell in the low category of < 2% prevalence (Fig. 8E).

Further, in the dataset of the Middle Eastern population, 30 SNPs were identified. Only two variants fell in the low-moderate prevalence (2–4%), including c.9002 A > G and c.10,474 A > G, both showing a prevalence of 3%. The remaining 28 SNPs had a prevalence < 2% (Fig. 8F).

Two variants, c.3318 C > G and c.9317G > A, were reported in unclassified populations, and both fell under the low prevalence category (Fig. 8G).

## Discussion

The results of this systematic review and meta-analysis delineate significant ethnic variability in the prevalence and pattern of *BRCA1* and *BRCA2* mutations among breast cancer patients. Our analysis distinguishes between high-frequency benign polymorphisms and pathogenic founder variants. While common polymorphisms (e.g., BRCA1 c.3113 A > G) dominate the prevalence statistics in unselected Asian cohorts, our data highlights specific pathogenic candidates in African and Hispanic populations that are currently underrepresented in standard Western diagnostic panels. For example, BRCA1 c.3113 A > G and c.2612 C > T each appear in > 7% of Asians in our data; however, clinical databases such as ClinVar classify c.3113 A > G as a benign polymorphism with an allele frequency of ~ 33% in Asians [60], rather than a cancer-driver mutation. This means that although the variant is not pathogenic, its impact on cancer risk is not fully understood and requires further validation. Further, the *BRCA2* c.865 A > C observed in our forest plots is also benign with ~ 10% Asian frequency [61]. In other words, these “top hits” in prevalence are consistent with common SNPs in public databases. *BRCA1* c.3113 A > G is not only prevalent in the Asian population, but also the most frequent variant found in the Chinese population in the present study. These findings align with other literature, where it is the most frequently found variant in patients with early-onset breast cancer and is a previously identified mutation “hotspot” in the Chinese population [62]. Interestingly, recent studies on cancer mutational hotspots indicate that recurrent mutations might impact tumor behavior and patient outcomes. A large analysis of > 1400 cancer hotspots, including those of *BRCA1/2*, displayed that *some* recurrently mutated positions are linked with differential patient survival, accentuating that even common mutations might have functional relevance in tumors [63]. Further,*c.2635G > T in BRCA1* is the most prevalent variant found in the Chinese population in this study and is well documented as pathogenic as per the ClinVar and other databases [64, 65]. Clinically, this variant is considered a known disease-causing mutation associated with hereditary breast/ovarian cancer. These findings are consistent with other studies that this pathogenic variant is a known mutation in BC patients from Hong Kong Chinese, Malaysia, and Singapore [66–70]. Similarly, c.1114 A > C in *BRCA2* is the most common *BRCA2* variant in our dataset and, while historically deliberated, at least one ClinVar submitter has categorized it as pathogenic/likely pathogenic, with others cataloguing it nonpathogenic [71]. These well-documented events reinforce the importance of considering the demographic history of a population in interpreting patterns of BRCA mutation prevalence. The clinical relevance of this result is considerable, as many commercially available gene panels used globally are derived from mutation frequencies established in Western populations, thereby reducing their sensitivity and utility for Asian patients. This underscores the necessity of tailoring genetic testing strategies and interpretive frameworks to the molecular epidemiology of each population.

In contrast, data from Black and African-descent populations revealed comparatively high prevalences of *BRCA2* pathogenic variants, notably including recurrent alleles such as c.6513G > C. However, the overall picture in these populations remains less well defined, partly due to a disproportionate focus of available data on South African cohorts while large regions of sub-Saharan Africa, West Africa, and African diaspora populations remain understudied [72]. Recent studies emphasize that mutation profiles in African populations show significant heterogeneity that may be attributable to distinct ancestral backgrounds and a complex history of migration and admixture [73]. Additionally, literature indicates that socioeconomic factors and disparities in healthcare access further modulate the impact of genetic risk in these communities [74].

In our data, the single very-high prevalence variant is c.2612 C > T (31.8% in BRCA1), which is not a well-known founder; however, its high frequency indicates it may be a population-specific SNP or a private founder. The moderate-frequency variants (e.g. *BRCA1* c.2082 C > T, c.4837 A > G) likewise may represent recurrent alleles or polymorphisms. Indeed, almost half of the mutations in large Hispanic cohorts are accounted for by a handful of recurrent alleles [75]. Our *BRCA1* findings are broadly consistent with this pattern: multiple variants appear at intermediate frequency, possibly reflecting recurrence in Latino lineages. Regional studies in Latin America confirm this diversity in mutation spectra and emphasize the need for region-specific variant databases and risk models [76]. These findings have important implications for the design of screening protocols and genetic counseling in multi-ethnic societies.

The considerable heterogeneity identified in this meta-analysis (I² >95%) reflects limitations that are widely recognized in the scientific literature and are not specific to any single investigation. The considerable heterogeneity identified in this meta-analysis (I² >95%) reflects limitations that are widely recognized in the scientific literature. Notably, extreme prevalence estimates observed in specific subgroups (e.g., Ashkenazi Jewish and Kenyan cohorts) must be interpreted with caution due to small sample sizes (n < 10). While these studies were retained to ensure systematic completeness and highlight the presence of specific variants, they do not statistically represent broader population-level prevalence and should not be used to infer general population risk without validation in larger cohorts. Common sources of variation include differences in cohort selection, such as whether studies enroll high-risk familial cases or unselected population samples, which can lead to divergent prevalence estimates. Variation in sequencing technologies and analytic sensitivity, next-generation sequencing often detects a broader range of variants than conventional methods, also introduces inconsistency across studies. Additionally, the necessity of pooling data into broad ethnic categories (e.g., ‘Asian’ or ‘African Descent’) for statistical power inherently masks distinct sub-population differences and micro-regional founder effects, such as those seen between specific diverse linguistic groups in Africa. Lastly, we acknowledge that pooling data from studies with varying recruitment criteria (e.g., unselected breast cancer patients vs. high-risk familial cohorts) introduces inherent heterogeneity. Stratification by sub-cohort was not feasible for all ethnic groups due to data scarcity in underrepresented regions. Therefore, the pooled estimates in this study should be interpreted as a composite measure of variant burden across study types, rather than a precise population-level penetrance estimate. Addressing these issues will require greater standardization in future study designs, analytic methodologies, and the reporting of population structure, as well as targeted efforts to fill persistent data gaps from underrepresented populations, particularly in Africa, Oceania, and Indigenous Americas.

Collectively, our study affirms that ethnicity is a primary determinant of both the distribution and frequency of pathogenic *BRCA1* and *BRCA2* variants. The implications of these findings are substantial for clinical practice. Population-specific founder mutations justify targeted screening strategies in some high-risk groups, while in others, more universal or polygenic approaches may be indicated. Furthermore, distinguishing between these high-frequency benign variants and true pathogenic founders is critical for selecting appropriate therapeutic agents, such as PARP inhibitors, which are indicated specifically for patients with deleterious germline BRCA mutations. Additionally, our results underscore the necessity of incorporating diverse population data into international variant databases and clinical interpretation frameworks, as echoed by recent recommendations from the American College of Medical Genetics and Genomics and the ClinGen Expert Panel [77, 78] Achieving equity in the genetic diagnosis and management of hereditary breast cancer requires continued international collaboration, more inclusive research, and dynamic models that integrate both genetic and non-genetic factors relevant to cancer risk. To aid in the clinical translation of these findings, Table 2 summarizes the key recurrent and founder variants identified in this review. Table 2A highlights pathogenic founder variants that should be prioritized in region-specific genetic screening panels to improve diagnostic yield. Conversely, Table 2B lists high-frequency benign polymorphisms identified in our analysis; distinguishing these from pathogenic drivers is essential to prevent false-positive misclassification in diverse populations.

**Table 2:** Translationally relevant BRCA variants by population: (A) founder/recurrent pathogenic variants for panel design, and (B) common benign polymorphisms to reduce misclassification

A. Founder / recurrent Pathogenic variants (panel-priority candidates)				Prevalence / Notes
Population / Ethnicity	Gene	Variant (c. nomenclature)	Clinical Significance / Evidence	
Ashkenazi Jewish	*BRCA1*	c.68_69delAG	Pathogenic (Founder)	100% in specific cohorts; widely recognized founder.
Ashkenazi Jewish	*BRCA1*	c.5266dupC	Pathogenic (Founder)	Essential for Jewish population screening panels.
Ashkenazi Jewish	*BRCA2*	c.5946delT	Pathogenic (Founder)	Essential for Jewish population screening panels.
Chinese	*BRCA1*	c.2635G > T	Pathogenic (ClinVar)	56.0% in specific Chinese cohorts^6^; a known hotspot.
North African (Morocco)	*BRCA1*	c.5309G > T	Pathogenic (Founder)	Recurrent in Northeastern Morocco; priority for regional testing.
North African (Tunisia)	*BRCA1*	c.5266dupC (5382insC)	Pathogenic	Observed in Tunisian TNBC cases; historically linked to multiple populations.
South African	*BRCA2*	c.7934del	Pathogenic (Founder)	Part of the unique South African founder set.
South African	*BRCA1*	c.2641G > T	Pathogenic (Founder)	Recurring lineage-specific variant.
**B. High-frequency benign polymorphisms (excluded from pathogenic risk assessment)**				
**Population / Ethnicity**	***Gene***	**Variant (c. nomenclature)**	**ClinVar Classification**	**Prevalence in Cohort (Analysis)**
Chinese / Asian	*BRCA1*	c.3113 A > G	Benign / VUS 11	66.7% in Chinese; 8.9% in broad Asian cohorts.
Chinese / Asian	*BRCA1*	c.2612 C > T	Benign	61.1% in Chinese; 7.6% in broad Asian cohorts.
Chinese / Asian	*BRCA1*	c.4837 A > G	Benign / Polymorphism	59.3% in Chinese; 56.8% in Middle Eastern.
Asian / Chinese	*BRCA2*	c.1114 A > C	Benign / Conflicting	57.4% in Chinese; 5.0% in broad Asian cohorts.
Hispanic/Latino	*BRCA2*	c.4563 A > G	Benign / Polymorphism	34.6% in Hispanic cohorts.
Middle Eastern	*BRCA1*	c.3548 A > G	Benign / Polymorphism	65.0% in Middle Eastern; 63.0% in Chinese.

## Conclusion

An in-depth understanding of genetic diversity, particularly involving *BRCA1* and *BRCA2*, remains fundamental for addressing hereditary breast cancer risk in clinical practice. Incorporating ancestry-specific insights enables more accurate genetic counseling, risk assessment, and patient-centered management. Broader inclusion of understudied populations within genomics research is needed to strengthen the relevance and precision of emerging guidelines, drive innovation in screening, and optimize therapeutic strategies. Advancing equity in cancer genetics will require ongoing scientific collaboration, improvements in data standardization, and consistent integration of diverse genomic datasets into clinical care. These steps are essential for translating research advancements into practical, widely accessible benefits for all individuals at risk for hereditary breast cancer.

## Supplementary Information

Below is the link to the electronic supplementary material.

Supplementary Material 1

Supplementary Material 2

Supplementary Material 3

Supplementary Material 4

Supplementary Material 5

Supplementary Material 6

Supplementary Material 7

## Data Availability

Not applicable.
