# Supplementary material for: Global prevalence and ethnic variation of pathogenic BRCA1/2 variants in breast cancer: a systematic review and meta-analysis
Source: J Transl Med. 2026 Mar 12;24:555. doi: 10.1186/s12967-026-07997-3 (PMC13097826; doi:10.1186/s12967-026-07997-3)
Supplement: Supplementary file 1 — Supplementary Material 1 [file 12967_2026_7997_MOESM1_ESM.docx]

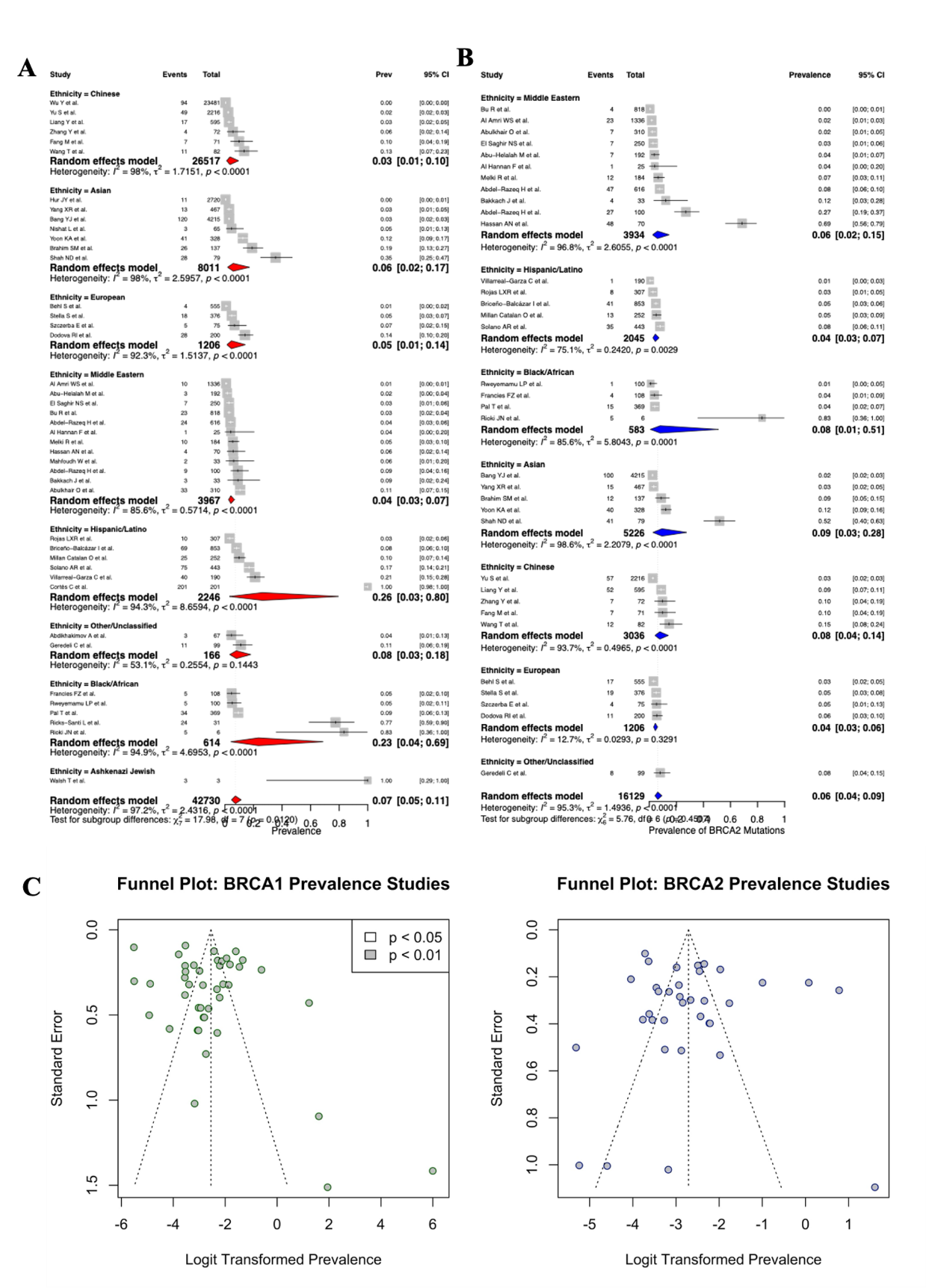


**Supplementary Figure S1. Funnel Plots for *BRCA1* and *BRCA2* Meta-Analyses.** Scatter plots of study size (standard error) against reported variant frequency (logit transformed). Asymmetry in the plots reflects the substantial heterogeneity in study design and variant reporting standards across the included literature.
