# Supplementary material for: Global prevalence and ethnic variation of pathogenic BRCA1/2 variants in breast cancer: a systematic review and meta-analysis
Source: J Transl Med. 2026 Mar 12;24:555. doi: 10.1186/s12967-026-07997-3 (PMC13097826; doi:10.1186/s12967-026-07997-3)
Supplement: Supplementary file 2 — Supplementary Material 2 [file 12967_2026_7997_MOESM2_ESM.docx]

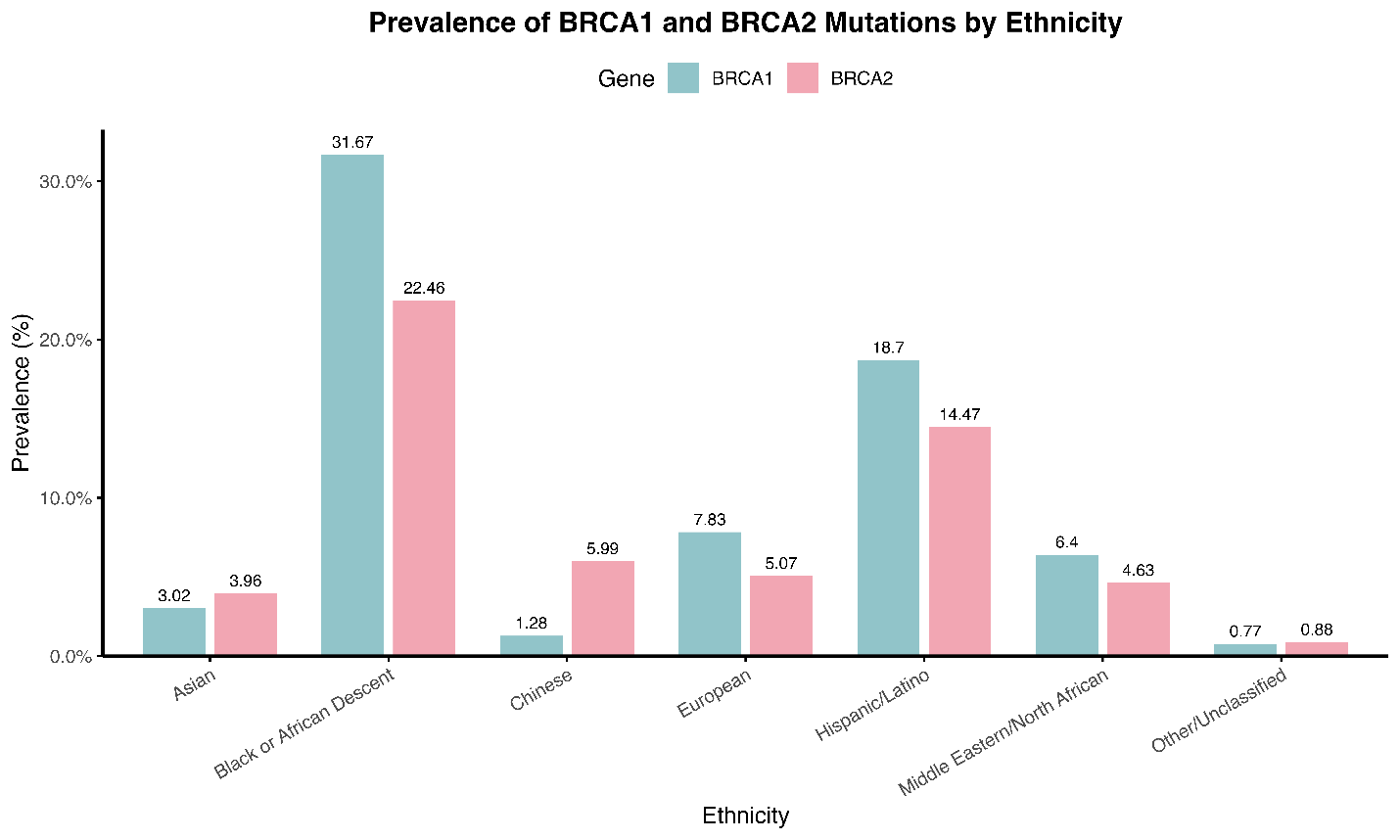


**Supplementary Figure S2.** Aggregated frequency of all reported *BRCA1/2* Variants by ethnicity. This bar chart summarizes the pooled frequency of all genetic variants as reported in the included studies across broad ethnic categories.
