# Supplementary material for: Global prevalence and ethnic variation of pathogenic BRCA1/2 variants in breast cancer: a systematic review and meta-analysis
Source: J Transl Med. 2026 Mar 12;24:555. doi: 10.1186/s12967-026-07997-3 (PMC13097826; doi:10.1186/s12967-026-07997-3)
Supplement: Supplementary file 3 — Supplementary Material 3 [file 12967_2026_7997_MOESM3_ESM.docx]

**Supplementary Table S1. Details of Studies included in the systematic literature review**

| **S.No.** | **First Author** | **Title** | **Publication Year** |
| --- | --- | --- | --- |
| 1 | Dodova RI | spectrum and frequencies of brca1/2 mutations in bulgarian high risk breast cancer patients | 2015 |
| 2 | Francies FZ | brca1, brca2 and palb2 mutations and chek2 c.1100delc in different south african ethnic groups diagnosed with premenopausal and/or triple negative breast cancer | 2015 |
| 3 | El Saghir NS | brca1 and brca2 mutations in ethnic lebanese arab women with high hereditary risk breast cancer | 2015 |
| 4 | Villarreal-Garza C | the prevalence of brca1 and brca2 mutations among young mexican women with triple-negative breast cancer | 2015 |
| 5 | Pal T | a high frequency of brca mutations in young black women with breast cancer residing in florida | 2015 |
| 6 | Abdikhakimov A | the potential contribution of brca mutations to early onset and familial breast cancer in uzbekistan | 2016 |
| 7 | Bu R | identification of novel brca founder mutations in middle eastern breast cancer patients using capture and sanger sequencing analysis | 2016 |
| 8 | Yoon KA | clinically significant unclassified variants in brca1 and brca2 genes among korean breast cancer patients | 2017 |
| 9 | Ricks-Santi L | next generation sequencing reveals high prevalence of brca1 and brca2 variants of unknown significance in early-onset breast cancer in african american women | 2017 |
| 10 | Walsh T | genetic predisposition to breast cancer due to mutations other than brca1 and brca2 founder alleles among ashkenazi jewish women | 2017 |
| 11 | Yang XR | prevalence and spectrum of germline rare variants in brca1/2 and palb2 among breast cancer cases in sarawak, malaysia | 2017 |
| 12 | Brice√±o-Balc√°zar I | mutational spectrum in breast cancer associated brca1 and brca2 genes in colombia | 2017 |
| 13 | Fang M | characterization of mutations in brca1/2 and the relationship with clinic-pathological features of breast cancer in a hereditarily high-risk sample of chinese population | 2018 |
| 14 | Liang Y | prevalence and spectrum of brca1/2 germline mutations in women with breast cancer in china based on next-generation sequencing | 2018 |
| 15 | Abdel-Razeq H | germline brca1/brca2 mutations among high risk breast cancer patients in jordan | 2018 |
| 16 | Shah ND | mutation analysis of brca1/2 mutations with special reference to polymorphic snps in indian breast cancer patients | 2018 |
| 17 | Abulkhair O | prevalence of brca1 and brca2 mutations among high-risk saudi patients with breast cancer | 2018 |
| 18 | Wang T | mutational analysis of brca1 and brca2 in northwest chinese breast cancer patients | 2019 |
| 19 | Al Hannan F | characterization of brca1 and brca2 genetic variants in a cohort of bahraini breast cancer patients using next-generation sequencing | 2019 |
| 20 | Khalili-Tanha G | mutations analysis of brca1 gene in patients with breast cancer in south khorasan province, east iran | 2019 |
| 21 | Shen M | brca1/2 mutation spectrum in chinese early-onset breast cancer | 2019 |
| 22 | Geredeli C | germline mutations in brca1 and brca2 in breast cancer patients with high genetic risk in the turkish population | 2019 |
| 23 | Cort√©s C | mutational analysis of brca1 and brca2 genes in women with familial breast cancer from different regions of colombia | 2019 |
| 24 | Wu Y | profiling of the germline mutation brca1: p.ile1845fs in a large cohort of han chinese breast cancer | 2019 |
| 25 | Mahfoudh W | contribution of brca1 5382insc mutation in triple negative breast cancer in tunisia | 2019 |
| 26 | Millan Catalan O | a multi-center study of brca1 and brca2 germline mutations in mexican-mestizo breast cancer families reveals mutations unreported in latin american population | 2019 |
| 27 | Nishat L | identification of mutation in exon11 of brca1 gene in bangladeshi patients with breast cancer | 2019 |
| 28 | Behl S | founder brca1/brca2/palb2 pathogenic variants in french-canadian breast cancer cases and controls | 2020 |
| 29 | Hur JY | clinical characteristics of korean breast cancer patients who carry pathogenic germline mutations in both brca1 and brca2: a single-center experience | 2020 |
| 30 | Bakkach J | contribution of brca1 and brca2 germline mutations to early onset breast cancer: a series from north of morocco | 2020 |
| 31 | Abu-Helalah M | brca1 and brca2 genes mutations among high risk breast cancer patients in jordan | 2020 |
| 32 | Abdel-Razeq H | prevalence and predictors of germline brca1 and brca2 mutations among young patients with breast cancer in jordan | 2021 |
| 33 | Solano AR | study of the genetic variants in brca1/2 and non-brca genes in a population-based cohort of 2155 breast/ovary cancer patients, including 443 triple-negative breast cancer patients, in argentina | 2021 |
| 34 | Szczerba E | brca1/2 mutation detection in the tumor tissue from selected polish patients with breast cancer using next generation sequencing | 2021 |
| 35 | Bang YJ | clinicopathological characterization of double heterozygosity for brca1 and brca2 variants in korean breast cancer patients | 2022 |
| 36 | Stella S | mutational analysis of brca1 and brca2 genes in breast cancer patients from eastern sicily | 2022 |
| 37 | Brahim SM | screening of brca1/2 variants in mauritanian breast cancer patients | 2022 |
| 38 | Rweyemamu LP | breast cancer in east africa: prevalence and spectrum of germline snv/indel and cnvs in brca1 and brca2 genes among breast cancer patients in tanzania | 2023 |
| 39 | Melki R | increased prevalence of the founder brca1 c.5309g>t and recurrent brca2 c. 1310_1313delAGA mutations in breast cancer families from north eastern region of morocco: evidence of geographical specificity and high relevance for genetic counseling | 2023 |
| 40 | Rioki JN | brca1 and brca2 mutations and their clinical relevance in selected women diagnosed with triple-negative breast cancer in kenya: a descriptive cross-sectional study | 2023 |
| 41 | Zhang Y | brca1 and brca2 germline mutations in chinese hakka breast cancer patients | 2024 |
| 42 | Yu S | breast cancer risk associated with brca1 and brca2 pathogenic variants in the eastern chinese population | 2024 |
| 43 | Hassan AN | breast cancer high-penetrance genes brca1 and brca2 mutations using next-generation sequencing among iraqi kurdish women | 2024 |
| 44 | Al Amri WS | brca1/2 mutations and outcomes among middle eastern patients with early-onset breast cancer in oman | 2024 |
| 45 | Rojas LXR | germinal mutations among patients with breast cancer in colombia: is brca3 coming? | 2025 |
