# Supplementary material for: Global prevalence and ethnic variation of pathogenic BRCA1/2 variants in breast cancer: a systematic review and meta-analysis
Source: J Transl Med. 2026 Mar 12;24:555. doi: 10.1186/s12967-026-07997-3 (PMC13097826; doi:10.1186/s12967-026-07997-3)
Supplement: Supplementary file 4 — Supplementary Material 4 [file 12967_2026_7997_MOESM4_ESM.docx]

**Supplementary Table S2. List of BRCA1 Variants across different ethnicities**

| **Variant** | **Exon*** | **Protein Change** | **Detection Method** | **dbSNP150** | **Mutation Type** | **Variant impact** | **Cases tested** | **Carrier number** | **Reference # in Manuscript** | **Ethnicity/Population** | **Year of Study** |
| --- | --- | --- | --- | --- | --- | --- | --- | --- | --- | --- | --- |
| c.181T>G | 5 | p.Cys61Gly | Sanger | rs80357474 | Missense | Pathogenic | 200 | 2 | Dodova RI et al. (16) | Bulgarian | 2015 |
| c.5030_5033delCTAA | 17 | p.Thr1677_Asn1678delinsIlefs | Sanger | rs80357865 | Frameshift | Pathogenic | 200 | 1 |  | Bulgarian | 2015 |
| c.5263_5264insC | 20 | p.Ser1755delinsSerProfs | Sanger | rs80357906 | Frameshift | Pathogenic | 200 | 22 |  | Bulgarian | 2015 |
| c.4603G>T | 15 | p.Glu1535Ter | Sanger | rs80356898 | Nonsense | Pathogenic | 200 | 1 |  | Bulgarian | 2015 |
| c.464delA | 8 | p.Gln155fs | Sanger | - | Frameshift | Pathogenic | 200 | 1 |  | Bulgarian | 2015 |
| c.5397_5403delCCCTTGG | 22 | p.Thr1799delins | Sanger | - | Frameshift | Pathogenic | 200 | 1 |  | Bulgarian | 2015 |
|  |  |  |  |  |  |  |  |  |  |  |  |
| c.181T>G | 4 | p.Cys61Gly | NGS | - | Missense | Pathogenic | 108 | 1 | Francies FZ et al.(17) | White | 2015 |
| c.212G>A | 4 | p.Arg71Lys | NGS | - | Missense | Pathogenic | 108 | 1 |  | Black | 2015 |
| c.3593T>A | 10 | p.Leu1198* | NGS | - | Nonsense | Pathogenic | 108 | 1 |  | Indian | 2015 |
| c.1155G>A | 10 | p.Trp385* | NGS | - | Nonsense | Pathogenic | 108 | 1 |  | Black | 2015 |
| c.1953_1954insA | 10 | p.Lys652fs | NGS | - | Frameshift | Pathogenic | 108 | 1 |  | Black | 2015 |
|  |  |  |  |  |  |  |  |  |  |  |  |
| c.34C>T | 2 | p.Gln12* | Sanger / NGS | - | Nonsense | Pathogenic | 250 | 1 | El Saghir NS et al. (18) | Lebanese | 2015 |
| c.131G>T | 3 | p.Cys44Phe | Sanger / NGS | - | Missense | Pathogenic | 250 | 2 |  | Lebanese | 2015 |
| c.485_486delTG | 8 | p.Val162Glufs*19 | Sanger / NGS | - | Frameshift | Pathogenic | 250 | 1 |  | Lebanese | 2015 |
| c.2158G>T | 11 | p.Glu720* | Sanger / NGS | - | Nonsense | Pathogenic | 250 | 1 |  | Lebanese | 2015 |
| c.2410_2411del | 11 | p.Gln804Valfs*5 | Sanger / NGS | - | Frameshift | Pathogenic | 250 | 1 |  | Lebanese | 2015 |
| c.5030_5033del | 17 | p.Thr1677Ilefs*2 | Sanger / NGS | - | Frameshift | Pathogenic | 250 | 1 |  | Lebanese | 2015 |
|  |  |  |  |  |  |  |  |  |  |  |  |
| 185delAG | 2 | p.Glu23Valfs*17 | PCR + Sanger | - | Frameshift | Pathogenic | 190 | 3 | Villarreal-Garza C et al. (19) | Mexican | 2015 |
| 943ins10 | 11 | p.Asp265Valfs*22 | PCR + Sanger | - | Insertion | Pathogenic | 190 | 5 |  | Mexican | 2015 |
| c.2804_2807del | 11 | p.Gly935Glyfs*? | PCR + Sanger | - | Frameshift | Pathogenic | 190 | 4 |  | Mexican | 2015 |
| c.213C>G | 5 | p.Arg71Gly | PCR + Sanger | - | Missense | Pathogenic | 190 | 4 |  | Mexican | 2015 |
| c.3598C>T | 16 | p.Gln1200* | PCR + Sanger | - | Nonsense | Pathogenic | 190 | 1 |  | Mexican | 2015 |
| c.4327C>T | 13 | p.Arg1443* | PCR + Sanger | - | Nonsense | Pathogenic | 190 | 4 |  | Mexican | 2015 |
| c.5123C>A | 18 | p.Ala1708Glu | PCR + Sanger | - | Missense | Pathogenic | 190 | 1 |  | Mexican | 2015 |
| exon9-12del | 11 | deletion of exons 9 to 12 | PCR + Sanger | - | Large rearrangement | Pathogenic | 190 | 18 |  | Mexican | 2015 |
|  |  |  |  |  |  |  |  |  |  |  |  |
| c.3G>T | 2 | c.3G>T | MLPA |  | M |  | 369 | 1 | Pal T et al. (20) | Black Women (Florida) | 2015 |
| c.182G>A | 5 | c.182G>A | MLPA |  | M |  | 369 | 1 |  | Black Women (Florida) | 2015 |
| c.213-11T>G | 6 | c.213-11T>G | MLPA |  | IVS |  | 369 | 1 |  | Black Women (Florida) | 2015 |
| delExon8 | 8 | delExon8 | MLPA |  | LR |  | 369 | 1 |  | Black Women (Florida) | 2015 |
| c.824_825insAGCCATGTGG | 11 | c.824_825insAGCCATGTGG | MLPA |  | F |  | 369 | 2 |  | Black Women (Florida) | 2015 |
| c.2071delA | 11 | c.2071delA | MLPA |  | F |  | 369 | 1 |  | Black Women (Florida) | 2015 |
| c.3016delC | 11 | c.3016delC | MLPA |  | F |  | 369 | 1 |  | Black Women (Florida) | 2015 |
| c.3358_3359delGT | 11 | c.3358_3359delGT | MLPA |  | F |  | 369 | 1 |  | Black Women (Florida) | 2015 |
| c.3481_3491delGAAGATACTAG | 11 | c.3481_3491delGAAGATACTAG | MLPA |  | F |  | 369 | 1 |  | Black Women (Florida) | 2015 |
| c.4357+1G>A | 13 | c.4357+1G>A | MLPA |  | IVS |  | 369 | 5 |  | Black Women (Florida) | 2015 |
| dupExon13 | 13 | dupExon13 | MLPA |  | LR |  | 369 | 1 |  | Black Women (Florida) | 2015 |
| c.4484G>T | 14 | c.4484G>T | MLPA |  | M |  | 369 | 1 |  | Black Women (Florida) | 2015 |
| c.4986+6T>C | 16 | c.4986+6T>C | MLPA |  | IVS |  | 369 | 4 |  | Black Women (Florida) | 2015 |
| c.5152+1G>A | 18 | c.5152+1G>A | MLPA |  | IVS |  | 369 | 1 |  | Black Women (Florida) | 2015 |
| c.5177_5180delGAAA | 19 | c.5177_5180delGAAA | MLPA |  | F |  | 369 | 3 |  | Black Women (Florida) | 2015 |
| c.5251C>T | 20 | c.5251C>T | MLPA |  | N |  | 369 | 3 |  | Black Women (Florida) | 2015 |
| c.5324T>G | 21 | c.5324T>G | MLPA |  | M |  | 369 | 3 |  | Black Women (Florida) | 2015 |
| c.5387C>A | 22 | c.5387C>A | MLPA |  | N |  | 369 | 2 |  | Black Women (Florida) | 2015 |
| c.5467+1G>A | 23 | c.5467+1G>A | MLPA |  | IVS |  | 369 | 1 |  | Black Women (Florida) | 2015 |
|  |  |  |  |  |  |  |  |  |  |  |  |
| c.5266dupC | - | p.Gln1756Profs*74 | SYBR Green-based real-time allele-specific PCR | - | Frameshift | Pathogenic | 67 | 3 | Abdikhakimov A et al.(21) | Uzbek | 2016 |
|  |  |  |  |  |  |  |  |  |  |  |  |
| c.1140dupG | 11 | p.Lys381Glufs | Capture/Sanger Sequencing | - | Frameshift | Pathogenic | 818 | 5 | Bu R et al. (22) | Middle Eastern (Saudi Arabia) | 2016 |
| c.4065_4068delTCAA | 11 | p.Asn1355Lysfs | Capture/Sanger Sequencing | - | Frameshift | Pathogenic | 818 | 2 |  | Middle Eastern (Saudi Arabia) | 2016 |
| c.4136_4137delCT | 12 | p.Ser1379X | Capture/Sanger Sequencing | - | Frameshift | Pathogenic | 818 | 4 |  | Middle Eastern (Saudi Arabia) | 2016 |
| c.4524G>A | 15 | p.Trp1508X | Capture/Sanger Sequencing | - | Nonsense | Pathogenic | 818 | 1 |  | Middle Eastern (Saudi Arabia) | 2016 |
| IVS18+1G>C | 18 | IVS18+1G>C | Capture/Sanger Sequencing | - | Splicing | Pathogenic | 818 | 2 |  | Middle Eastern (Saudi Arabia) | 2016 |
| c.5251C>T | 20 | p.Arg1751X | Capture/Sanger Sequencing | - | Nonsense | Pathogenic | 818 | 5 |  | Middle Eastern (Saudi Arabia) | 2016 |
| c.5530delC | 24 | p.Leu1844SerfsX11 | Capture/Sanger Sequencing | - | Frameshift | Pathogenic | 818 | 4 |  | Middle Eastern (Saudi Arabia) | 2016 |
|  |  |  |  |  |  |  |  |  |  |  |  |
| c.154C>T | 5 | p.Leu52Phe | Direct sequencing | rs80357084 | Missense | Unclassified | 328 | 3 | Yoon KA et al. (23) | Korean | 2017 |
| c.547+14delG | IVS8 | c.547+14delG | Direct sequencing | rs273902771 | Splice region | Unclassified | 328 | 2 |  | Korean | 2017 |
| c.2566T>C | 11 | p.Tyr856His | Direct sequencing | rs80356892 | Missense | Unclassified | 328 | 19 |  | Korean | 2017 |
| c.3448C>T | 11 | p.Pro1150Ser | Direct sequencing | rs80357272 | Missense | Unclassified | 328 | 5 |  | Korean | 2017 |
| c.4883T>C | 16 | p.Met1628Thr | Direct sequencing | rs4986854 | Missense | Unclassified | 328 | 8 |  | Korean | 2017 |
| c.5339T>C | 22 | p.Leu1780Pro | Direct sequencing | rs80357474 | Missense | Potentially Pathogenic | 328 | 4 |  | Korean | 2017 |
|  |  |  |  |  |  |  |  |  |  |  |  |
| Asp232Asn | - | - | NGS | rs55975699 | Missense | VUS | 31 | 1 | Ricks-Santi L et al. (24) | African American | 2017 |
| Asn319Ser | - | - | NGS | rs397507258 | Missense | VUS | 31 | 1 |  | African American | 2017 |
| c.441+52delC | - | c.441+52delC | NGS | rs373202012 | Intron | Not found in ClinVar | 31 | 2 |  | African American | 2017 |
| c.441+51T>C | - | c.441+51T>C | NGS | rs578250989 | Intron | Not found in ClinVar | 31 | 16 |  | African American | 2017 |
| c.441+54A>G | - | c.441+54A>G | NGS | - | Intron | VUS | 31 | 1 |  | African American | 2017 |
|  |  |  |  |  |  |  |  |  |  |  |  |
| c.3607C>T | - | p.R1203X | Sequencing | - | Nonsense | Pathogenic | 3 | 1 | Walsh T et al. (25) | Ashkenazi Jewish | 2003 |
| c.4480G>T | - | p.E1494X | Sequencing | - | Nonsense | Pathogenic | 3 | 1 |  | Ashkenazi Jewish | 2003 |
| c.4986(+6)T>C | - | p.1662X | Sequencing | - | Splice site | Pathogenic | 3 | 1 |  | Ashkenazi Jewish | 2003 |
|  |  |  |  |  |  |  |  |  |  |  |  |
| c.5548delG |  | c.5548delG | Targeted Sequencing |  | frameshift | pathogenic | 467 | 1 | Yang XR et al. (26) | Malaysian (Sarawak) | 2017 |
| c.5335delC |  | c.5335delC | Targeted Sequencing | rs80357590 | frameshift | pathogenic | 467 | 2 |  | Malaysian (Sarawak) | 2017 |
| R1203X |  | c.3607C>T | Targeted Sequencing | rs62625308 | nonsense | pathogenic | 467 | 1 |  | Malaysian (Sarawak) | 2017 |
| c.3228_3229delAG |  | c.3228_3229delAG | Targeted Sequencing | rs80357635 | frameshift | pathogenic | 467 | 1 |  | Malaysian (Sarawak) | 2017 |
| c.3214delC |  | c.3214delC | Targeted Sequencing | rs80357923 | frameshift | pathogenic | 467 | 2 |  | Malaysian (Sarawak) | 2017 |
| S689X |  | S689X | Targeted Sequencing |  | nonsense | pathogenic | 467 | 1 |  | Malaysian (Sarawak) | 2017 |
| c.1140dupG |  | c.1140dupG | Targeted Sequencing | rs876659327 | frameshift | pathogenic | 467 | 1 |  | Malaysian (Sarawak) | 2017 |
| c.594+1G>A | - | c.594+1G>A | Targeted Sequencing | - | Splice site | Pathogenic | 467 | 1 |  | Malaysian (Sarawak) | 2017 |
| c.211C>G | - | p.R71G | Targeted Sequencing | rs80357382 | Missense | Pathogenic | 467 | 1 |  | Malaysian (Sarawak) | 2017 |
| c.182G>A | - | p.C61Y | Targeted Sequencing | rs80357093 | Missense | Pathogenic | 467 | 1 |  | Malaysian (Sarawak) | 2017 |
| c.110C>A | - | p.T37K | Targeted Sequencing | rs80356880 | Missense | Pathogenic | 467 | 1 |  | Malaysian (Sarawak) | 2017 |
|  |  |  |  |  |  |  |  |  |  |  |  |
| 3450delCAAG | - | 3450delCAAG | Sequencing | - | Frameshift | Pathogenic | 853 | 13 | Briceño-Balcázar I et al. (27) | Colombian | 2014 |
| A1708E | - | A1708E | Sequencing | - | Missense | Pathogenic | 853 | 27 |  | Colombian | 2014 |
| G3031A | - | G3031A | Sequencing | - | Unknown | NR | 853 | 1 |  | Colombian | 2014 |
| T3014C | - | T3014C | Sequencing | - | Unknown | NR | 853 | 1 |  | Colombian | 2014 |
| C5214T | - | C5214T | Sequencing | - | Unknown | Pathogenic | 853 | 1 |  | Colombian | 2014 |
| 1163delTG | - | 1163delTG | Sequencing | - | Frameshift | NR | 853 | 1 |  | Colombian | 2014 |
| C5141T | - | C5141T | Sequencing | - | Unknown | NR | 853 | 3 |  | Colombian | 2014 |
| 1793delA | - | 1793delA | Sequencing | - | Frameshift | Pathogenic | 853 | 4 |  | Colombian | 2014 |
| 5221delTG | - | 5221delTG | Sequencing | - | Frameshift | Pathogenic | 853 | 1 |  | Colombian | 2014 |
| 5221delT | - | 5221delT | Sequencing | - | Frameshift | NR | 853 | 1 |  | Colombian | 2014 |
| 5637delG | - | 5637delG | Sequencing | - | Frameshift | NR | 853 | 1 |  | Colombian | 2014 |
| C39R | - | C39R (234T>C) | Sequencing | - | Missense | NR | 853 | 2 |  | Colombian | 2014 |
| 4642G>A | - | W1508X | Sequencing | - | Nonsense | Pathogenic | 853 | 1 |  | Colombian | 2014 |
| 5154delTTTTC | - | 5154delTTTTC | Sequencing | - | Frameshift | NR | 853 | 1 |  | Colombian | 2014 |
| 2277G>T | - | E720X | Sequencing | - | Nonsense | NR | 853 | 1 |  | Colombian | 2014 |
| N1742S | - | N1742S | Sequencing | - | Missense | Uncertain | 853 | 1 |  | Colombian | 2014 |
| 2881delGACA | - | 2881delGACA | Sequencing | - | Frameshift | NR | 853 | 1 |  | Colombian | 2014 |
| 1499insA | - | 1499insA | Sequencing | - | Insertion | Pathogenic | 853 | 1 |  | Colombian | 2014 |
| V1145F | - | V1145F | Sequencing | - | Missense | NR | 853 | 2 |  | Colombian | 2014 |
| 2031delG | - | 2031delG | Sequencing | - | Frameshift | Pathogenic | 853 | 1 |  | Colombian | 2014 |
| K168E | - | K168E | Sequencing | - | Missense | NR | 853 | 1 |  | Colombian | 2014 |
| 5356delT | - | 5356delT | Sequencing | - | Frameshift | NR | 853 | 1 |  | Colombian | 2014 |
| 5622C>T | - | R1835X | Sequencing | - | Nonsense | Pathogenic | 853 | 1 |  | Colombian | 2014 |
| 5255G>A | - | W1712X | Sequencing | - | Nonsense | Pathogenic | 853 | 1 |  | Colombian | 2014 |
|  |  |  |  |  |  |  |  |  |  |  |  |
| c.3442delG | - | p.E1148fs | NGS | - | Frameshift | Pathogenic | 71 | 2 | Fang M et al.(28) | Chinese | 2017 |
| c.485_486delA | - | p.V162fs | NGS | - | Frameshift | Pathogenic | 71 | 1 |  | Chinese | 2017 |
| c.212G>A | - | p.R71K | NGS | - | Missense | Pathogenic | 71 | 1 |  | Chinese | 2017 |
| c.4676-1G>T | - | E1559_Splice | NGS | - | Splice site | Pathogenic | 71 | 1 |  | Chinese | 2017 |
| c.5278-1G>C | - | I1760_Splice | NGS | - | Splice site | Pathogenic | 71 | 1 |  | Chinese | 2017 |
| c.3626T>G | - | p.L1209X | NGS | - | Nonsense | Pathogenic | 71 | 1 |  | Chinese | 2017 |
|  |  |  |  |  |  |  |  |  |  |  |  |
| c.1504_1508delTTAAA | 11 | p.Leu502Ala fs | NGS + Sanger | - | Frameshift | Pathogenic | 595 | 1 | Liang Y et al. (29) | Chinese | 2018 |
| c.3333_3333delA | 11 | p.Glu1112fs | NGS + Sanger | - | Frameshift | Pathogenic | 595 | 1 |  | Chinese | 2018 |
| c.981_982delAT | 11 | p.Cys328fs | NGS + Sanger | - | Frameshift | Pathogenic | 595 | 1 |  | Chinese | 2018 |
| c.1299_1300insC | 11 | p.Ser434fs | NGS + Sanger | - | Frameshift | Pathogenic | 595 | 1 |  | Chinese | 2018 |
| c.1934_1934delC | 11 | p.Ser645fs | NGS + Sanger | - | Frameshift | Pathogenic | 595 | 1 |  | Chinese | 2018 |
| c.3214_3214delC | 11 | p.Leu1072fs | NGS + Sanger | - | Frameshift | Pathogenic | 595 | 1 |  | Chinese | 2018 |
| c.5510G>A | 24 | p.Trp1837Ter | NGS + Sanger | - | Nonsense | Pathogenic | 595 | 1 |  | Chinese | 2018 |
| c.1961_1961delA | 11 | p.Lys654fs | NGS + Sanger | - | Frameshift | Pathogenic | 595 | 1 |  | Chinese | 2018 |
| c.3352C>T | 11 | p.Gln1118Ter | NGS + Sanger | - | Nonsense | Pathogenic | 595 | 1 |  | Chinese | 2018 |
| c.376C>T | 7 | p.Gln126Ter | NGS + Sanger | - | Nonsense | Pathogenic | 595 | 1 |  | Chinese | 2018 |
| c.5353C>T | 22 | p.Gln1785Ter | NGS + Sanger | - | Nonsense | Pathogenic | 595 | 1 |  | Chinese | 2018 |
| c.3472G>T | 11 | p.Glu1158Ter | NGS + Sanger | - | Nonsense | Pathogenic | 595 | 1 |  | Chinese | 2018 |
| c.1012A>T | 11 | p.Lys338Ter | NGS + Sanger | - | Nonsense | Pathogenic | 595 | 1 |  | Chinese | 2018 |
| c.4222C>T | 13 | p.Gln1408Ter | NGS + Sanger | - | Nonsense | Pathogenic | 595 | 1 |  | Chinese | 2018 |
| c.1439_1440insA | 11 | p.Asn480fs | NGS + Sanger | - | Frameshift | Pathogenic | 595 | 1 |  | Chinese | 2018 |
| c.283_286delCTTG | 6 | p.Leu95fs | NGS + Sanger | - | Frameshift | Pathogenic | 595 | 1 |  | Chinese | 2018 |
| c.5521_5521delA | 24 | p.Ser1841fs | NGS + Sanger | - | Frameshift | Pathogenic | 595 | 1 |  | Chinese | 2018 |
|  |  |  |  |  |  |  |  |  |  |  |  |
| 3450del4 | - | 3450del4 / Stop 1115 | NGS/Sanger | - | Frameshift | Deleterious | 100 | 1 | Abdel-Razeq H et al. (30) | Jordanian | 2018 |
| 3954delG | - | 3954delG / Stop 1306 | NGS/Sanger | - | Frameshift | Deleterious | 100 | 2 |  | Jordanian | 2018 |
| 3555del4 | - | 3555del4 / Stop 1153 | NGS/Sanger | - | Frameshift | Deleterious | 100 | 1 |  | Jordanian | 2018 |
| c.4236G>T | - | E1373X | NGS/Sanger | - | Nonsense | Deleterious | 100 | 1 |  | Jordanian | 2018 |
| IVS17+3A>G | - | IVS17+3A>G | NGS/Sanger | - | Splice | Deleterious | 100 | 1 |  | Jordanian | 2018 |
| 5149del4 | - | 5149del4 / Stop 1678 | NGS/Sanger | - | Frameshift | Deleterious | 100 | 1 |  | Jordanian | 2018 |
| c.4553G>C | - | E1478D | NGS/Sanger | - | Missense | VUS | 100 | 1 |  | Jordanian | 2018 |
| c.1452G>C | - | E445Q | NGS/Sanger | - | Missense | FP | 100 | 1 |  | Jordanian | 2018 |
|  |  |  |  |  |  |  |  |  |  |  |  |
| c.4136_4137delCT | - | p.Ser1379* | NGS/Sanger | - | Frameshift | Pathogenic | 310 | 5 | Abulkhair O et al. (32) | Saudi Arabian | 2018 |
| c.4524G>A | - | p.Trp1508Ter* | NGS/Sanger | - | Nonsense | Pathogenic | 310 | 5 |  | Saudi Arabian | 2018 |
| c.5152+1G>C | - | IVS18+1G>T | NGS/Sanger | - | Splice | Pathogenic | 310 | 3 |  | Saudi Arabian | 2018 |
| c.5251C>T | - | p.Arg1751* | NGS/Sanger | - | Nonsense | Pathogenic | 310 | 3 |  | Saudi Arabian | 2018 |
| c.5530delC | - | p.Leu1844Serfs | NGS/Sanger | - | Frameshift | Pathogenic | 310 | 3 |  | Saudi Arabian | 2018 |
| c.5512dup | - | p.Val1838Glyfs*42 | NGS/Sanger | - | Frameshift | Pathogenic | 310 | 2 |  | Saudi Arabian | 2018 |
| 1326_1327insG | - | - | NGS/Sanger | - | Insertion | Pathogenic | 310 | 1 |  | Saudi Arabian | 2018 |
| c.1066C>T | - | - | NGS/Sanger | - | Nonsense | Pathogenic | 310 | 1 |  | Saudi Arabian | 2018 |
| c.1140dup | - | - | NGS/Sanger | - | Duplication | Pathogenic | 310 | 1 |  | Saudi Arabian | 2018 |
| c.124delA | - | p.Ile42Tyrfs* | NGS/Sanger | - | Frameshift | Pathogenic | 310 | 1 |  | Saudi Arabian | 2018 |
| c.1953delGAAA | - | p.Lys653Serfs*47 | NGS/Sanger | - | Frameshift | Pathogenic | 310 | 1 |  | Saudi Arabian | 2018 |
| c.1961dup | - | p.Tyr655Valfs*18 | NGS/Sanger | - | Duplication | Pathogenic | 310 | 1 |  | Saudi Arabian | 2018 |
| c.4065_4068delTCAA | - | p.Asn1355_Gln1356?fs | NGS/Sanger | - | Frameshift | Pathogenic | 310 | 1 |  | Saudi Arabian | 2018 |
| c.4524A>T | - | p.Gly1508Gly | NGS/Sanger | - | Synonymous | Likely benign | 310 | 1 |  | Saudi Arabian | 2018 |
| c.4609C>T | - | p.Gln1537* | NGS/Sanger | - | Nonsense | Pathogenic | 310 | 1 |  | Saudi Arabian | 2018 |
| c.4676-2A>G | - | - | NGS/Sanger | - | Splice | Pathogenic | 310 | 1 |  | Saudi Arabian | 2018 |
| c.5030_5033del | - | p.Thr1677Ilefs*2 | NGS/Sanger | - | Frameshift | Pathogenic | 310 | 1 |  | Saudi Arabian | 2018 |
| c.5074+2T>A | - | - | NGS/Sanger | - | Splice | Pathogenic | 310 | 1 |  | Saudi Arabian | 2018 |
|  |  |  |  |  |  |  |  |  |  |  |  |
| c.4485-2A>C | 14 | A>C | NGS | - | Splice | Pathogenic | 82 | 1 | Wang T et al. (33) | Chinese | 2019 |
| c.5470-5477del | 23 | TGCCCAAT/- | NGS | - | Frameshift | Pathogenic | 82 | 1 |  | Chinese | 2019 |
| c.190T>C | ? | T>C | NGS | - | Missense | Pathogenic | 82 | 1 |  | Chinese | 2019 |
| c.4837A>G | 15 | A>G | NGS | - | Missense | Drug-sensitive | 82 | 4 |  | Chinese | 2019 |
| c.2612C>T | 10 | C>T | NGS | - | Missense | Drug-sensitive | 82 | 4 |  | Chinese | 2019 |
|  |  |  |  |  |  |  |  |  |  |  |  |
| c.4850C>A | 16 | p.Ser1617X | NGS |  | Nonsense | Deleterious | 25 | 1 | Al Hannan F et al. (34) | Bahraini | 2019 |
|  |  |  |  |  |  |  |  |  |  |  |  |
| c.3113A>G | 11 | p.Glu1038Gly | SSCP + Sequencing | rs16941 | Missense | Polymorphism | 40 | 13 | Khalili-Tanha G et al.(35) | Iranian (South Khorasan) | 2019 |
| c.3119G>A | 11 | p.Ser1040Asn | SSCP + Sequencing | rs4986852 | Missense | Polymorphism | 40 | 2 |  | Iranian (South Khorasan) | 2019 |
| c.3548A>G | 11 | pLys1183Arg | SSCP + Sequencing | rs16942 | Missense | Polymorphism | 40 | 26 |  | Iranian (South Khorasan) | 2019 |
| c.4308T>C | 13 | Ser1436Ser | SSCP + Sequencing | rs1060915 | Synonymous | Polymorphism | 88 | 28 |  | Iranian (South Khorasan) | 2019 |
| c.4837A>G | 16 | Ser1613Gly | SSCP + Sequencing | rs1799966 | Missense | Polymorphism | 88 | 50 |  | Iranian (South Khorasan) | 2019 |
|  |  |  |  |  |  |  |  |  |  |  |  |
|  |  |  |  |  |  |  |  |  |  |  |  |
| c.213-1G>A | 4 | c.213-1G>A | PGM & Miseq |  | Splice_acceptor_variant | Pathogenic | 54 | 1 | Shen M et al.(36) | Chinese | 2019 |
| c.988G>A | 10 | p.Asp330Asn | PGM & Miseq |  | Missense_variant | Uncertain | 54 | 1 |  | Chinese | 2019 |
| c.1036C>T | 10 | p.Pro346Ser | PGM & Miseq |  | Missense_variant | Uncertain | 54 | 1 |  | Chinese | 2019 |
| c.1299dupC | 10 | p.Ser434GlnfsTer2 | PGM & Miseq |  | Frameshift_variant | Pathogenic | 54 | 1 |  | Chinese | 2019 |
| c.2059C>T | 10 | p.Gln687Ter | PGM & Miseq |  | Stop_gained | Pathogenic | 54 | 1 |  | Chinese | 2019 |
| c.2566T>C | 10 | p.Tyr856His | PGM & Miseq |  | Missense_variant |  | 54 | 15 |  | Chinese | 2019 |
| c.2612C>T | 10 | p.Pro871Leu | PGM & Miseq |  | Missense_variant |  | 54 | 33 |  | Chinese | 2019 |
| c.2623C>T | 10 | p.Pro875Ser | PGM & Miseq |  | Missense_variant | Uncertain | 54 | 1 |  | Chinese | 2019 |
| c.3113A>G | 10 | p.Glu1038Gly | PGM & Miseq |  | Missense_variant |  | 54 | 36 |  | Chinese | 2019 |
| c.3548A>G | 10 | p.Lys1183Arg | PGM & Miseq |  | Missense_variant |  | 54 | 34 |  | Chinese | 2019 |
| c.4674A>G | 15 | (p.Leu1558=) | PGM & Miseq |  | Synonymous_variant | Uncertain | 54 | 1 |  | Chinese | 2019 |
| c.4837A>G | 16 | p.Ser1613Gly | PGM & Miseq |  | Missense_variant |  | 54 | 32 |  | Chinese | 2019 |
| c.446A>C | 7 | p.Glu149Ala | PGM & Miseq |  | Missense_variant |  | 54 | 1 |  | Chinese | 2019 |
| c.2398_2401delAAAT | 10 | p.Lys800ValfsTer2 | PGM & Miseq |  | Frameshift_variant | Pathogenic | 54 | 1 |  | Chinese | 2019 |
|  |  |  |  |  |  |  |  |  |  |  |  |
| c.3607C>T |  | p.R1203 | NGS |  | Nonsense |  | 99 | 2 | Geredeli C et al. (37) | Turkish | 2019 |
| c.4837A>G) |  | p.S1613G | NGS |  | Missense |  | 99 | 2 |  | Turkish | 2019 |
| c.5152+66g>A |  | IVS18 +66G>A | NGS |  | Splice site |  | 99 | 1 |  | Turkish | 2019 |
| (c1067A>G |  | p.Q356 | NGS |  | Missense |  | 99 | 1 |  | Turkish | 2019 |
| c.442-34C>T |  | IVS7 -34C>T | NGS |  |  |  | 99 | 1 |  | Turkish | 2019 |
| c.5444G>A |  | p.W1815 | NGS |  | Missense |  | 99 | 2 |  | Turkish | 2019 |
| c.3624dupA |  | p.L1209lfs 10 | NGS |  | Frameshift |  | 99 | 2 |  | Turkish | 2019 |
|  |  |  |  |  |  |  |  |  |  |  |  |
| c.412C>A |  | p.Leu138Ile | NGS |  | Missense | Non-synonymous | 201 | 1 | Cortés C et al. (38) | Columbian | 2021 |
| c.4186-22G>A |  | NA | NGS |  | Intronic | Intronic | 201 | 2 |  | Columbian | 2021 |
| c.2077G>A |  | p.Asp693Asn | NGS |  | Missense | Non-synonymous | 201 | 2 |  | Columbian | 2021 |
| c.2079G>A |  | p.Asp693Asp | NGS |  | Synonymous | Synonymous | 201 | 2 |  | Columbian | 2021 |
| c.2082C>T |  | p.Ser694Ser | NGS |  | Synonymous | Synonymous | 201 | 30 |  | Columbian | 2021 |
| c.2146T>A |  | p.Ser716Arg | NGS |  | Missense | Non-synonymous | 201 | 2 |  | Columbian | 2021 |
| c.2311T>C |  | p.Leu771Leu | NGS |  | Synonymous | Synonymous | 201 | 13 |  | Columbian | 2021 |
| c.2368A>G |  | p.Thr790Ala | NGS |  | Missense | Non-synonymous | 201 | 2 |  | Columbian | 2021 |
| c.2612C>T |  | p.Pro871Leu | NGS |  | Missense | Non-synonymous | 201 | 64 |  | Columbian | 2021 |
| c.2876G>A |  | p.Arg959Lys | NGS |  | Missense | Non-synonymous | 201 | 2 |  | Columbian | 2021 |
| c.3083G>A |  | p.Arg1028His | NGS |  | Missense | Non-synonymous | 201 | 3 |  | Columbian | 2021 |
| c.3113A>G |  | p.Glu1038Gly | NGS |  | Missense | Non-synonymous | 201 | 27 |  | Columbian | 2021 |
| c.3506A>C |  | p.Asp1169Ala | NGS |  | Missense | Non-synonymous | 201 | 1 |  | Columbian | 2021 |
| c.3548A>G |  | p.Lys1183Arg | NGS |  | Missense | Non-synonymous | 201 | 20 |  | Columbian | 2021 |
| c.3978T>G |  | p.His1326Gln | NGS |  | Missense | Non-synonymous | 201 | 1 |  | Columbian | 2021 |
| c.4033G>A |  | p.Glu1345Lys | NGS |  | Missense | Non-synonymous | 201 | 4 |  | Columbian | 2021 |
| c.4308T>C |  | p.Ser1436Ser | NGS |  | Synonymous | Synonymous | 201 | 8 |  | Columbian | 2021 |
| c.4837A>G |  | p.Ser1613Gly | NGS |  | Missense | Non-synonymous | 201 | 17 |  | Columbian | 2021 |
|  |  |  |  |  |  |  |  |  |  |  |  |
| c.5536_5539del | 23 | p.Ile1845fs | NGS + Sanger sequencing | Not listed | Frameshift | Pathogenic | 23481 | 94 | Wu Y et al. (39) | Han Chinese | 2020 |
|  |  |  |  |  |  |  |  |  |  |  |  |
| c.5266dupC | 20 | p.Gln1756fs | Sanger sequencing | Not listed | Frameshift | Pathogenic | 33 | 2 | Mahfoudh W et al.(40) | Tunisia | 2019 |
|  |  |  |  |  |  |  |  |  |  |  |  |
| c.66_67delAG |  | p.Leu22fs | NGS | rs80357783 | Frameshift | Pathogenic | 252 | 4 | Millan Catalan O et al.(41) | Latin American | 2022 |
| c.69_70insAG |  | p.Cys24fs | NGS | rs80357914 | Frameshift | Pathogenic | 252 | 1 |  | Latin American | 2022 |
| c.211A>G |  | p.Arg71Gly | NGS | rs80357382 | Missense | Pathogenic | 252 | 2 |  | Latin American | 2022 |
| c.798_799delTT |  | p.Ser267fs | NGS | rs80357724 | Frameshift | Pathogenic | 252 | 1 |  | Latin American | 2022 |
| c.1504_1508delTTAAA |  | p.Leu502fs | NGS | rs876659139 | Frameshift | Pathogenic | 252 | 1 |  | Latin American | 2022 |
| c.1960A>T |  | p.Lys654Ter | NGS | rs80357355 | Nonsense | Pathogenic | 252 | 2 |  | Latin American | 2022 |
| c.2806_2809delGATA |  | p.Asp936fs | NGS | rs80357832 | Frameshift | Pathogenic | 252 | 1 |  | Latin American | 2022 |
| c.3598C>T |  | p.Gln1200Ter | NGS | rs62625307 | Nonsense | Pathogenic | 252 | 3 |  | Latin American | 2022 |
| c.3759_3760delTA |  | p.Lys1254Glufs | NGS | rs80357520 | Frameshift | Pathogenic | 252 | 1 |  | Latin American | 2022 |
| c.3858_3861delTGAG |  | p.Ser1286fs | NGS | rs80357842 | Frameshift | Pathogenic | 252 | 1 |  | Latin American | 2022 |
| c.4065_4068delTCAA |  | p.Asn1355fs | NGS | rs80357508 | Frameshift | Pathogenic | 252 | 1 |  | Latin American | 2022 |
| c.4327C>T |  | p.Arg1443Ter | NGS | rs41293455 | Nonsense | Pathogenic | 252 | 1 |  | Latin American | 2022 |
| c.5095C>T |  | p.Arg1699Trp | NGS | rs55770810 | Missense | Pathogenic | 252 | 1 |  | Latin American | 2022 |
| c.5123C>A |  | p.Ala1708Glu | NGS | rs28897696 | Missense | Pathogenic | 252 | 3 |  | Latin American | 2022 |
| c.5263_5264insC |  | p.Ser1755fs | NGS | rs80357906 | Frameshift | Pathogenic | 252 | 1 |  | Latin American | 2022 |
| c.212+1G>A |  | Splicing mutation | NGS | rs80358042 | Splice | Pathogenic | 252 | 1 |  | Latin American | 2022 |
|  |  |  |  |  |  |  |  |  |  |  |  |
| g.852G>C | Exon11 | p.Gln284His | Sanger Sequencing |  | Substitution | Missense | 65 | 1 | Nishat L et al. (42) | Bangladeshi | 2019 |
| g.709G>A | Exon11 | p.Glu237Lys | Sanger Sequencing |  | Substitution | Missense | 65 | 1 |  | Bangladeshi | 2019 |
| g.711A>G | Exon11 | p.Glu237Glu | Sanger Sequencing |  | Substitution | Synonymous | 65 | 1 |  | Bangladeshi | 2019 |
|  |  |  |  |  |  |  |  |  |  |  |  |
| c.962G>A |  | p.(Trp321Ter) | PCR + Sanger |  |  |  | 522 | 0 | Behl S et al. (43) | French-Canadians | 2020 |
| c.1016dupA |  | p.(Val340GlyfsTer6) | PCR + Sanger |  |  |  | 555 | 0 |  | French-Canadians | 2020 |
| c.1961dupA |  | p.(Tyr655ValfsTer18) | PCR + Sanger |  |  |  | 555 | 0 |  | French-Canadians | 2020 |
| c.2125_2126insA |  | p.(Phe709TyrfsTer3) | PCR + Sanger |  |  |  | 555 | 1 |  | French-Canadians | 2020 |
| c.2834_2836delGTAinsC | | p.(Ser945TrfsTer6) | PCR + Sanger |  |  |  | 555 | 0 |  | French-Canadians | 2020 |
| c.3649_3650insA |  | p.(Ser1217TyrfsTer2) | PCR + Sanger |  |  |  | 553 | 0 |  | French-Canadians | 2020 |
| c.3756_3759delGTCT |  | p.(Ser1253ArgfsTer10) | PCR + Sanger |  |  |  | 555 | 0 |  | French-Canadians | 2020 |
| c.4327C>T |  | p.(Arg1443Ter) | PCR + Sanger |  |  |  | 552 | 3 |  | French-Canadians | 2020 |
| c.5102_5103delTG |  | p.(Leu1701GlnfsTer14) | PCR + Sanger |  |  |  | 555 | 0 |  | French-Canadians | 2020 |
| c.1054G>T |  | p.(Glu352Ter) | PCR + Sanger |  |  |  | 553 | 0 |  | French-Canadians | 2020 |
| c.5536C>T |  | p.(Gln1846Ter) | PCR + Sanger |  |  |  | 555 | 0 |  | French-Canadians | 2020 |
|  |  |  |  |  |  |  |  |  |  |  |  |
| c.390C>A |  | p.Tyr130Ter | NGS | rsID_not_found | Nonsense | High | 2720 | 1 | Hur JY et al. (44) | Korean | 2020 |
| c.922_924delinsT |  | p.Lys307_Ser308insTer | NGS | rsID_not_found | Frameshift | High | 2720 | 1 |  | Korean | 2020 |
| c.5496_5506delinsA |  | p.Val1835fs | NGS | rsID_not_found | Frameshift | High | 2720 | 1 |  | Korean | 2020 |
| c.5300_5303del |  | p.Thr1767fs | NGS | rsID_not_found | Frameshift | High | 2720 | 1 |  | Korean | 2020 |
| c.5445G>A |  | p.Trp1815Ter | NGS | rsID_not_found | Nonsense | High | 2720 | 1 |  | Korean | 2020 |
| c.3627dupA |  | p.Glu1210Argfs | NGS | rsID_not_found | Frameshift | High | 2720 | 2 |  | Korean | 2020 |
| c.4933delA |  | p.Arg1645Glyfs | NGS | rsID_not_found | Frameshift | High | 2720 | 1 |  | Korean | 2020 |
| c.302-2A>C |  |  | NGS | rsID_not_found | Splice_acceptor | High | 2720 | 1 |  | Korean | 2020 |
| c.2405_2406delTG |  | p.Val802Glufs | NGS | rsID_not_found | Frameshift | High | 2720 | 1 |  | Korean | 2020 |
| c.5080G>T |  | p.Glu1694Ter | NGS | rsID_not_found | Nonsense | High | 2720 | 1 |  | Korean | 2020 |
|  |  |  |  |  |  |  |  |  |  |  |  |
| c.116G > A | 3 | NP_009225.1: p.Cys39Tyr | NGS |  | Missense | Likely Pathogenic | 33 | 1 | Bakkach J et al. (45) | Morrocon | 2020 |
| c.2125_ 2126insA | 11 | NP_009225.1: p.Phe709Tyrfs | NGS |  | Frameshift | Pathogenic | 33 | 1 |  | Morrocon | 2020 |
| c.798_ 799del | 11 | NP_009225.1: p.Ser267Lysfs | NGS |  | Frameshift | Pathogenic | 33 | 1 |  | Morrocon | 2020 |
|  |  |  |  |  |  |  | 33 | 1 |  |  |  |
| c.5186C>A | 18 | p.Ala1729Glu | NGS | rs28897696 | Missense | Pathogenic | 192 | 1 | Abu-Helalah M et al. (46) | Jordan | 2020 |
| c.5158C>T | 18 | p.Arg1720Trp | NGS | rs55770810 | Missense | Pathogenic | 192 | 1 |  | Jordan | 2020 |
| c.4065_4068delTCAA | 10 | p.Asn1355Lysfs*1 | NGS | rs80357508 | Frameshift | Pathogenic | 192 | 1 |  | Jordan | 2020 |
| c.121C>T | 3 | p.His41Tyr |  | n/a | Missense | n/a | 192 | 1 |  |  |  |
|  |  |  |  |  |  |  |  |  |  |  |  |
| Deletion (exons 1–2) | Exon 1–2 | Absent or disrupted protein product | NGS |  | Large deletion |  | 616 | 1 | Abdel-Razeq H et al. (30) | Jordan | 2020 |
| c.66dup | Exon 2 | p.Glu23Argfs | NGS |  | Duplication/frameshift |  | 616 | 1 |  | Jordan | 2020 |
| c.121C>T | Exon 3 | p.His41Tyr | NGS |  | Missense |  | 616 | 1 |  | Jordan | 2020 |
| c.3853del | Exon 11 | p.Ala1279Hisfs | NGS |  | Deletion/frameshift |  | 616 | 1 |  | Jordan | 2020 |
| c.346_3439del | Exon 11 | p.Cys1146LeufsTer | NGS |  | Deletion/frameshift |  | 616 | 1 |  | Jordan | 2020 |
| c.798_799del | Exon 11 | p.Ser267Lysfs | NGS |  | Deletion/frameshift |  | 616 | 1 |  | Jordan | 2020 |
| c.2761C>T | Exon 12 | p.Gln921Ter | NGS |  | Nonsense |  | 616 | 1 |  | Jordan | 2020 |
| c.1961del | Exon 11 | p.Lys654Serfs | NGS |  | Deletion/frameshift |  | 616 | 1 |  | Jordan | 2020 |
| c.809del | Exon 11 | p.His270Leufs | NGS |  | Deletion/frameshift |  | 616 | 1 |  | Jordan | 2020 |
| c.4065_4068del | Exon 11 | p.Asn1355Lysfs | NGS |  | Deletion/frameshift |  | 616 | 2 |  | Jordan | 2020 |
| c.4117G>T | Exon 12 | p.Glu1373Ter | NGS |  | Nonsense |  | 616 | 4 |  | Jordan | 2020 |
| c.4524G>A | Exon 15 | p.Trp1508Ter | NGS |  | Nonsense |  | 616 | 1 |  | Jordan | 2020 |
| c.5030_5033del | Exon 15 | p.Thr1677Ilefs | NGS |  | Deletion/frameshift |  | 616 | 1 |  | Jordan | 2020 |
| c.4117G>A | Exon 12 | p.Ala1708Glu | NGS |  | Missense |  | 616 | 1 |  | Jordan | 2020 |
| c.5095C>T | Exon 18 | p.Arg1699Trp | NGS |  | Missense |  | 616 | 1 |  | Jordan | 2020 |
| c.5161C>T | Exon 19 | p.Gln1721Ter | NGS |  | Nonsense |  | 616 | 2 |  | Jordan | 2020 |
| c.5074+3A>G / IVS17+3 | Intron 17 | Splice acceptor | NGS |  | Splice site |  | 616 | 3 |  | Jordan | 2020 |
|  |  |  |  |  |  |  |  |  |  |  |  |
| c.(-?_-232)_(80+1_81-1)del | 2_2i | Glu23Valfs*17 | NGS |  | Nonsense | Likely pathogenic/pathogenic | 443 | 1 | Solano AR et al. (47) | Argentina | 2021 |
| c.68_69del | 2 | Glu23Valfs*17 | NGS |  | Nonsense | Likely pathogenic/pathogenic | 443 | 12 |  | Argentina | 2021 |
| c.81-1G>A | 2i |  | NGS |  |  | Likely pathogenic/pathogenic | 443 | 1 |  | Argentina | 2021 |
| c.134+2T>C | 3i |  | NGS |  |  | Likely pathogenic/pathogenic | 443 | 1 |  | Argentina | 2021 |
| c.134+1_135-1_(1383_?)del | 3i_24_ |  | NGS |  | Missense | Likely pathogenic/pathogenic | 443 | 1 |  | Argentina | 2021 |
| c.140G>T | 5 | Cys47Phe | NGS |  |  | Likely pathogenic/pathogenic | 443 | 1 |  | Argentina | 2021 |
| c.1480C>T | 5 | Cys494Ter | NGS |  | Missense | Likely pathogenic/pathogenic | 443 | 1 |  | Argentina | 2021 |
| c.181T>G | 5 | Cys61Gly | NGS |  | Missense | Likely pathogenic/pathogenic | 443 | 2 |  | Argentina | 2021 |
| c.187_188del | 5 | Leu63Metfs*2 | NGS |  | Nonsense | Likely pathogenic/pathogenic | 443 | 1 |  | Argentina | 2021 |
| c.187_189delinsATA | 5 | Leu63Metfs*2 | NGS |  | Nonsense | Likely pathogenic/pathogenic | 443 | 1 |  | Argentina | 2021 |
| c.190_191ins19 | 5 | Cys64* | NGS |  | Nonsense | Likely pathogenic/pathogenic | 443 | 2 |  | Argentina | 2021 |
| c.191G>A | 5 | Cys64Tyr | NGS |  | Missense | Likely pathogenic/pathogenic | 443 | 1 |  | Argentina | 2021 |
| c.190T>C | 5 | Cys64Arg | NGS |  | Missense | Likely pathogenic/pathogenic | 443 | 1 |  | Argentina | 2021 |
| c.211A>G | 5 | Arg71Gly |  |  | Missense | Likely pathogenic/pathogenic | 443 | 8 |  | Argentina | 2021 |
| c.211del | 5 | Arg71Glyfs*17 | NGS |  | Missense | Likely pathogenic/pathogenic | 443 | 1 |  | Argentina | 2021 |
| c.213-11T>G | 5i |  | NGS |  | Nonsense | Likely pathogenic/pathogenic | 443 | 1 |  | Argentina | 2021 |
| c.427G>T | 7 | Glu143 | NGS |  | Missense | Likely pathogenic/pathogenic | 443 | 1 |  | Argentina | 2021 |
| c.470_471del | 8 | p.Ser157* | NGS |  | Deletion | Likely pathogenic/pathogenic | 443 | 1 |  | Argentina | 2021 |
| c.1039_1040del | 11 | p.Leu347Valfs*2 | NGS |  | Deletion | Likely pathogenic/pathogenic | 443 | 1 |  | Argentina | 2021 |
| c.1067del | 11 | p.Gln356Argfs*18 | NGS |  | Deletion | Likely pathogenic/pathogenic | 443 | 1 |  | Argentina | 2021 |
| c.1504_1507del | 11 | p.Leu502Serfs*29 | NGS |  | Deletion | Likely pathogenic/pathogenic | 443 | 1 |  | Argentina | 2021 |
| c.1687C>T | 11 | p.Gln563* | NGS |  | Substitution | Likely pathogenic/pathogenic | 443 | 1 |  | Argentina | 2021 |
| c.2296_2297del | 11 | p.Ser766* | NGS |  | Deletion | Likely pathogenic/pathogenic | 443 | 1 |  | Argentina | 2021 |
| c.3228_3229del | 11 | p.Gly1077Alafs*8 | NGS |  | Deletion | Likely pathogenic/pathogenic | 443 | 1 |  | Argentina | 2021 |
| c.3331_3334del | 11 | p.Gln1111Asnfs*5 | NGS |  | Deletion | Likely pathogenic/pathogenic | 443 | 1 |  | Argentina | 2021 |
| c.3627dup | 11 | p.Glu1210Argfs*9 | NGS |  | Duplication | Likely pathogenic/pathogenic | 443 | 1 |  | Argentina | 2021 |
| c.3858_3861del | 11 | p.Ser1286Argfs*20 | NGS |  | Deletion | Likely pathogenic/pathogenic | 443 | 1 |  | Argentina | 2021 |
| c.4042G>T | 11 | p.Gly1348* | NGS |  | Nonsense | Likely pathogenic/pathogenic | 443 | 1 |  | Argentina | 2021 |
| c.4065_4068del | 11 | p.Asn1355Lysfs*10 | NGS |  | Frameshift | Likely pathogenic/pathogenic | 443 | 1 |  | Argentina | 2021 |
| c.4183C>T | 11 | p.Gln1395* | NGS |  | Nonsense | Likely pathogenic/pathogenic | 443 | 1 |  | Argentina | 2021 |
| c.4201C>T | 13 | p.Gln1401* | NGS |  | Nonsense | Likely pathogenic/pathogenic | 443 | 1 |  | Argentina | 2021 |
| c.4327C>T | 13 | p.Arg1443* | NGS |  | Nonsense | Likely pathogenic/pathogenic | 443 | 1 |  | Argentina | 2021 |
| c.(4357+1_4358-1)_(4484+1_4485-1)del | 13i-14i |  | NGS |  | Splice site | Likely pathogenic/pathogenic | 443 | 1 |  | Argentina | 2021 |
| c.4392del | 14 | p.Ile1465* | NGS |  | Frameshift | Likely pathogenic/pathogenic | 443 | 1 |  | Argentina | 2021 |
| c.4484G>T | 14 | p.Arg1495Met | NGS |  | Missense | Likely pathogenic/pathogenic | 443 | 1 |  | Argentina | 2021 |
| c.4675+2T>A | 15i |  | NGS |  | Splice site | Likely pathogenic/pathogenic | 443 | 1 |  | Argentina | 2021 |
| c.(4675+1_4676-1)_(5074+1_5075-1)del | 15i-17i |  | NGS |  | Splice site | Likely pathogenic/pathogenic | 443 | 2 |  | Argentina | 2021 |
| c.4736_4739del | 16 | p.Pro1579Leufs*21 | NGS |  | Frameshift | Likely pathogenic/pathogenic | 443 | 1 |  | Argentina | 2021 |
| c.4964_4982del | 16 | p.Ser1655Tyrfs*16 | NGS |  | Frameshift | Likely pathogenic/pathogenic | 443 | 2 |  | Argentina | 2021 |
| c.4986+4A>C | 16i |  | NGS |  | Splice region | Likely pathogenic/pathogenic | 443 | 1 |  | Argentina | 2021 |
| c.5030_5033del | 17 | p.Thr1677Ilefs*2 | NGS |  | Frameshift | Likely pathogenic/pathogenic | 443 | 3 |  | Argentina | 2021 |
| c.5095C>T | 18 | p.Arg1699Trp | NGS |  | Missense | Likely pathogenic/pathogenic | 443 | 1 |  | Argentina | 2021 |
| c.5123C>A | 18 | p.Ala1708Glu | NGS |  | Missense | Likely pathogenic/pathogenic | 443 | 1 |  | Argentina | 2021 |
| c.(5193+1_5194-1)_(5227+1_5278-1)del | 19i-20i |  | NGS |  | Splice site | Likely pathogenic/pathogenic | 443 | 1 |  | Argentina | 2021 |
| c.5266dup | 20 | p.Gln1756Profs*74 | NGS |  | Frameshift | Likely pathogenic/pathogenic | 443 | 3 |  | Argentina | 2021 |
| c.5282T>C | 21 | p.Phe1761Ser | NGS |  | Missense | Likely pathogenic/pathogenic | 443 | 1 |  | Argentina | 2021 |
| c.5431C>T | 23 | p.Gln1811* | NGS |  | Nonsense | Likely pathogenic/pathogenic | 443 | 1 |  | Argentina | 2021 |
| c.5445G>A | 23 | p.Trp1815* | NGS |  | Nonsense | Likely pathogenic/pathogenic | 443 | 1 |  | Argentina | 2021 |
| c.(5467+1_5468-1)_(*1383_?)del | 23i_24_ |  | NGS |  | Large deletion | Likely pathogenic/pathogenic | 443 | 1 |  | Argentina | 2021 |
|  |  |  |  |  |  |  |  |  |  |  |  |
| c.1687C>T |  | p.Gln563Ter | NGS |  | Nonsense | Pathogenic | 75 | 1 | Szczerba E et al. (48) | Polish | 2021 |
| c.4752C>G |  | p.Tyr1584Ter | NGS |  | Nonsense | Pathogenic | 75 | 2 |  | Polish | 2021 |
| c.5186C>A |  | p.Ala1729Glu | NGS |  | Nonsense | Pathogenic | 75 | 1 |  | Polish | 2021 |
| c.5242A>T |  | p.Lys1748Ter | NGS |  | Nonsense | Pathogenic | 75 | 1 |  | Polish | 2021 |
|  |  |  |  |  |  |  |  |  |  |  |  |
| c.390C>A |  | p.Tyr130* | NGS |  | Nonsense |  | 4215 | 26 | Bang YJ et al. (49) | Korean | 2021 |
| c.5496_5506delinsA |  | p.Val1833Serfs*7 | NGS |  | Frameshift |  | 4215 | 23 |  | Korean | 2021 |
| c.3627dup |  | p.Glu1210Argfs*9 | NGS |  | Frameshift |  | 4215 | 19 |  | Korean | 2021 |
| c.5339T>C |  |  | NGS |  | Missense |  | 4215 | 16 |  | Korean | 2021 |
| c.922_924delinsT |  | p.Ser308* | NGS |  | Nonsense |  | 4215 | 15 |  | Korean | 2021 |
| c.5445G>A |  |  | NGS |  | Nonsense |  | 4215 | 14 |  | Korean | 2021 |
| c.5080G>T |  |  | NGS |  | Missense |  | 4215 | 7 |  | Korean | 2021 |
|  |  |  |  |  |  |  |  |  |  |  |  |
| c.66_67insA |  | p.(Glu23Argfs) | NGS |  | Duplication | Pathogenic | 376 | 1 | Stella S et al. (50) | Sicily | 2022 |
| c.117_118delTG |  | p.(Cys39Ter) | NGS |  | Deletion | Pathogenic | 376 | 1 |  | Sicily | 2022 |
| c.181T>G |  | p.(Cys61Gly) | NGS |  | SNV | Pathogenic | 376 | 1 |  | Sicily | 2022 |
| c.514del |  | p.(Gln172Asnfs) | NGS |  | Deletion | Pathogenic | 376 | 1 |  | Sicily | 2022 |
| c.1204G>T |  | p.(Glu402Ter) | NGS |  | SNV | Pathogenic | 376 | 1 |  | Sicily | 2022 |
| c.1360_1361delAG |  | p.(Ser454Ter) | NGS |  | Deletion | Pathogenic | 376 | 1 |  | Sicily | 2022 |
| c.2350_2351delTC |  | p.(Ser784Valfs) | NGS |  | Deletion | Pathogenic | 376 | 1 |  | Sicily | 2022 |
| c.2536G>T |  | p.(Glu846Ter) | NGS |  | SNV | Pathogenic | 376 | 1 |  | Sicily | 2022 |
| c.2835_2836insCC |  | p.(Ile946Profs) | NGS |  | Insertion | Pathogenic | 376 | 1 |  | Sicily | 2022 |
| c.3253dup |  | p.(Arg1085Lysfs) | NGS |  | Duplication | Pathogenic | 376 | 1 |  | Sicily | 2022 |
| c.4117G>T |  | p.(Glu1373Ter) | NGS |  | SNV | Pathogenic | 376 | 1 |  | Sicily | 2022 |
| c.4357+1G>T |  |  | NGS |  | SNV | Pathogenic | 376 | 1 |  | Sicily | 2022 |
| c.4484G>T |  | p.(Arg1495Met) | NGS |  | SNV | Pathogenic | 376 | 1 |  | Sicily | 2022 |
| c.5035_5039delCTAAT |  | p.(Leu1679Tyrfs) | NGS |  | Deletion | Pathogenic | 376 | 2 |  | Sicily | 2022 |
| c.5266dup |  | p.(Gln1756Profs) | NGS |  | Duplication | Pathogenic | 376 | 1 |  | Sicily | 2022 |
| c.5509T>C |  | p.(Trp1837Arg) | NGS |  | SNV | Pathogenic | 376 | 1 |  | Sicily | 2022 |
| c.5522delG |  | p.(Ser1841fs*) | NGS |  | Deletion | Pathogenic | 376 | 1 |  | Sicily | 2022 |
|  |  |  |  |  |  |  |  |  |  |  |  |
| c.4986+6T>C | Intronic |  | NGS | rs80358086 | Intron | Pathogenic | 137 | 7 | Brahim SM et al. (51) | Mauritania | 2022 |
| c.53T>C | Exon2 | p.Met18Thr | NGS | rs80356929 | Missense | Likely pathogenic | 137 | 1 |  | Mauritania | 2022 |
| c.122A>T | Exon 3 | p.His41Leu | NGS | rs80357276 | Missense | Likely pathogenic | 137 | 1 |  | Mauritania | 2022 |
| c.131G>C | Exon 4 | p.Cys44Ser | NGS | See in ClinVar | Missense | Pathogenic | 137 | 1 |  | Mauritania | 2022 |
| c.5169del | Exon 4 | p.Glu1725LysfsTer5 | NGS | rs80357553 | frameshift | Pathogenic | 137 | 1 |  | Mauritania | 2022 |
| c.3008_3009del | Exon4 | p.Phe1003Ter | NGS | rs80357617 | frameshift | Pathogenic | 137 | 1 |  | Mauritania | 2022 |
| c.813_814insTAGCCATGTG | Exon 4 | p.Glu272Ter | NGS | Unreported | stop_gained | Pathogenic | 137 | 1 |  | Mauritania | 2022 |
| c.815_824dup | Exon 10 | p.Thr276Alafs*14 | NGS | rs387906563 | frameshift | Pathogenic | 137 | 13 |  | Mauritania | 2022 |
|  |  |  |  |  |  |  |  |  |  |  |  |
| c.212+1G>A | 4 |  | NGS +Sanger Sequencing+MLPA | rs80358042 | Splice donor | Pathogenic | 100 | 1 | Rweyemamu LP et al.(52) | Tanzanian | 2022 |
| c.4065_4068del | 10 | p.Asn1355fs | NGS +Sanger Sequencing+MLPA | rs80357508 | Frameshift | Pathogenic | 100 | 1 |  | Tanzanian | 2022 |
| c.2090del | 10 | p.Phe697fs | NGS +Sanger Sequencing+MLPA | rs886039996 | Frameshift | Pathogenic | 100 | 1 |  | Tanzanian | 2022 |
| c.5030_5033del | 16 | p.Thr1677fs | NGS +Sanger Sequencing+MLPA | rs80357580 | Frameshift | Pathogenic | 100 | 1 |  | Tanzanian | 2022 |
| c.5123C>A | 17 | p.Ala1708Glu | NGS +Sanger Sequencing+MLPA | rs28897696 | Missense | Pathogenic | 100 | 1 |  | Tanzanian | 2022 |
|  |  |  |  |  |  |  |  |  |  |  |  |
| c.5309G>T |  | (p.Gly1770Val) | NGS+Sanger |  | Missense | Pathogenic | 184 | 10 | Melki R et al. (53) | Morocco |  |
|  |  |  |  |  |  |  |  |  |  |  |  |
| c.5513T>A | 24 | p.Val1838Glu | NGS | rs80357107 |  | Pathogenic | 6 | 5 | Rioki JN et al. (54) | Kenya | 2022 |
| c.5291T>C | 21 | p.Leu1764Pro | NGS | rs80357281 |  | Pathogenic | 6 | 4 |  | Kenya | 2022 |
| c.5297T>G | 21 | p.Ile1766Ser | NGS | rs80357463 |  | Pathogenic | 6 | 2 |  | Kenya | 2022 |
| c.110C>A | 3 | p.Thr37Lys | NGS | rs80356880 |  | Pathogenic | 6 | 5 |  | Kenya | 2022 |
| c.5212G>C | 20 | p.Gly1738Arg | NGS | rs80356937 |  | Pathogenic | 6 | 5 |  | Kenya | 2022 |
| c.122A>C | 3 | p.His41Pro | NGS | rs80357276 |  | Pathogenic | 6 | 5 |  | Kenya | 2022 |
| c.5117G>A | 18 | p.Gly1706Glu | NGS | rs80356860 |  | Pathogenic | 6 | 5 |  | Kenya | 2022 |
| c.5095C>T | 18 | p.Arg1699Trp | NGS | rs55770810 |  | Pathogenic | 6 | 5 |  | Kenya | 2022 |
| c.5054C>T | 17 | p.Thr1685Ile | NGS | rs80357043 |  | Pathogenic | 6 | 5 |  | Kenya | 2022 |
| c.5053A>G | 17 | p.Thr1685Ala | NGS | rs80356890 |  | Pathogenic | 6 | 5 |  | Kenya | 2022 |
| c.181T>C | 4 | p.Cys61Arg | NGS | rs28897672 |  | Pathogenic/likely pathogenic | 6 | 5 |  | Kenya | 2022 |
| c.131G>T | 3 | p.Cys44Phe | NGS | rs80357446 |  | Pathogenic | 6 | 5 |  | Kenya | 2022 |
| c.115T>A | 3 | p.Cys39Ser | NGS | rs80357164 |  | Pathogenic/likely pathogenic | 6 | 5 |  | Kenya | 2022 |
| c.5143A>G | 18 | p.Se1715Arg | NGS | rs80357222 |  | Pathogenic | 6 | 5 |  | Kenya | 2022 |
| c.130T>G | 3 | p.Cys44Gly | NGS | rs80357327 |  | Pathogenic | 6 | 5 |  | Kenya | 2022 |
| c.5359T>A | 22 | p.Cys1787Ser | NGS | rs80357065 |  | Conflicting interpretations of pathogenicity | 6 | 5 |  | Kenya | 2022 |
| c.5324T>C | 21 | p.Met1775Thr | NGS | rs41293463 |  | Uncertain significance | 6 | 5 |  | Kenya | 2022 |
| c.5123C>T | 18 | p.Ala1708Val | NGS | rs28897696 |  | Uncertain significance | 6 | 5 |  | Kenya | 2022 |
| c.4900A>G | 16 | p.Arg1634Gly | NGS | rs1597830733 |  | Uncertain significance | 6 | 2 |  | Kenya | 2022 |
| c.5332+1G>C | 21 |  | NGS | rs80358041 |  | Pathogenic/likely pathogenic | 6 | 5 |  | Kenya | 2022 |
| c.5278-2del | 21 |  | NGS | rs878853285 |  | Pathogenic | 6 | 5 |  | Kenya | 2022 |
| c.5153-1G>T | 19 |  | NGS | rs80358137 |  | Pathogenic | 6 | 5 |  | Kenya | 2022 |
| c.5152+1G>A | 18 |  | NGS | rs80358094 |  | Pathogenic | 6 | 5 |  | Kenya | 2022 |
| c.5074+1G>T | 17 |  | NGS | rs80358053 |  | Pathogenic | 6 | 5 |  | Kenya | 2022 |
| c.4675+1G>C | 15 |  | NGS | rs80358044 |  | Pathogenic | 6 | 5 |  | Kenya | 2022 |
| c.4357+1G>C | 12 |  | NGS | rs80358027 |  | Likely pathogenic | 6 | 5 |  | Kenya | 2022 |
| c.4097-1G>A | 11 |  | NGS | rs80358070 |  | Pathogenic | 6 | 5 |  | Kenya | 2022 |
| c.547+2T>A | 7 |  | NGS | rs80358047 |  | Pathogenic | 6 | 5 |  | Kenya | 2022 |
| c.302-1G>A |  |  | NGS | rs80358116 |  | Pathogenic | 6 | 5 |  | Kenya | 2022 |
|  |  |  |  |  |  |  |  |  |  |  |  |
| c.2635G>T | 11 | p.Glu879* | NGS | — | SNV/Nonsense | — | 72 | 4 | Zhang et al.(55) | Chinese Hakka | 2022 |
|  |  |  |  |  |  |  |  |  |  |  |  |
| c.894delT |  |  | NGS |  | Frameshift |  | 2216 | 3 | Yu S et al. (56) | Chinese | 2022 |
| c.981_982delAT |  |  | NGS |  | Frameshift |  | 2216 | 1 |  | Chinese | 2022 |
| c.1132delA |  |  | NGS |  | Frameshift |  | 2216 | 3 |  | Chinese | 2022 |
| c.1252G>T |  |  | NGS |  | SNV |  | 2216 | 4 |  | Chinese | 2022 |
| c.1608delT |  |  | NGS |  | Frameshift |  | 2216 | 2 |  | Chinese | 2022 |
| c.1953_1956delGAAA |  |  | NGS |  | Frameshift |  | 2216 | 1 |  | Chinese | 2022 |
| c.1961dupA |  |  | NGS |  | Frameshift |  | 2216 | 1 |  | Chinese | 2022 |
| c.2090_2091delTC |  |  | NGS |  | Frameshift |  | 2216 | 1 |  | Chinese | 2022 |
| c.2110_2111delAA |  |  | NGS |  | Frameshift |  | 2216 | 2 |  | Chinese | 2022 |
| c.2217dupA |  |  | NGS |  | Frameshift |  | 2216 | 1 |  | Chinese | 2022 |
| c.2740G>T |  |  | NGS |  | SNV |  | 2216 | 1 |  | Chinese | 2022 |
| c.2866_2870delTCTCA |  |  | NGS |  | Frameshift |  | 2216 | 1 |  | Chinese | 2022 |
| c.3329delA |  |  | NGS |  | Frameshift |  | 2216 | 1 |  | Chinese | 2022 |
| c.3329dupA |  |  | NGS |  | Frameshift |  | 2216 | 1 |  | Chinese | 2022 |
| c.3359_3363delTTAAT |  |  | NGS |  | Frameshift |  | 2216 | 1 |  | Chinese | 2022 |
| c.3607C>T |  |  | NGS |  | SNV |  | 2216 | 3 |  | Chinese | 2022 |
| c.3770_3771delAG |  |  | NGS |  | Frameshift |  | 2216 | 1 |  | Chinese | 2022 |
| c.3959delC |  |  | NGS |  | Frameshift |  | 2216 | 1 |  | Chinese | 2022 |
| c.4065_4068delTCAA |  |  | NGS |  | Frameshift |  | 2216 | 1 |  | Chinese | 2022 |
| c.4228delG |  |  | NGS |  | Frameshift |  | 2216 | 1 |  | Chinese | 2022 |
| c.4293delC |  |  | NGS |  | Frameshift |  | 2216 | 1 |  | Chinese | 2022 |
| c.4362_4386del25 |  |  | NGS |  | Frameshift |  | 2216 | 1 |  | Chinese | 2022 |
| c.4819G>T |  |  | NGS |  | SNV |  | 2216 | 1 |  | Chinese | 2022 |
| c.5138-1G>T |  |  | NGS |  | Splice site |  | 2216 | 1 |  | Chinese | 2022 |
| c.5154G>A |  |  | NGS |  | SNV |  | 2216 | 3 |  | Chinese | 2022 |
| c.5215+2dupT |  |  | NGS |  | Frameshift |  | 2216 | 2 |  | Chinese | 2022 |
| c.5216-2delA |  |  | NGS |  | Splice site |  | 2216 | 1 |  | Chinese | 2022 |
| c.5470_5477delATTGGGCA |  |  | NGS |  | Frameshift |  | 2216 | 2 |  | Chinese | 2022 |
| c.5533_5540delATTGGGCA |  |  | NGS |  | Frameshift |  | 2216 | 5 |  | Chinese | 2022 |
| c.5566C>T |  |  | NGS |  | SNV |  | 2216 | 1 |  | Chinese | 2022 |
|  |  |  |  |  |  |  |  |  |  |  |  |
| c.3607C>T | 10 | c.3607C>T | NGS | rs62625308 | SNV/Nonsense | Pathogenic | 70 | 1 | Hassan AN et al. (57) | Iraqi Kurdish | 2024 |
| c.3544C>T | 10 | c.3544C>T | NGS | rs80357296 | SNV/Nonsense | Pathogenic | 70 | 1 |  | Iraqi Kurdish | 2024 |
| c.224_227delAAAG | 4 | c.224_227delAAAG | NGS | rs80357697 | Deletion/frameshift | Pathogenic | 70 | 1 |  | Iraqi Kurdish | 2024 |
| c.68_69del | 10 | c.68_69del | NGS | rs80357914 | Deletion/frameshift | Pathogenic | 70 | 1 |  | Iraqi Kurdish | 2024 |
|  |  |  |  |  |  |  |  |  |  |  |  |
| c.178C>T | 3 | p.Gln60Ter | NGS |  | Nonsense | Pathogenic | 1336 | 1 | Al Amri WS et al. (58) | Omani (Middle Eastern) | 2022 |
| c.68-69delAG | 2 | p.Glu23fs | NGS |  | Deletion |  | 1336 | 1 |  | Omani (Middle Eastern) | 2022 |
| c.895_896delGT | 10 | p.Val1299fs | NGS |  | Deletion |  | 1336 | 1 |  | Omani (Middle Eastern) | 2022 |
| c.971G>T | 10 | p.Ser324Ile | NGS |  | Missense | VUS | 1336 | 1 |  | Omani (Middle Eastern) | 2022 |
| c.398G>A | 6 | p.Arg133His | NGS |  | Nonsense |  | 1336 | 1 |  | Omani (Middle Eastern) | 2022 |
| c.4993G>C | 16 | p.Val665Leu | NGS |  | Missense |  | 1336 | 1 |  | Omani (Middle Eastern) | 2022 |
| c.5452-6T>G1 | â€” | p.[?] | NGS |  | Splice site |  | 1336 | 1 |  | Omani (Middle Eastern) | 2022 |
| c.5423T>C | 22 | p.Val1808Ala | NGS |  | Missense |  | 1336 | 1 |  | Omani (Middle Eastern) | 2022 |
| c.4165_4166delAG | 11 | p.Ser1389Ter | NGS |  | Deletion | Pathogenic | 1336 | 1 |  | Omani (Middle Eastern) | 2022 |
| c.2123C>A | 10 | p.Ser708Tyr | NGS |  | Nonsense | VUS | 1336 | 1 |  | Omani (Middle Eastern) | 2022 |
|  |  |  |  |  |  |  |  |  |  |  |  |
| c.1674del |  | p.(Gly559ValfsTer13) | NGS |  | Frameshift | Pathogenic | 307 | 4 | Rojas LXR et al. (59) | Colombian | 2022 |
| c.3331_3334del |  | p.(Gln1111AsnfsTer5) | NGS |  | Frameshift | Pathogenic | 307 | 4 |  | Colombian | 2022 |
| c.5093_5096del |  | p.(Thr1698IlefsTer2) | NGS |  | Frameshift | Pathogenic | 307 | 1 |  | Colombian | 2022 |
| c.5177_5180del |  | p.(Arg1726LysfsTer3) | NGS |  | Frameshift | Pathogenic | 307 | 1 |  | Colombian | 2022 |
|  |  |  |  |  |  |  |  |  |  |  |  |
| c.66_67delAG | 2 | p.Glu23Val |  | - | Deletion | Frameshift | 79 | 1 | Shah ND et al. (31) | Indian | 2018 |
| c.3119G>A | 10 | p.Ser1040Asn |  | rs4986852 | SNP | Missense | 79 | 2 |  | Indian | 2018 |
| c.2412G>C | 11 | p.Gln804His |  | rs55746541 | SNP | Missense | 79 | 1 |  | Indian | 2018 |
| c.5019G>A | 16 | p.Met1673Ile |  | rs1799967 | SNP | Missense | 79 | 2 |  | Indian | 2018 |
| c.3113A>G | 10 | p.Glu1038Gly |  | rs16941 | SNP | Missense | 79 | 7 |  | Indian | 2018 |
| c.2077G>A | 9 | p.Asp693Asn |  | rs4986850 | SNP | Missense | 79 | 2 |  | Indian | 2018 |
| c.2612C>T | 11 | p.Pro871Leu |  | rs799917 | SNP | Missense | 79 | 6 |  | Indian | 2018 |
| c.2521C>T | 11 | p.Arg841Try |  | rs1800709 | SNP | Missense | 79 | 1 |  | Indian | 2018 |
| c.3328_3330delAAG | 10 | p.Lys1110del |  | rs80358335 | Deletion | In-frame del. | 79 | 2 |  | Indian | 2018 |
| c.4442_4442delG | 14 | p.Ser1481fs |  | NOVEL | Deletion | Frameshift | 79 | 2 |  | Indian | 2018 |
| c.5137+1G>A | 3 | - |  | Novel | Splice site | Missense | 79 | 2 |  | Indian | 2018 |
