# Supplementary material for: Global prevalence and ethnic variation of pathogenic BRCA1/2 variants in breast cancer: a systematic review and meta-analysis
Source: J Transl Med. 2026 Mar 12;24:555. doi: 10.1186/s12967-026-07997-3 (PMC13097826; doi:10.1186/s12967-026-07997-3)
Supplement: Supplementary file 5 — Supplementary Material 5 [file 12967_2026_7997_MOESM5_ESM.docx]

**Supplementary Table S3. List of BRCA2 Variants across different ethnicities**

| **Variant ID** | **Exon** | **Protein Change** | **Detection Method** | **dbSNP150** | **Mutation Type** | **Variant impact** | **Cases tested** | **Carrier number** | **References (number in Supplementary table 1)** | **Ethnicity/Population** | **Year of Study** |
| --- | --- | --- | --- | --- | --- | --- | --- | --- | --- | --- | --- |
| c.5718_5719delCT | 11 | p.Asn1906_Ser1907fs | Sanger |  | Frameshift | Pathogenic | 200 | 1 | Dodova RI et al. (16) | Bulgarian | 2015 |
| c.5851_5854delAGTT | 11 | p.Ser1951_Leu1952delinsTrpfs | Sanger |  | Frameshift | Pathogenic | 200 | 2 |  | Bulgarian | 2015 |
| c.5946delT | 11 | p.Ser1982Argfs | Sanger |  | Frameshift | Pathogenic | 200 | 1 |  | Bulgarian | 2015 |
| c.7910_7914delCCTTT | 17 | p.Ala2637_Phe2638delinsAlafs | Sanger |  | Frameshift | Pathogenic | 200 | 1 |  | Bulgarian | 2015 |
| c.8532_8533delAA | 20 | p.Glu2844fs | Sanger |  | Frameshift | Pathogenic | 200 | 1 |  | Bulgarian | 2015 |
| c.9098_9099insA | 23 | p.Thr3033delinsThrSerfs | Sanger |  | Frameshift | Pathogenic | 200 | 4 |  | Bulgarian | 2015 |
| c.9682delA | 27 | p.Gln3227fs | Sanger |  | Frameshift | Pathogenic | 200 | 1 |  | Bulgarian | 2015 |
|  |  |  |  |  |  |  |  |  |  |  |  |
| c.7934delG | 17 | c.7934delG / p.Arg2645fs | NGS | - | Frameshift |  | 108 | 2 | Francies FZ et al.(17) | White South African | 2015 |
| c.9097_9098insA | 23 | c.9097_9098insA / p.Thr3033fs | NGS | - | Frameshift | Pathogenic | 108 | 1 |  | Black South African | 2015 |
| c.8754+1G>A | 21 | c.8754+1G>A / Splice site | NGS | - | Splice site | Pathogenic | 108 | 1 |  | Indian South African | 2015 |
|  |  |  |  |  |  |  |  |  |  |  |  |
| c.674delC | 8 | c.674delC / p.Thr225Ilefs*5 | Sanger / NGS | - | Frameshift | Pathogenic | 250 | 1 | El Saghir NS et al. (18) | Lebanese | 2015 |
| c.1310_1313del | 10 | c.1310_1313del / p.Lys437Ilefs*22 | Sanger / NGS | - | Frameshift | Pathogenic | 250 | 1 |  | Lebanese | 2015 |
| c.3971del | 11 | c.3971del / p.Tyr1324Leufs*11 | Sanger / NGS | - | Frameshift | Pathogenic | 250 | 1 |  | Lebanese | 2015 |
| c.5576_5579del | 11 | c.5576_5579del / p.Ile1859Lysfs*3 | Sanger / NGS | - | Frameshift | Pathogenic | 250 | 1 |  | Lebanese | 2015 |
| c.9257-1G>A | IVS24 | c.9257-1G>A / p.? | Sanger / NGS | - | Splice site | Pathogenic | 250 | 3 |  | Lebanese | 2015 |
|  |  |  |  |  |  |  |  |  |  |  |  |
| Q742X | 11 | c.2254C>T / p.Gln742* | Sanger / NGS | - | Nonsense | Pathogenic | 190 | 1 | Villarreal-Garza C et al. (19) | Mexican | 2015 |
|  |  |  |  |  |  |  |  |  |  |  |  |
| c.115delG | 3 | c.115delG | MLPA |  | F |  | 396 | 1 | Pal T et al. (20) | Black Women (Florida) | 2015 |
| c.1103C>G | 10 | c.1103C>G | MLPA |  | N |  | 396 | 1 |  | Black Women (Florida) | 2015 |
| c.1705_1706delCA | 10 | c.1705_1706delCA | MLPA |  | F |  | 396 | 1 |  | Black Women (Florida) | 2015 |
| c.1887_1893delTACATTT | 10 | c.1887_1893delTACATTT | MLPA |  | F |  | 396 | 1 |  | Black Women (Florida) | 2015 |
| c.3599_3600delGT | 11 | c.3599_3600delGT | MLPA |  | F |  | 396 | 1 |  | Black Women (Florida) | 2015 |
| c.3680_3681delTG | 11 | c.3680_3681delTG | MLPA |  | F |  | 396 | 1 |  | Black Women (Florida) | 2015 |
| c.4471_4474delCTGA | 11 | c.4471_4474delCTGA | MLPA |  | F |  | 396 | 2 |  | Black Women (Florida) | 2015 |
| c.5616_5620delAGTAA | 11 | c.5616_5620delAGTAA | MLPA |  | F |  | 396 | 1 |  | Black Women (Florida) | 2015 |
| c.5979_5980insA | 11 | c.5979_5980insA | MLPA |  | F |  | 396 | 1 |  | Black Women (Florida) | 2015 |
| c.6137C>A | 11 | c.6137C>A | MLPA |  | N |  | 396 | 1 |  | Black Women (Florida) | 2015 |
| c.8777T>A | 22 | c.8777T>A | MLPA |  | N |  | 396 | 1 |  | Black Women (Florida) | 2015 |
| c.8969G>A | 23 | c.8969G>A | MLPA |  | N |  | 396 | 1 |  | Black Women (Florida) | 2015 |
| c.9253_9254insA | 24 | c.9253_9254insA | MLPA |  | F |  | 396 | 1 |  | Black Women (Florida) | 2015 |
| c.9382C>T | 25 | c.9382C>T | MLPA |  | N |  | 396 | 1 |  | Black Women (Florida) | 2015 |
|  |  |  |  |  |  |  |  |  |  |  |  |
| c.6025C>T | 11 | c.6025C>T / p.Gln2009X | Capture/Sanger Sequencing | - | Nonsense | Pathogenic | 818 | 3 | Abdikhakimov A et al.(21) | Middle Eastern (Saudi Arabia) | 2016 |
| c.7007G>A | 13 | c.7007G>A / p.Arg2336His | Capture/Sanger Sequencing | - | Missense | Pathogenic | 818 | 1 |  | Middle Eastern (Saudi Arabia) | 2016 |
|  |  |  |  |  |  |  |  |  |  |  |  |
| c.943T>A | 10 | c.943T>A / p.Cys315Ser | Direct sequencing | rs79483201 | Missense | Unclassified | 328 | 3 | Yoon KA et al. (23) | Korean | 2017 |
| c.1745T>C | 11 | c.1745T>C / p.Thr582Pro | Direct sequencing | rs80358457 | Missense | Unclassified | 328 | 6 |  | Korean | 2017 |
| c.2350A>G | 11 | c.2350A>G / p.Met784Val | Direct sequencing | rs11571653 | Missense | Unclassified | 328 | 9 |  | Korean | 2017 |
| c.3220A>T | 11 | c.3220A>T / p.Asp1074Val | Direct sequencing | rs14505603 | Missense | Unclassified | 328 | 2 |  | Korean | 2017 |
| c.6020T>G | 11 | c.6020T>G / p.Val2010Gly | Direct sequencing | - | Missense | Unclassified | 328 | 2 |  | Korean | 2017 |
| c.6325G>C | 11 | c.6325G>C / p.Val2109Leu | Direct sequencing | rs79456940 | Missense | Unclassified | 328 | 3 |  | Korean | 2017 |
| c.6351G>T | 11 | c.6351G>T / p.Ala2151Gly | Direct sequencing | - | Missense | Unclassified | 328 | 3 |  | Korean | 2017 |
| c.7522G>C | 15 | c.7522G>C / p.Gly2508Ser | Direct sequencing | - | Missense | Unclassified | 328 | 2 |  | Korean | 2017 |
| c.8187G>T | 18 | c.8187G>T / p.Lys2729Asn | Direct sequencing | rs80359065 | Missense | Unclassified | 328 | 10 |  | Korean | 2017 |
|  |  |  |  |  |  |  |  |  |  |  |  |
| Met591Ile | - | - | NGS | rs80359304 | Frameshift | Pathogenic | 31 | 1 | Ricks-Santi L et al. (24) | African American | 2017 |
| Asp596His | - | - | NGS | rs56328701 | Missense | VUS | 31 | 1 |  | African American | 2017 |
| Arg2973Cys | - | - | NGS | rs45469092 | Missense | VUS | 31 | 1 |  | African American | 2017 |
| Val2171Val | - | - | NGS | rs206076 | Synonymous | VUS | 31 | 31 |  | African American | 2017 |
| c.7007+53G>A | - | c.7007+53G>A | NGS | rs56014558 | Intron | VUS | 31 | 1 |  | African American | 2017 |
| c.7008-62A>G | - | c.7008-62A>G | NGS | rs76584943 | Intron | VUS | 31 | 1 |  | African American | 2017 |
|  |  |  |  |  |  |  |  |  |  |  |  |
| c.4827delTG | - | c.4827delTG / p.T1609fs | Sequencing | - | Frameshift | Pathogenic | 4 | 1 | Walsh T et al. (25) | Ashkenazi Jewish | 2003 |
| c.5718delCT | - | c.5718delCT / p.N1906fs | Sequencing | - | Frameshift | Pathogenic | 4 | 1 |  | Ashkenazi Jewish | 2003 |
| c.8635del5 | - | c.8635del5 / p.N2879fs | Sequencing | - | Frameshift | Pathogenic | 4 | 1 |  | Ashkenazi Jewish | 2003 |
| c.9090dupA | - | c.9090dupA / p.T3030fs | Sequencing | - | Frameshift | Pathogenic | 4 | 1 |  | Ashkenazi Jewish | 2003 |
|  |  |  |  |  |  |  |  |  |  |  |  |
| W194X |  | c.6025C>T | Targeted Sequencing | rs80358810 | nonsense | pathogenic | 467 | 1 | Yang XR et al. (26) | Malaysian (Sarawak) | 2017 |
| c.657delT |  | c.657delT | Targeted Sequencing |  | frameshift | pathogenic | 467 | 1 |  | Malaysian (Sarawak) | 2017 |
| c.755_758delACAG |  | c.755_758delACAG | Targeted Sequencing | rs80359659 | frameshift | pathogenic | 467 | 1 |  | Malaysian (Sarawak) | 2017 |
| c.1763_1766delATAA |  | c.1763_1766delATAA | Targeted Sequencing | rs80359303 | frameshift | pathogenic | 467 | 1 |  | Malaysian (Sarawak) | 2017 |
| c.1888dupA |  | c.1888dupA | Targeted Sequencing | rs80359314 | frameshift | pathogenic | 467 | 1 |  | Malaysian (Sarawak) | 2017 |
| c.2442delC |  | c.2442delC | Targeted Sequencing | rs397507627 | frameshift | pathogenic | 467 | 1 |  | Malaysian (Sarawak) | 2017 |
| Q1037X |  | c.3109C>T | Targeted Sequencing | rs80358557 | nonsense | pathogenic | 467 | 1 |  | Malaysian (Sarawak) | 2017 |
| c.5335_5334delAC |  | c.5335_5334delAC | Targeted Sequencing | rs397507780 | frameshift | pathogenic | 467 | 3 |  | Malaysian (Sarawak) | 2017 |
| c.5575_5578delATTA |  | c.5575_5578delATTA | Targeted Sequencing | rs80359520 | frameshift | pathogenic | 467 | 1 |  | Malaysian (Sarawak) | 2017 |
| c.8961_8964delGAGT |  | c.8961_8964delGAGT | Targeted Sequencing | rs80359734 | frameshift | pathogenic | 467 | 4 |  | Malaysian (Sarawak) | 2017 |
|  |  |  |  |  |  |  |  |  |  |  |  |
| 3034delCAAA | - | c.2808_2811delACAA | Sequencing | - | Frameshift | Pathogenic | 853 | 8 | Briceño-Balcázar I et al. (27) | Colombian | 2014 |
| 6076delGTCTT | - | c.5846_5850delGTCTT | Sequencing | - | Frameshift | Pathogenic | 853 | 2 |  | Colombian | 2014 |
| 6503delTTT | - | c.6274_6276delTTT | Sequencing | - | Frameshift | Pathogenic | 853 | 1 |  | Colombian | 2014 |
| c.936G>A | - | p.W312X | Sequencing | - | Nonsense | Pathogenic | 853 | 1 |  | Colombian | 2014 |
| T289A | - | T289A | Sequencing | - | Missense | NR | 853 | 1 |  | Colombian | 2014 |
| C6448A | - | C6448A | Sequencing | - | Missense | Benign | 853 | 1 |  | Colombian | 2014 |
| C3046T | - | C3046T | Sequencing | - | Missense | Pathogenic | 853 | 1 |  | Colombian | 2014 |
| V572L | - | V572L | Sequencing | - | Missense | Uncertain | 853 | 1 |  | Colombian | 2014 |
| P218L | - | P218L | Sequencing | - | Missense | NR | 853 | 1 |  | Colombian | 2014 |
| C6328T | - | C6328T | Sequencing | - | Missense | Benign | 853 | 3 |  | Colombian | 2014 |
| T10K | - | T10K | Sequencing | - | Missense | Uncertain | 853 | 1 |  | Colombian | 2014 |
| 2929delC | - | 2929delC | Sequencing | - | Frameshift | Benign | 853 | 1 |  | Colombian | 2014 |
| 3154TC>AT | - | 3154TC>AT | Sequencing | - | Substitution | NR | 853 | 1 |  | Colombian | 2014 |
| C5972T | - | C5972T | Sequencing | - | Missense | Benign | 853 | 11 |  | Colombian | 2014 |
| T1011R | - | T1011R (3260C>G) | Sequencing | - | Missense | Conflicting interpretations | 853 | 1 |  | Colombian | 2014 |
| 4772delA | - | 4772delA | Sequencing | - | Frameshift | NR | 853 | 1 |  | Colombian | 2014 |
| 6310delGA | - | 6310delGA | Sequencing | - | Frameshift | Benign | 853 | 1 |  | Colombian | 2014 |
| A5996C | - | A5996C | Sequencing | - | Missense | Conflicting interpretations | 853 | 1 |  | Colombian | 2014 |
| 6062insG | - | 6062insG | Sequencing | - | Insertion | NR | 853 | 1 |  | Colombian | 2014 |
| S1630X | - | S1630X (5117C>G) | Sequencing | - | Nonsense | NR | 853 | 1 |  | Colombian | 2014 |
| N570S | - | N570S (1937A>G) | Sequencing | - | Missense | Uncertain | 853 | 1 |  | Colombian | 2014 |
|  |  |  |  |  |  |  |  |  |  |  |  |
| c.5753delA | - | c.5753delA / p.H1918fs | NGS | - | Frameshift | Pathogenic | 71 | 2 | Fang M et al.(28) | Chinese | 2017 |
| c.8400_8402delTT | - | c.8400_8402delTT / p.2800_2801del | NGS | - | Frameshift | Pathogenic | 71 | 1 |  | Chinese | 2017 |
| TinsAAAA | - | TinsAAAA | NGS | - | Insertion | Pathogenic | 71 | 1 |  | Chinese | 2017 |
| c.3883C>T | - | c.3883C>T / p.Q1295X | NGS | - | Nonsense | Pathogenic | 71 | 1 |  | Chinese | 2017 |
| c.5495delC | - | c.5495delC / p.S1832fs | NGS | - | Frameshift | Pathogenic | 71 | 1 |  | Chinese | 2017 |
| c.2806_2809delAAAC | - | c.2806_2809delAAAC / p.K936fs | NGS | - | Frameshift | Pathogenic | 71 | 1 |  | Chinese | 2017 |
|  |  |  |  |  |  |  |  |  |  |  |  |
| c.5164_5165delAG | 11 | p.Ser1722Tyrfs | NGS + Sanger | - | Frameshift | Pathogenic | 595 | 1 | Liang Y et al. (29) | Chinese | 2018 |
| c.182_182delT | 3 | p.Leu61fs | NGS + Sanger | - | Frameshift | Pathogenic | 595 | 1 |  | Chinese | 2018 |
| c.3109C>T | 11 | p.Gln1037Ter | NGS + Sanger | - | Nonsense | Pathogenic | 595 | 1 |  | Chinese | 2018 |
| c.2806_2809delAAAC | 11 | p.Lys936_Gln937fs | NGS + Sanger | - | Frameshift | Pathogenic | 595 | 1 |  | Chinese | 2018 |
| c.5718_5719delCT | 11 | p.Leu1908fs | NGS + Sanger | - | Frameshift | Pathogenic | 595 | 1 |  | Chinese | 2018 |
| c.5959C>T | 11 | p.Gln1987Ter | NGS + Sanger | - | Nonsense | Pathogenic | 595 | 1 |  | Chinese | 2018 |
| c.9400_9400delG | 25 | p.Gly3134fs | NGS + Sanger | - | Frameshift | Pathogenic | 595 | 1 |  | Chinese | 2018 |
| c.469_473delAAGTC | 5 | p.Val159fs | NGS + Sanger | - | Frameshift | Pathogenic | 595 | 1 |  | Chinese | 2018 |
| c.304_304delA | 3 | p.Leu103fs | NGS + Sanger | - | Frameshift | Pathogenic | 595 | 1 |  | Chinese | 2018 |
| c.7480C>T | 15 | p.Arg2494Ter | NGS + Sanger | - | Nonsense | Pathogenic | 595 | 1 |  | Chinese | 2018 |
| c.3559G>T | 11 | p.Glu1187Ter | NGS + Sanger | - | Nonsense | Pathogenic | 595 | 1 |  | Chinese | 2018 |
| c.8955_8956insA | 23 | p.Ile2986fs | NGS + Sanger | - | Frameshift | Pathogenic | 595 | 1 |  | Chinese | 2018 |
| c.8827C>T | 22 | p.Gln2943Ter | NGS + Sanger | - | Nonsense | Pathogenic | 595 | 1 |  | Chinese | 2018 |
| c.464_468delGAGAT | 5 | p.Arg155fs | NGS + Sanger | - | Frameshift | Pathogenic | 595 | 1 |  | Chinese | 2018 |
| c.8517C>A | 20 | p.Tyr2839Ter | NGS + Sanger | - | Nonsense | Pathogenic | 595 | 1 |  | Chinese | 2018 |
| c.5574_5577delAATT | 11 | p.Ile1859fs | NGS + Sanger | - | Frameshift | Pathogenic | 595 | 1 |  | Chinese | 2018 |
| c.3163_3166delAATC | 11 | p.Gly2281fs | NGS + Sanger | - | Frameshift | Pathogenic | 595 | 1 |  | Chinese | 2018 |
| c.5900_5901insG | 11 | p.Ser1968fs | NGS + Sanger | - | Frameshift | Pathogenic | 595 | 1 |  | Chinese | 2018 |
| c.8576_8576delA | 20 | p.Lys2860fs | NGS + Sanger | - | Frameshift | Pathogenic | 595 | 1 |  | Chinese | 2018 |
| c.9317G>A | 25 | p.Trp3106Ter | NGS + Sanger | - | Nonsense | Pathogenic | 595 | 1 |  | Chinese | 2018 |
| c.8951C>G | 22 | p.Ser2984Ter | NGS + Sanger | - | Nonsense | Pathogenic | 595 | 1 |  | Chinese | 2018 |
| c.1301_1304delAAAG | 10 | p.Lys437fs | NGS + Sanger | - | Frameshift | Pathogenic | 595 | 1 |  | Chinese | 2018 |
| c.6952C>T | 13 | p.Arg2318Ter | NGS + Sanger | - | Nonsense | Pathogenic | 595 | 1 |  | Chinese | 2018 |
| c.5718_5721delCTCT | 11 | p.Ser1907fs | NGS + Sanger | - | Frameshift | Pathogenic | 595 | 1 |  | Chinese | 2018 |
| c.7562_7563delTC | 15 | p.Leu2523fs | NGS + Sanger | - | Frameshift | Pathogenic | 595 | 1 |  | Chinese | 2018 |
|  |  |  |  |  |  |  |  |  |  |  |  |
| 999del5 | - | 999del5 / Stop 273 | NGS/Sanger | - | Frameshift | Deleterious | 100 | 1 | Abdel-Razeq H et al. (30) | Jordanian | 2018 |
| 1461insA | - | 1461insA / Stop 420 | NGS/Sanger | - | Frameshift | Deleterious | 100 | 2 |  | Jordanian | 2018 |
| 2482del4 | - | 2482del4 / Stop 770 | NGS/Sanger | - | Frameshift | Deleterious | 100 | 4 |  | Jordanian | 2018 |
| L2039X | - | c.6344T>A / L2039X | NGS/Sanger | - | Nonsense | Deleterious | 100 | 1 |  | Jordanian | 2018 |
| 6855del8 | - | 6855del8 / Stop 2221 | NGS/Sanger | - | Frameshift | Deleterious | 100 | 1 |  | Jordanian | 2018 |
| 6862del4 | - | 6862del4 / Stop 2227 | NGS/Sanger | - | Frameshift | Deleterious | 100 | 1 |  | Jordanian | 2018 |
| E2229X | - | c.6913G>T / E2229X | NGS/Sanger | - | Nonsense | Deleterious | 100 | 1 |  | Jordanian | 2018 |
| IVS23-1G>A | - | IVS23-1G>A | NGS/Sanger | - | Splice | Deleterious | 100 | 1 |  | Jordanian | 2018 |
| IVS24-1G>A | - | IVS24-1G>A | NGS/Sanger | - | Splice | Suspected Deleterious | 100 | 3 |  | Jordanian | 2018 |
| dup exons 5–11 | - | dup exons 5–11(5′) | NGS/Sanger | - | Duplication | Suspected Deleterious | 100 | 4 |  | Jordanian | 2018 |
| P168A | - | c.730C>G / P168A | NGS/Sanger | - | Missense | VUS | 100 | 1 |  | Jordanian | 2018 |
| T251R | - | c.980C>G / T251R | NGS/Sanger | - | Missense | VUS | 100 | 1 |  | Jordanian | 2018 |
| A2306P | - | c.7144G>C / A2306P | NGS/Sanger | - | Missense | VUS | 100 | 1 |  | Jordanian | 2018 |
| Q2925R | - | c.9002A>G / Q2925R | NGS/Sanger | - | Missense | VUS | 100 | 2 |  | Jordanian | 2018 |
| E2193K | - | c.6805G>A / E2193K | NGS/Sanger | - | Missense | VUS | 100 | 1 |  | Jordanian | 2018 |
| K21R | - | c.290A>G / K21R | NGS/Sanger | - | Missense | FP | 100 | 0 |  | Jordanian | 2018 |
| K3416E | - | c.10474A>G / K3416E | NGS/Sanger | - | Missense | FP | 100 | 2 |  | Jordanian | 2018 |
|  |  |  |  |  |  |  |  |  |  |  |  |
| 7643delAT | - | - | NGS/Sanger | - | Frameshift | Pathogenic | 310 | 1 | Abulkhair O et al. (32) | Saudi Arabian | 2018 |
| c.2808_2811 | - | - | NGS/Sanger | - | Deletion | Pathogenic | 310 | 1 |  | Saudi Arabian | 2018 |
| c.5034_504del | - | - | NGS/Sanger | - | Deletion | Pathogenic | 310 | 1 |  | Saudi Arabian | 2018 |
| c.6591_6592delTG | - | - | NGS/Sanger | - | Deletion | Pathogenic | 310 | 1 |  | Saudi Arabian | 2018 |
| c.8332_1G>T | - | - | NGS/Sanger | - | Splice | Pathogenic | 310 | 1 |  | Saudi Arabian | 2018 |
| c.9502_131G>A | - | - | NGS/Sanger | - | Missense | Pathogenic | 310 | 1 |  | Saudi Arabian | 2018 |
| c.968_971 | - | - | NGS/Sanger | - | Deletion | Pathogenic | 310 | 1 |  | Saudi Arabian | 2018 |
|  |  |  |  |  |  |  |  |  |  |  |  |
| c.2971A>G | 11 | A>G | NGS | - | Missense | Good prognosis | 82 | 7 | Wang T et al. (33) | Chinese | 2019 |
| c.31delT | - | DelT | NGS | - | Frameshift | Potential Pathogenic | 82 | 1 |  | Chinese | 2019 |
| c.6408delA | - | DelA | NGS | - | Frameshift | Potential Pathogenic | 82 | 1 |  | Chinese | 2019 |
| c.6705delG | - | DelG | NGS | - | Frameshift | Potential Pathogenic | 82 | 1 |  | Chinese | 2019 |
| c.677delC | - | DelC | NGS | - | Frameshift | Potential Pathogenic | 82 | 1 |  | Chinese | 2019 |
| c.3109C>T | 11 | C>T | NGS | - | Nonsense | Pathogenic | 82 | 1 |  | Chinese | 2019 |
|  |  |  |  |  |  |  |  |  |  |  |  |
| c.67+2T>C | Intron 2 | Splice donor site | NGS | rs81002885 | Splice site | Deleterious | 25 | 1 | Al Hannan F et al. (34) | Bahraini | 2019 |
|  |  |  |  |  |  |  |  |  |  |  |  |
| c.10234A>G | 27 | p.Ile3412Val | PGM & Miseq |  | Missense_variant |  | 54 | 4 | Shen M et al.(36) | Chinese | 2019 |
| c.9401delG | 25 | p.Gly3134AlafsTer29 | PGM & Miseq |  | Frameshift_variant | Pathogenic | 54 | 1 |  | Chinese | 2019 |
| c.8187G>T | 18 | p.Lys2729Asn | PGM & Miseq |  | Missense_variant |  | 54 | 1 |  | Chinese | 2019 |
| c.5852G>A | 11 | p.Ser1951Asn | PGM & Miseq |  | Missense_variant | Uncertain | 54 | 1 |  | Chinese | 2019 |
| c.5785A>G | 11 | p.Ile1929Val | PGM & Miseq |  | Missense_variant |  | 54 | 2 |  | Chinese | 2019 |
| c.2971A>G | 11 | p.Asn991Asp | PGM & Miseq |  | Missense_variant |  | 54 | 15 |  | Chinese | 2019 |
| c.1462A>G | 10 | p.Ile488Val | PGM & Miseq |  | Missense_variant | Uncertain | 54 | 1 |  | Chinese | 2019 |
| c.1399A>T | 10 | p.Lys467Te | PGM & Miseq |  | Stop_gained | Pathogenic | 54 | 1 |  | Chinese | 2019 |
| c.1114A>C | 10 | p.Asn372His | PGM & Miseq |  | Missense_variant |  | 54 | 31 |  | Chinese | 2019 |
| c.865A>C | 10 | p.Asn289His | PGM & Miseq |  | Missense_variant |  | 54 | 15 |  | Chinese | 2019 |
| c.461A>G | 5 | p.Gln154Arg | PGM & Miseq |  | Missense_variant | Uncertain | 54 | 1 |  | Chinese | 2019 |
| c.10150C>G | 27 | p.Arg3384Gly | PGM & Miseq |  | Missense_variant |  | 54 | 1 |  | Chinese | 2019 |
| c.9294C>G | 25 | p.Tyr3098Ter | PGM & Miseq |  | Stop_gained | Pathogenic | 54 | 1 |  | Chinese | 2019 |
| c.7414_7415delAA | 14 | p.Lys2472ValfsTer2 | PGM & Miseq |  | Frameshift_variant | Pathogenic | 54 | 1 |  | Chinese | 2019 |
| c.3445A>G | 11 | p.Met1149Val | PGM & Miseq |  | Missense_variant | Pathogenic | 54 | 1 |  | Chinese | 2019 |
|  |  |  |  |  |  |  |  |  |  |  |  |
| c.9413dupT |  |  | NGS |  | Frameshift |  | 99 | 2 | Geredeli C et al. (37) | Turkish | 2019 |
| c.67+1G>A |  | IVS2 +1G>A | NGS |  | Splice site |  | 99 | 2 |  | Turkish | 2019 |
| c.771 775deTCAA |  |  | NGS |  | Frameshift |  | 99 | 1 |  | Turkish | 2019 |
| c.7487dupA |  |  | NGS |  | Frameshift |  | 99 | 1 |  | Turkish | 2019 |
| c.3318C>G |  | p.S1106R | NGS |  | Missense |  | 99 | 1 |  | Turkish | 2019 |
| c.9317G>A |  | P.W3106X | NGS |  | Missense |  | 99 | 1 |  | Turkish | 2019 |
|  |  |  |  |  |  |  |  |  |  |  |  |
| c.1909-37dup |  | NA | NGS |  | Intronic | Intronic | 231 | 1 | Cortés C et al. (38) | Columbia | 2019 |
| c.1909-33delA |  | NA | NGS |  | Intronic | Intronic | 231 | 9 |  | Columbia | 2019 |
| c.2147A>G |  | p.Gln716Arg | NGS |  | Missense | Non-synonymous | 231 | 1 |  | Columbia | 2019 |
| c.2229T>C |  | p.His743His | NGS |  | Synonymous | Synonymous | 231 | 2 |  | Columbia | 2019 |
| c.2313A>T |  | p.Leu771Phe | NGS |  | Missense | Non-synonymous | 231 | 1 |  | Columbia | 2019 |
| c.2386G>A |  | p.Asp796Asn | NGS |  | Missense | Non-synonymous | 231 | 18 |  | Columbia | 2019 |
| c.2454T>A |  | p.Asn818Lys | NGS |  | Missense | Non-synonymous | 231 | 1 |  | Columbia | 2019 |
| c.2574_2575insA |  | p.Val859Ser*22 | NGS |  | Nonsense | Nonsense | 231 | 1 |  | Columbia | 2019 |
| c.2971A>G |  | p.Asn991Asp | NGS |  | Missense | Non-synonymous | 231 | 7 |  | Columbia | 2019 |
| c.3095A>T |  | p.Lys1032Ile | NGS |  | Missense | Non-synonymous | 231 | 1 |  | Columbia | 2019 |
| c.3396T>G |  | p.Lys1132Lys | NGS |  | Synonymous | Synonymous | 231 | 23 |  | Columbia | 2019 |
| c.3807T>C |  | p.Val1269Val | NGS |  | Synonymous | Synonymous | 231 | 8 |  | Columbia | 2019 |
| c.4563A>G |  | p.Leu1521Leu | NGS |  | Synonymous | Synonymous | 231 | 80 |  | Columbia | 2019 |
| c.6513C>G |  | p.Val2171Val | NGS |  | Synonymous | Synonymous | 231 | 75 |  | Columbia | 2019 |
| c.6841+80_6841+83delTTAA |  | NA | NGS |  | Intronic | Intronic | 231 | 3 |  | Columbia | 2019 |
|  |  |  |  |  |  |  |  |  |  |  |  |
| c.145G>T |  | p.Glu49Ter | NGS | rs80358435 | Nonsense | Pathogenic | 252 | 1 | Millan Catalan O et al.(41) | Latin American | 2022 |
| c.1806insA |  | p.Gly602fs | NGS | rs80359307 | Frameshift | Pathogenic | 252 | 1 |  | Latin American | 2022 |
| c.1813delA |  | p.Ile605fs | NGS | rs80359306 | Frameshift | Pathogenic | 252 | 2 |  | Latin American | 2022 |
| c.2899_2900delCT |  | p.Leu967Argfs | NGS | rs80359361 | Frameshift | Pathogenic | 252 | 1 |  | Latin American | 2022 |
| c.3166C>T |  | p.Gln1056Ter | NGS | rs79728106 | Nonsense | Pathogenic | 252 | 1 |  | Latin American | 2022 |
| c.3492insT |  | p.Gln1089fs | NGS | rs80359380 | Frameshift | Pathogenic | 252 | 1 |  | Latin American | 2022 |
| c.5631delC |  | p.Asn1877fs | NGS | rs397507357 | Frameshift | Pathogenic | 252 | 1 |  | Latin American | 2022 |
| c.6244_6244delG |  |  | NGS |  | Frameshift | Pathogenic | 252 | 1 |  | Latin American | 2022 |
| c.6024_6025insG |  | p.Gln2009fs | NGS | rs80359554 | Frameshift | Pathogenic | 252 | 1 |  | Latin American | 2022 |
| c.6486_6489delACAA |  | p.Lys2162fs | NGS | rs80359598 | Frameshift | Pathogenic | 252 | 1 |  | Latin American | 2022 |
| c.8219T>G |  | p.Leu2740Ter | NGS | rs80359070 | Nonsense | Pathogenic | 252 | 1 |  | Latin American | 2022 |
| c.8754G>A |  | p.Glu2918= | NGS | rs80359803 | Splice | Pathogenic | 252 | 1 |  | Latin American | 2022 |
|  |  |  |  |  |  |  |  |  |  |  |  |
| c.2588dupA |  | p.(Asn863LysfsTer18) |  |  |  |  | 555 | 1 | Behl S et al. (43) | French-Canadians | 2020 |
| c.2806_2809delAAAC |  | p.(Ala938ProfsTer21) |  |  |  |  | 555 | 0 |  | French-Canadians | 2020 |
| c.3170_3174delAGAAA |  | p.(Lys1057TrfsTer8) |  |  |  |  | 547 | 6 |  | French-Canadians | 2020 |
| c.3545_3546delTT |  | p.(Phe1182Ter) |  |  |  |  | 550 | 2 |  | French-Canadians | 2020 |
| c.5857G > T |  | p.(Glu1953Ter) |  |  |  |  | 555 | 1 |  | French-Canadians | 2020 |
| c.6275_6276delTT |  | p.(Leu2092ProfsTer7) |  |  |  |  | 555 | 0 |  | French-Canadians | 2020 |
| c.8537_8538delAG |  | p.(Glu2846GlyfsTer22) |  |  |  |  | 536 | 7 |  | French-Canadians | 2020 |
| c.9004G > A |  | p.(Glu3002Lys) |  |  |  |  | 554 | 0 |  | French-Canadians | 2020 |
|  |  |  |  |  |  |  |  |  |  |  |  |
| c.289G > TA | 3 | NP_000050.2: p.Glu97Ter | NGS |  | Nonsense | Pathogenic | 33 | 1 | Bakkach J et al. (45) | Morrocon | 2020 |
| c.5116_ 5119delAATA | 11 | NP_000050.2: p.Asn1706Leufs | NGS |  | Frameshift | Pathogenic | 33 | 1 |  | Morrocon | 2020 |
| c.4090A > G | 11 | NP_000050.2: p.Ile1364Val | NGS |  | Misense | VUS | 33 | 1 |  | Morrocon | 2020 |
| c.6322C > T | 11 | NP_000050.2: p.Arg2108Cys | NGS |  | Misense | Conflicting interpretations of pathogenicity | 33 | 1 |  | Morrocon | 2020 |
|  |  |  |  |  |  |  |  |  |  |  |  |
| c.2254_2257delGACT | 10 | p.Asp752Phefs*19 | NGS | rs80359326 | Frameshif | Pathogenic | 192 | 2 | Abu-Helalah M et al. (46) | Jordan | 2020 |
| c.5042_5043delTG | 11 | p.Val1681Glufs*7 | NGS | rs80359478 | Frameshif | Pathogenic | 192 | 1 |  | Jordan | 2020 |
| c.5351dupA | 11 | p.Asn1784Lysfs*3 | NGS | rs80359508 | Frameshif | Pathogenic | 192 | 1 |  | Jordan | 2020 |
| C.6634_6637delTGTT | 11 | p.Cys2212Leufs*16 | NGS | rs397507871 – | Frameshif | Pathogenic | 192 | 2 |  | Jordan | 2020 |
| c.8696A>G | 1 | p.Gln2899Arg | NGS | N/A | Misense | n/a | 192 | 1 |  | Jordan | 2020 |
|  |  |  |  |  |  |  |  |  |  |  |  |
| exon 5–11 duplication | 5–11 | Absent or disrupted protein product | NGS |  | Large duplication |  | 616 | 8 | Abdel-Razeq H et al. (30) | Jordan | 2023 |
| c.658_659del | 8 | p.Val220Ilefs | NGS |  | Deletion/frameshift |  | 616 | 1 |  | Jordan | 2023 |
| c.1233dup | 10 | p.Pro412Thrfs | NGS |  | Duplication/frameshift |  | 616 | 5 |  | Jordan | 2023 |
| c.1013del | 10 | p.Ala338Metfs | NGS |  | Deletion/frameshift |  | 616 | 1 |  | Jordan | 2023 |
| c.2254_2257del | 11 | p.Asp752Phefs | NGS |  | Deletion/frameshift |  | 616 | 11 |  | Jordan | 2023 |
| c.2254_2257del & c.5351dup | 11 | p.Asp752Phefs & p.Asn1784Lysfs | NGS |  | Deletion/frameshift |  | 616 | 1 |  | Jordan | 2023 |
| c.6685G>T | 11 | p.Glu2229Ter | NGS |  | Nonsense |  | 616 | 3 |  | Jordan | 2023 |
| c.6486_6489del | 11 | p.Lys2162Asnfs | NGS |  | Deletion/frameshift |  | 616 | 1 |  | Jordan | 2023 |
| c.4222_4223del | 11 | p.Gln1408Argfs | NGS |  | Deletion/frameshift |  | 616 | 1 |  | Jordan | 2023 |
| c.6627_6634del | 11 | p.Ile2209Metfs | NGS |  | Deletion/frameshift |  | 616 | 1 |  | Jordan | 2023 |
| c.2677C>T | 11 | p.Gln893Ter | NGS |  | Nonsense |  | 616 | 1 |  | Jordan | 2023 |
| c.6193C>T | 11 | p.Gln2065Ter | NGS |  | Nonsense |  | 616 | 1 |  | Jordan | 2023 |
| c.2808_2811del | 11 | p.Ala938Profs | NGS |  | Deletion/frameshift |  | 616 | 1 |  | Jordan | 2023 |
| c.4936_4939del | 11 | p.Glu1646Glnfs | NGS |  | Deletion/frameshift |  | 616 | 1 |  | Jordan | 2023 |
| c.5732_5732del | 11 | p.Leu1918Argfs | NGS |  | Deletion/frameshift |  | 616 | 1 |  | Jordan | 2023 |
| c.6445_6446del | 11 | p.Ile2149Ter | NGS |  | Nonsense |  | 616 | 1 |  | Jordan | 2023 |
| c.6022A>T | 11 | p.Tyr2008Ter | NGS |  | Nonsense |  | 616 | 1 |  | Jordan | 2023 |
| c.7007G>A | 13 | p.Arg2336His | NGS |  | Missense |  | 616 | 1 |  | Jordan | 2023 |
| c.8140C>T | 16 | p.Gln2714Ter | NGS |  | Nonsense |  | 616 | 1 |  | Jordan | 2023 |
| c.8876C>G | 22 | p.Gln2960Ter | NGS |  | Nonsense |  | 616 | 1 |  | Jordan | 2023 |
| c.8760T>G | 23 | p.Tyr2920Ter | NGS |  | Nonsense |  | 616 | 1 |  | Jordan | 2023 |
| c.9257-1G>A / IVS24-1G>A | Intron 24 | Splice acceptor | NGS |  | Splice site |  | 616 | 3 |  | Jordan | 2023 |
|  |  |  |  |  |  |  |  |  |  |  |  |
| c.51_52del | 2 | p.Arg18Leufs*12 | NGS |  | Frameshift | Likely pathogenic/pathogenic | 443 | 2 | Solano AR et al. (47) | Argentina | 2024 |
| c.156_157ins(Alu) | 3 |  | NGS |  | Insertion | Likely pathogenic/pathogenic | 443 | 1 |  | Argentina | 2024 |
| c.214A>C | 3 | p.Asn72His | NGS |  | Missense | Likely pathogenic/pathogenic | 443 | 1 |  | Argentina | 2024 |
| c.516+3A>G | 6i |  | NGS |  | Splice region | Likely pathogenic/pathogenic | 443 | 1 |  | Argentina | 2024 |
| c.1337T>A | 10 | p.Leu446* | NGS |  | Nonsense | Likely pathogenic/pathogenic | 443 | 1 |  | Argentina | 2024 |
| c.1909+1G>A | 10i |  | NGS |  | Splice donor | Likely pathogenic/pathogenic | 443 | 1 |  | Argentina | 2024 |
| c.2657del | 11 | p.Asn886Metfs*9 | NGS |  | Frameshift | Likely pathogenic/pathogenic | 443 | 1 |  | Argentina | 2024 |
| c.2808_2811del | 11 | p.Ala938Profs*21 | NGS |  | Frameshift | Likely pathogenic/pathogenic | 443 | 4 |  | Argentina | 2024 |
| c.2830A>T | 11 | p.Lys944* | NGS |  | Nonsense | Likely pathogenic/pathogenic | 443 | 1 |  | Argentina | 2024 |
| c.3744_3747del | 11 | p.Ser1248Argfs*10 | NGS |  | Frameshift | Likely pathogenic/pathogenic | 443 | 1 |  | Argentina | 2024 |
| c.3847_3828del | 11 | p.Val1283Lysfs*2 | NGS |  | Frameshift | Likely pathogenic/pathogenic | 443 | 1 |  | Argentina | 2024 |
| c.4277del | 11 | p.Thr1426Asnfs*22 | NGS |  | Frameshift | Likely pathogenic/pathogenic | 443 | 1 |  | Argentina | 2024 |
| c.4928T>C | 11 | p.Val1643Ala | NGS |  | Missense | Likely pathogenic/pathogenic | 443 | 1 |  | Argentina | 2024 |
| c.5351dup | 11 | p.Asn1784Lysfs*3 | NGS |  | Frameshift | Likely pathogenic/pathogenic | 443 | 1 |  | Argentina | 2024 |
| c.5682C>G | 11 | p.Tyr1894* | NGS |  | Nonsense | Likely pathogenic/pathogenic | 443 | 1 |  | Argentina | 2024 |
| c.5946del | 11 | p.Ser1982Argfs*22 | NGS |  | Frameshift | Likely pathogenic/pathogenic | 443 | 2 |  | Argentina | 2024 |
| c.6024dup | 11 | p.Gln2009Alafs*9 | NGS |  | Frameshift | Likely pathogenic/pathogenic | 443 | 3 |  | Argentina | 2024 |
| c.6275_6276del | 11 | p.Leu2092Profs*7 | NGS |  | Frameshift | Likely pathogenic/pathogenic | 443 | 1 |  | Argentina | 2024 |
| c.6405_6409del | 11 | p.Asn2135Lysfs*3 | NGS |  | Frameshift | Likely pathogenic/pathogenic | 443 | 1 |  | Argentina | 2024 |
| c.6596del | 11 | p.Thr2199Ilefs*7 | NGS |  | Frameshift | Likely pathogenic/pathogenic | 443 | 1 |  | Argentina | 2024 |
| c.7480C>T | 15 | p.Arg2494* | NGS |  | Nonsense | Likely pathogenic/pathogenic | 443 | 1 |  | Argentina | 2024 |
| c.7985C>T | 18 | p.Thr2662Met | NGS |  | Missense | Likely pathogenic/pathogenic | 443 | 1 |  | Argentina | 2024 |
| c.8351G>A | 19 | p.Arg2784Gln | NGS |  | Missense | Likely pathogenic/pathogenic | 443 | 1 |  | Argentina | 2024 |
| c.8487+1G>A | 19i |  | NGS |  | Splice donor | Likely pathogenic/pathogenic | 443 | 1 |  | Argentina | 2024 |
| c.8754+4A>G | 21i |  | NGS |  | Splice region | Likely pathogenic/pathogenic | 443 | 1 |  | Argentina | 2024 |
| c.8755-1G>A | 21i |  | NGS |  | Splice acceptor | Likely pathogenic/pathogenic | 443 | 1 |  | Argentina | 2024 |
| c.8942A>G | 22 | p.Glu2981Gly | NGS |  | Missense | Likely pathogenic/pathogenic | 443 | 1 |  | Argentina | 2024 |
| c.9481A>T | 25 | p.Lys3161* | NGS |  | Nonsense | Likely pathogenic/pathogenic | 443 | 1 |  | Argentina | 2024 |
|  |  |  |  |  |  |  |  |  |  |  |  |
| c.8191C>T |  | p.Gln2731Ter | NGS |  | Nonsense | Pathogenic | 75 | 1 | Szczerba E et al. (48) | Polish | 2022 |
| c.9079dupA |  | p.Thr3033AsnfsTer11 | NGS |  | Frameshift | Pathogenic | 75 | 1 |  | Polish | 2022 |
| c.5645C>A |  | p.Ser1882Ter | NGS |  | Nonsense | Pathogenic | 75 | 1 |  | Polish | 2022 |
| c.7758G>A |  | p.Trp2586Ter | NGS |  | Nonsense | Pathogenic | 75 | 1 |  | Polish | 2022 |
|  |  |  |  |  |  |  |  |  |  |  |  |
| c.7480C>T |  | p.Arg2494* | NGS |  | Nonsense |  | 4215 | 40 | Bang YJ et al. (49) | Korean | 2021 |
| c.1399A>T |  | p.Lys467* | NGS |  | Nonsense |  | 4215 | 23 |  | Korean | 2021 |
| c.3744_3747del |  | p.Ser1248Argfs*10 | NGS |  | Frameshift |  | 4215 | 14 |  | Korean | 2021 |
| c.5576_5579del |  | p.Ile1859Lysfs*3 | NGS |  | Frameshift |  | 4215 | 10 |  | Korean | 2021 |
| c.6724_6725del |  | p.Asp2242Phefs*2 | NGS |  | Frameshift |  | 4215 | 6 |  | Korean | 2021 |
| c.9076C>T |  |  | NGS |  | Nonsense |  | 4215 |  |  | Korean | 2021 |
| c.8991T>G |  |  | NGS |  | Missense |  | 4215 | 6 |  | Korean | 2021 |
|  |  |  |  |  |  |  |  |  |  |  |  |
| c.428dup |  | p.(Val144Cysfs) | NGS |  | Duplication | Pathogenic | 376 | 3 | Stella S et al. (50) | Sicily | 2023 |
| c.631G>A |  | p.(Val211Ile) | NGS |  | SNV | Pathogenic | 376 | 1 |  | Sicily | 2023 |
| c.7008–2A>T |  | / | NGS |  | SNV | Pathogenic | 376 |  |  | Sicily | 2023 |
| c.5073_5074insA |  | p.(Trp1692Metfs) | NGS |  | Duplication | Pathogenic | 376 | 1 |  | Sicily | 2023 |
| c.5603_5606delACAG |  | p.(Asp1868Valfs) | NGS |  | Deletion | Pathogenic | 376 | 1 |  | Sicily | 2023 |
| c.5851_5854delAGTT |  | p.(Ser1951TrpfsTer) | NGS |  | Deletion | Pathogenic | 376 | 2 |  | Sicily | 2023 |
| c.6082_6086delGAAGA |  | p.(Glu2028Lysfs) | NGS |  | Deletion | Pathogenic | 376 | 1 |  | Sicily | 2023 |
| c.6486_6489delACAA |  | p.(Lys2162Asnfs) | NGS |  | Deletion | Pathogenic | 376 | 1 |  | Sicily | 2023 |
| c.8331+2T>C |  | / | NGS |  | SNV | Pathogenic | 376 | 1 |  | Sicily | 2023 |
| c.8487+1G>A |  | / | NGS |  | SNV | Pathogenic | 376 | 3 |  | Sicily | 2023 |
| c.8754+4A>G |  | / | NGS |  | SNV | Pathogenic | 376 | 1 |  | Sicily | 2023 |
| c.9004G>A |  | p.(Glu3002Lys) | NGS |  | SNV | Pathogenic | 376 | 1 |  | Sicily | 2023 |
| c.9026_9030delATCAT |  | p.(Tyr3009Serfs) | NGS |  | Deletion | Pathogenic | 376 | 1 |  | Sicily | 2023 |
| c.9455_9456delAG |  | p.(Glu3152Glyfs) | NGS |  | Deletion | Pathogenic | 376 | 1 |  | Sicily | 2023 |
|  |  |  |  |  |  |  |  |  |  |  |  |
| c.256del | 3 | p.Leu86Ter | NGS | Unreported | frameshift | Pathogenic | 137 | 1 | Brahim SM et al. (51) | Mauritania | 2023 |
| c.2892_2893insC | 3 | p.Met965HisfsTer17 | NGS | Unreported | frameshift | Pathogenic | 137 | 1 |  | Mauritania | 2023 |
| c.6280_6286del | 3 | p.Tyr2094LeufsTer23 | NGS | rs80359572 | frameshift | Pathogenic | 137 | 2 |  | Mauritania | 2023 |
| c.7234_7235insG | 3 | p.Thr2412SerfsTer2 | NGS | rs397507906 | frameshift | Pathogenic | 137 | 4 |  | Mauritania | 2023 |
| c.8910G>A | 3 | p.Trp2970Ter | NGS | rs886040799 | stop_gained | Pathogenic | 137 | 1 |  | Mauritania | 2023 |
| c.8969G>A | 3 | p.Trp2990Ter | NGS | rs80359148 | stop_gained | Pathogenic | 137 | 1 |  | Mauritania | 2023 |
| c.5800C>T | 11 | p.Gln1934Ter | NGS | rs886040610 | stop_gained | Pathogenic | 137 | 1 |  | Mauritania | 2023 |
| c.6125A>G | 11 | p.Gln2042Arg | NGS | rs80358852 | Missense | Conficting of pathogenicity | 137 | 1 |  | Mauritania | 2023 |
|  |  |  |  |  |  |  |  |  |  |  |  |
| c.6591_6592del | 11 | p.Glu2198fs | NGS +Sanger Sequencing+MLPA | rs80359605 | Frameshift | Pathogenic | 100 | 1 | Rweyemamu LP et al.(52) | Tanzanian | 2022 |
|  |  |  |  |  |  |  |  |  |  |  |  |
| c.1310_1313 DelAAGA |  |  | NGS+Sanger |  | Frameshift | Pathogenic | 184 | 12 | Melki R et al. (53) | Morroco | 2022 |
|  |  |  |  |  |  |  |  |  |  |  |  |
| c.7878G>A | 17 | p.Trp2626Ter | NGS | rs80359013 |  | Pathogenic | 6 | 5 | Rioki JN et al. (54) | Kenya | 2022 |
| c.9154C>T | 24 | p.Arg3052Trp | NGS | rs45580035 |  | Pathogenic | 6 | 3 |  | Kenya | 2023 |
| c.8243G>A | 18 | p.Gly2748Asp | NGS | rs80359071 |  | Pathogenic | 6 | 5 |  | Kenya | 2024 |
| c.7976G>A | 17 | p.Arg2659Lys | NGS | rs80359027 |  | Pathogenic | 6 | 5 |  | Kenya | 2025 |
| c.8165C>G | 18 | p.Thr2722Arg | NGS | rs80359062 |  | Pathogenic | 6 | 5 |  | Kenya | 2026 |
| c.8167G>C | 18 | p.Asp2723His | NGS | rs41293511 |  | Pathogenic | 6 | 5 |  | Kenya | 2027 |
| c.8168A>T | 18 | p.Asp2723Val | NGS | rs41293513 |  | Pathogenic/likely pathogenic | 6 | 5 |  | Kenya | 2028 |
| c.475+1G>T | 5 |  | NGS | rs81002797 |  | Pathogenic | 6 | 5 |  | Kenya | 2029 |
| c.476-2A>G | 6 |  | NGS | rs81002853 |  | Pathogenic | 6 | 5 |  | Kenya | 2030 |
| c.4987-1G>C | 17 |  | NGS | rs730881495 |  | Pathogenic/likely pathogenic | 6 | 5 |  | Kenya | 2031 |
| c.682-2A>G | 9 |  | NGS | rs878853287 |  | Pathogenic | 6 | 5 |  | Kenya | 2032 |
| c.7008-2A>T | 14 |  | NGS | rs81002823 |  | Pathogenic | 6 | 5 |  | Kenya | 2033 |
| c.7617+1G>T | 15 |  | NGS | rs397507922 |  | Pathogenic/likely pathogenic | 6 | 5 |  | Kenya | 2034 |
| c.8487+1G>A | 19 |  | NGS | rs81002798 |  | Pathogenic | 6 | 5 |  | Kenya | 2035 |
| c.8953+1G>A | 22 |  | NGS | rs81002882 |  | Pathogenic/likely pathogenic | 6 | 5 |  | Kenya | 2036 |
| c.7618-1G>A | 16 |  | NGS | rs397507389 |  | Pathogenic | 6 | 5 |  | Kenya | 2037 |
| c.7988A>G | 18 | p.Glu2663Gly | NGS | rs80359031 |  | Uncertain significance | 6 | 5 |  | Kenya | 2038 |
| c.7879A>G | 17 | p.Ile2627Val | NGS | rs80359014 |  | Uncertain significance | 6 | 5 |  | Kenya | 2039 |
|  |  |  |  |  |  |  |  |  |  |  |  |
| c.5164_5165del | 11 | p.Ser1722Tyrfs*4 | NGS | — | Deletion/Frameshift | — | 72 | 7 | Zhang et al.(55) | Chinese Hakka | 2022 |
|  |  |  |  |  |  |  |  |  |  |  |  |
| c.262â€“263delCT |  | c.262â€“263delCT | NGS |  | Deletion | Pathogenic | 2216 | 1 | Yu S et al. (56) | Chinese | 2022 |
| c.433dupG |  | c.433dupG | NGS |  | Duplication | Pathogenic | 2216 | 1 |  | Chinese | 2022 |
| c.439C>T |  | c.439C>T | NGS |  | Substitution | Pathogenic | 2216 | 1 |  | Chinese | 2022 |
| c.470â€“474delAGTCA |  | c.470â€“474delAGTCA | NGS |  | Deletion | Pathogenic | 2216 | 3 |  | Chinese | 2022 |
| c.771â€“775delTCAAA |  | c.771â€“775delTCAAA | NGS |  | Deletion | Pathogenic | 2216 | 1 |  | Chinese | 2022 |
| c.1055dupA |  | c.1055dupA | NGS |  | Duplication | Pathogenic | 2216 | 1 |  | Chinese | 2022 |
| c.1465â€“1471delTCTGGAA |  | c.1465â€“1471delTCTGGAA | NGS |  | Deletion | Pathogenic | 2216 | 1 |  | Chinese | 2022 |
| c.2471T>G |  | c.2471T>G | NGS |  | Substitution | Pathogenic | 2216 | 1 |  | Chinese | 2022 |
| c.2514dupA |  | c.2514dupA | NGS |  | Duplication | Pathogenic | 2216 | 1 |  | Chinese | 2022 |
| c.2657dupA |  | c.2657dupA | NGS |  | Duplication | Pathogenic | 2216 | 1 |  | Chinese | 2022 |
| c.2845delT |  | c.2845delT | NGS |  | Deletion | Pathogenic | 2216 | 4 |  | Chinese | 2022 |
| c.3109C>T |  | c.3109C>T | NGS |  | Substitution | Pathogenic | 2216 | 3 |  | Chinese | 2022 |
| c.3189â€“3192delGTCA |  | c.3189â€“3192delGTCA | NGS |  | Deletion | Pathogenic | 2216 | 2 |  | Chinese | 2022 |
| c.3256dupA |  | c.3256dupA | NGS |  | Duplication | Pathogenic | 2216 | 2 |  | Chinese | 2022 |
| c.3365delG |  | c.3365delG | NGS |  | Deletion | Pathogenic | 2216 | 2 |  | Chinese | 2022 |
| c.3599â€“3600delGT |  | c.3599â€“3600delGT | NGS |  | Deletion | Pathogenic | 2216 | 1 |  | Chinese | 2022 |
| c.3865â€“3868delAAAT |  | c.3865â€“3868delAAAT | NGS |  | Deletion | Pathogenic | 2216 | 1 |  | Chinese | 2022 |
| c.5351dupA |  | c.5351dupA | NGS |  | Duplication | Pathogenic | 2216 | 1 |  | Chinese | 2022 |
| c.5576â€“5579delTTAA |  | c.5576â€“5579delTTAA | NGS |  | Deletion | Pathogenic | 2216 | 1 |  | Chinese | 2022 |
| c.5682C>G |  | c.5682C>G | NGS |  | Substitution | Pathogenic | 2216 | 5 |  | Chinese | 2022 |
| c.6205â€“6206delTT |  | c.6205â€“6206delTT | NGS |  | Deletion | Pathogenic | 2216 | 1 |  | Chinese | 2022 |
| c.6302dupA |  | c.6302dupA | NGS |  | Duplication | Pathogenic | 2216 | 1 |  | Chinese | 2022 |
| c.6405â€“6409delCTTAA |  | c.6405â€“6409delCTTAA | NGS |  | Deletion | Pathogenic | 2216 | 1 |  | Chinese | 2022 |
| c.6547delG |  | c.6547delG | NGS |  | Deletion | Pathogenic | 2216 | 2 |  | Chinese | 2022 |
| c.6486â€“6489delACAA |  | c.6486â€“6489delACAA | NGS |  | Deletion | Pathogenic | 2216 | 2 |  | Chinese | 2022 |
| c.7090G>T |  | c.7090G>T | NGS |  | Substitution | Pathogenic | 2216 | 1 |  | Chinese | 2022 |
| c.7142delC |  | c.7142delC | NGS |  | Deletion | Pathogenic | 2216 | 1 |  | Chinese | 2022 |
| c.7409dupT |  | c.7409dupT | NGS |  | Duplication | Pathogenic | 2216 | 7 |  | Chinese | 2022 |
| c.7673â€“7674delAG |  | c.7673â€“7674delAG | NGS |  | Deletion | Pathogenic | 2216 | 1 |  | Chinese | 2022 |
| c.8234dupT |  | c.8234dupT | NGS |  | Duplication | Pathogenic | 2216 | 1 |  | Chinese | 2022 |
| c.8377G>T |  | c.8377G>T | NGS |  | Substitution | Pathogenic | 2216 | 2 |  | Chinese | 2022 |
| c.8584dupC |  | c.8584dupC | NGS |  | Duplication | Pathogenic | 2216 | 1 |  | Chinese | 2022 |
| c.8687â€“8690delGTGC |  | c.8687-8690delGTGC | NGS |  | Deletion | Pathogenic | 2216 | 1 |  | Chinese | 2022 |
| c.10150C>T |  | c.10150C>T | NGS |  | Substitution | Pathogenic | 2216 | 1 |  | Chinese | 2022 |
|  |  |  |  |  |  |  |  |  |  |  |  |
| c.100G>T | E3 | c.100G>T | NGS | rs80358391 | Nonsense | Pathogenic | 70 | 1 | Hassan AN et al. (57) | Iraqi Kurdish | 2024 |
| c.1813delA | E1 | c.1813delA | NGS | rs80359306 | Frameshift | Pathogenic | 70 | 1 |  | Iraqi Kurdish | 2024 |
| c.1909+12delT | Intronic | c.1909+12delT | NGS | rs276174816 | Frameshift | Conflicting interpretations of pathogenicity | 70 | 44 |  | Iraqi Kurdish | 2024 |
| c.3318C>G | E11 | c.3318C>G | NGS | rs1298550035 | Missense | Conflicting interpretations of pathogenicity | 70 | 1 |  | Iraqi Kurdish | 2024 |
| c.6966G>T | E13 | c.6966G>T | NGS | rs80358924 | Missense | Uncertain significance | 70 | 1 |  | Iraqi Kurdish | 2024 |
|  |  |  |  |  |  |  |  |  |  |  |  |
| c.1819A>T | 10 | p.Lys607Ter | NGS |  | Nonsense | Pathogenic | 1336 | 1 | Al Amri WS et al. (58) | Omani (Middle Eastern) | 2022 |
| Exon 3 deletion3 | 3 |  | NGS |  | Deletion |  | 1336 | 1 |  | Omani (Middle Eastern) | 2022 |
| c.5290_5291delTC | 11 | p.Ser1764fs | NGS |  | Deletion |  | 1336 | 1 |  | Omani (Middle Eastern) | 2022 |
| c.5705delA* | 11 | p.Asp1902fs | NGS |  | Deletion |  | 1336 | 1 |  | Omani (Middle Eastern) | 2022 |
| c.7673_7674delAG | 16 | p.Glu2558fs | NGS |  | Deletion |  | 1336 | 1 |  | Omani (Middle Eastern) | 2022 |
| c.7679_7680delTT | 16 | p.Phe2560fs | NGS |  | Deletion |  | 1336 | 1 |  | Omani (Middle Eastern) | 2022 |
| c.9018C>A2 | 23 | p.Tyr3006Ter | NGS |  | Nonsense |  | 1336 | 1 |  | Omani (Middle Eastern) | 2022 |
| c.9382C>T1 | 25 | p.Arg3128Ter | NGS |  | Nonsense |  | 1336 | 1 |  | Omani (Middle Eastern) | 2022 |
| c.2588dupA1 | 11 | p.Asn863fs | NGS |  | Nonsense |  | 1336 | 1 |  | Omani (Middle Eastern) | 2022 |
| c.644_646delAAG | 8 | p.Glu215del | NGS |  | Deletion | VUS | 1336 | 1 |  | Omani (Middle Eastern) | 2022 |
| c.161dupA* | 3 | p.Asn54fs | NGS |  | Nonsense |  | 1336 | 1 |  | Omani (Middle Eastern) | 2022 |
| c.1574C>G | 10 | p.Thr525Ser | NGS |  | Nonsense |  | 1336 | 1 |  | Omani (Middle Eastern) | 2022 |
| c.1694C>T | 10 | p.Ala565Val | NGS |  | Missense |  | 1336 | 1 |  | Omani (Middle Eastern) | 2022 |
| c.4045A> | 11 | p.Ile1349Val | NGS |  | Missense |  | 1336 | 1 |  | Omani (Middle Eastern) | 2022 |
| c.800G>A | 10 | p.Gly267Glu | NGS |  | Missense |  | 1336 | 1 |  | Omani (Middle Eastern) | 2022 |
| c.9586A>G | 26 | p.Lys3196Glu | NGS |  | Missense |  | 1336 | 1 |  | Omani (Middle Eastern) | 2022 |
| c.2808_2811delACAA | 11 | p.Ala938fs | NGS |  | Deletion | Pathogenic | 1336 | 1 |  | Omani (Middle Eastern) | 2022 |
| c.3195_3198delTAAT | 11 | p.Asn1066fs | NGS |  | Deletion |  | 1336 | 1 |  | Omani (Middle Eastern) | 2022 |
| c.4718delG* | 11 | p.Cys1573fs | NGS |  | Deletion |  | 1336 | 1 |  | Omani (Middle Eastern) | 2022 |
| c.1423G>T | 10 | p.Glu475Ter | NGS |  | Nonsense |  | 1336 | 1 |  | Omani (Middle Eastern) | 2022 |
| c.1794_1798delATCTT | 10 | p.Ser599Ter | NGS |  | Nonsense |  | 1336 | 1 |  | Omani (Middle Eastern) | 2022 |
| c.8530G>A | 20 | p.Glu2844Lys | NGS |  | Nonsense | VUS | 1336 | 1 |  | Omani (Middle Eastern) | 2022 |
| c.8633-6T>A | â€” | p.[?] | NGS |  | Splice site |  | 1336 | 1 |  | Omani (Middle Eastern) | 2022 |
|  |  |  |  |  |  |  |  |  |  |  |  |
| c.9246dup |  | p.(Lys3083GlufsTer28) | NGS |  | Frameshift | Pathogenic | 307 | 1 | Rojas LXR et al. (59) | Colombian | 2022 |
| c.6275_6276del |  | p.(Leu2092ProfsTer7) | NGS |  | Frameshift | Pathogenic | 307 | 5 |  | Colombian | 2022 |
| c.7673_7674del |  | p.(Glu2558ValfsTer7) | NGS |  | Frameshift | Pathogenic | 307 | 1 |  | Colombian | 2022 |
| c.1796_1800del |  | p.(Ser599Ter) | NGS |  | Nonsense | Pathogenic | 307 | 1 |  | Colombian | 2022 |
|  |  |  |  |  |  |  |  |  |  |  |  |
| c.9976A>T | 27 | p.Lys3326Ter |  | rs11571833 | SNP | Nonsense | 79 | 1 | Shah ND et al. (31) | Indian | 2018 |
| c.484_484delG | 6 | p.Pro163fs |  | Novel | Deletion | Frameshift | 79 | 2 |  | Indian | 2018 |
| c.2971A>G | 11 | p.Asn991Asp |  | rs1799944 | SNP | Missense | 79 | 7 |  | Indian | 2018 |
| c.4779A>C | 11 | p.Glu1593Asp |  | rs80358703 | SNP | Missense | 79 | 2 |  | Indian | 2018 |
| c.865A>C | 10 | p.Asn289His |  | rs766173 | SNP | Missense | 79 | 9 |  | Indian | 2018 |
| c.5744C>T | 11 | p.Thr1915Met |  | - | SNP | Missense | 79 | 3 |  | Indian | 2018 |
| c.7397T>C | 14 | p.Val2466Ala |  | - | SNP | Missense | 79 | 6 |  | Indian | 2018 |
| c.1114A>C | 11 | p.Asn372His |  | rs144848 | SNP | Missense | 79 | 4 |  | Indian | 2018 |
| c.8117A>G | 18 | p.Asn2706Ser |  | rs80359055 | SNP | Missense | 79 | 1 |  | Indian | 2018 |
| c.9380G>A | 25 | p.Trp3127Ter |  | rs80359211 | Nonsense | Nonsense | 79 | 1 |  | Indian | 2018 |
| c.125A>G | 3 | p.Try42Cys |  | rs4987046 | SNP | Missense | 79 | 1 |  | Indian | 2018 |
| c.3167_3170delAAAA | 11 | p.Gln1056 |  | rs80359372 | Deletion | Frameshift | 79 | 1 |  | Indian | 2018 |
| c.4258G>T | 11 | p.Asp1420Tyr |  | rs28897727 | SNP | Missense | 79 | 1 |  | Indian | 2018 |
| c.943T>A | 10 | p.Cys315Ser |  | rs79483201 | SNP | Missense | 79 | 1 |  | Indian | 2018 |
| c.2169_2170insA | 10 | p.Val726fs |  | Novel | Insertion | Frameshift | 79 | 1 |  | Indian | 2018 |
