# Supplementary material for: Global prevalence and ethnic variation of pathogenic BRCA1/2 variants in breast cancer: a systematic review and meta-analysis
Source: J Transl Med. 2026 Mar 12;24:555. doi: 10.1186/s12967-026-07997-3 (PMC13097826; doi:10.1186/s12967-026-07997-3)
Supplement: Supplementary file 6 — Supplementary Material 6 [file 12967_2026_7997_MOESM6_ESM.docx]

**Supplementary Table S4. Characteristics of Included Studies and Distribution of Pathogenic (PLP) Variants by Ethnicity**.

| ***Broad Ethnic Classification*** | ***Number of Studies*** | ***Total Cases Analyzed (N)*** | ***BRCA1 PLP Variants (n)*** | ***BRCA2 PLP Variants (n)*** | ***Total PLP Variants (n)*** |
| --- | --- | --- | --- | --- | --- |
| Chinese | 7 | 26,571 | 31 | 68 | 99 |
| Middle Eastern / North African | 14 | 4,144 | 50 | 40 | 90 |
| Asian | 6 | 7,874 | 4 | 0 | 4 |
| Hispanic / Latino | 6 | 2,246 | 81 | 49 | 130 |
| European | 4 | 1,206 | 26 | 23 | 49 |
| Black / African Descent | 5 | 614 | 32 | 19 | 51 |
| Ashkenazi Jewish | 1 | 3 | 3 | 0 | 3 |
| Other / Unclassified | 2 | 166 | 1 | 0 | 1 |
| TOTAL | 45 | 42,824 | 228 | 199 | 427* |

**Abbreviations:** PLP, Pathogenic/Likely Pathogenic; *N*, total number of patients; *n*, number of unique variants.

*The column sum (427) represents the sum of unique variants identified *within* each ethnic group. This differs slightly from the global deduplicated count (402) used in Figure 6 because some specific variants (e.g., *BRCA1* c.68_69delAG) were identified in multiple ethnic groups.
