# Supplementary material for: Global prevalence and ethnic variation of pathogenic BRCA1/2 variants in breast cancer: a systematic review and meta-analysis
Source: J Transl Med. 2026 Mar 12;24:555. doi: 10.1186/s12967-026-07997-3 (PMC13097826; doi:10.1186/s12967-026-07997-3)
Supplement: Supplementary file 7 — Supplementary Material 7 [file 12967_2026_7997_MOESM7_ESM.docx]

**Supplementary Table S5.1. List of BRCA1 filtered SNPs in the Asian Population**

| **Variant** | **Exon** | **Protein Change** | **Detection Method** | **Db_Snp150** | **Mutation Type** | **Variant Impact** | **Cases Tested** | **Carriers** | **Reference#** | **Prevalence** | **Prevalence**  **Category** |
| --- | --- | --- | --- | --- | --- | --- | --- | --- | --- | --- | --- |
| c.154C>T | 5 | p.Leu52Phe | Direct sequencing | rs80357084 | M | Unclassified | 328 | 3 | Yoon KA et al. (23) | 0.009 | Low (<2%) |
| c.2566T>C | 11 | p.Tyr856His | Direct sequencing | rs80356892 | M | Unclassified | 328 | 19 |  | 0.058 | Moderate (5–29%) |
| c.3448C>T | 11 | p.Pro1150Ser | Direct sequencing | rs80357272 | M | Unclassified | 328 | 5 |  | 0.015 | Low (<2%) |
| c.4883T>C | 16 | p.Met1628Thr | Direct sequencing | rs4986854 | M | Unclassified | 328 | 8 |  | 0.024 | Low-Moderate (2–4%) |
| c.5339T>C | 22 | p.Leu1780Pro | Direct sequencing | rs80357474 | M | Potentially Pathogenic | 328 | 4 |  | 0.012 | Low (<2%) |
| c.390C>A |  | p.Tyr130Ter | NGS | - | N | High | 2720 | 1 | Hur JY et al. (44) | 0.000 | Low (<2%) |
| c.5445G>A |  | p.Trp1815Ter | NGS | - | N | High | 2720 | 1 |  | 0.000 | Low (<2%) |
| c.5080G>T |  | p.Glu1694Ter | NGS | - | N | High | 2720 | 1 |  | 0.000 | Low (<2%) |
| c.5339T>C |  | p.Leu1780Pro | NGS | -- | M |  | 4215 | 16 | Bang YJ et al. (49)  Bang YJ et al. (49) | 0.004 | Low (<2%) |
| c.5080G>T |  | p.Glu1694Ter | NGS | - | M |  | 4215 | 7 |  | 0.002 | Low (<2%) |
| c.53T>C | 2 | p.Met18Thr | NGS | rs80356929 | M | Likely pathogenic | 137 | 1 | Brahim SM et al. (51) | 0.007 | Low (<2%) |
| c.122A>T | 3 | p.His41Leu | NGS | rs80357276 | M | Likely pathogenic | 137 | 1 |  | 0.007 | Low (<2%) |
| c.131G>C | 4 | p.Cys44Ser | NGS | - | M | Pathogenic | 137 | 1 |  | 0.007 | Low (<2%) |
| c.3119G>A | 10 | p.Ser1040Asn |  | rs4986852 | M |  | 79 | 2 | Shah ND et al. (31) | 0.025 | Low-Moderate (2–4%) |
| c.2412G>C | 11 | p.Gln804His |  | rs55746541 | M |  | 79 | 1 |  | 0.013 | Low (<2%) |
| c.5019G>A | 16 | p.Met1673Ile |  | rs1799967 | M |  | 79 | 2 |  | 0.025 | Low-Moderate (2–4%) |
| c.3113A>G | 10 | p.Glu1038Gly |  | rs16941 | M |  | 79 | 7 |  | 0.089 | Moderate (5–29%) |
| c.2077G>A | 9 | p.Asp693Asn |  | rs4986850 | M |  | 79 | 2 |  | 0.025 | Low-Moderate (2–4%) |
| c.2612C>T | 11 | p.Pro871Leu |  | rs799917 | M |  | 79 | 6 |  | 0.076 | Moderate (5–29%) |
| c.2521C>T | 11 | p.Arg841Try |  | rs1800709 | M |  | 79 | 1 |  | 0.013 | Low (<2%) |
| c.3607C>T |  | p.Arg1203Ter | Targeted Sequencing | rs62625308 | N | pathogenic | 467 | 1 | Yang XR et al. (26) | 0.002 | Low (<2%) |
| c.211C>G | - | p.Pro71Ala | Targeted Sequencing | rs80357382 | M | Pathogenic | 467 | 1 |  | 0.002 | Low (<2%) |
| c.182G>A | - | p.Cys61Tyr | Targeted Sequencing | rs80357093 | M | Pathogenic | 467 | 1 |  | 0.002 | Low (<2%) |
| c.110C>A | - | p.Thr37Lys | Targeted Sequencing | rs80356880 | M | Pathogenic | 467 | 1 |  | 0.002 | Low (<2%) |

**Supplementary Table S5.2. List of BRCA1 filtered SNPs in the Chinese Population**

| **Variant** | **Exon** | **Protein Change** | **Detection Method** | **Db_Snp150** | **Mutation**  **Type** | **Variant Impact** | **Cases Tested** | **Carriers** | **Reference#** | **Prevalence** | **Prevalence category** |
| --- | --- | --- | --- | --- | --- | --- | --- | --- | --- | --- | --- |
| c.212G>A | - | p.R71K | NGS | - | M | Pathogenic | 71 | 1 | Fang M et al.(28) | 0.014 | Low (<2%) |
| c.3626T>G | - | p.L1209X | NGS | - | N | Pathogenic | 71 | 1 |  | 0.014 | Low (<2%) |
| c.5510G>A | 24 | p.Trp1837Ter | NGS + Sanger | - | N | Pathogenic | 595 | 1 | Liang Y et al. (29) | 0.002 | Low (<2%) |
| c.3352C>T | 11 | p.Gln1118Ter | NGS + Sanger | - | N | Pathogenic | 595 | 1 |  | 0.002 | Low (<2%) |
| c.376C>T | 7 | p.Gln126Ter | NGS + Sanger | - | N | Pathogenic | 595 | 1 |  | 0.002 | Low (<2%) |
| c.5353C>T | 22 | p.Gln1785Ter | NGS + Sanger | - | N | Pathogenic | 595 | 1 |  | 0.002 | Low (<2%) |
| c.3472G>T | 11 | p.Glu1158Ter | NGS + Sanger | - | N | Pathogenic | 595 | 1 |  | 0.002 | Low (<2%) |
| c.1012A>T | 11 | p.Lys338Ter | NGS + Sanger | - | N | Pathogenic | 595 | 1 |  | 0.002 | Low (<2%) |
| c.4222C>T | 13 | p.Gln1408Ter | NGS + Sanger | - | N | Pathogenic | 595 | 1 |  | 0.002 | Low (<2%) |
| c.190T>C |  |  | NGS | - | M | Pathogenic | 82 | 1 | Wang T et al. (33) | 0.012 | Low (<2%) |
| c.4837A>G | 15 |  | NGS | - | M | Drug-sensitive | 82 | 4 |  | 0.049 | Low-Moderate (2–4%) |
| c.2612C>T | 10 |  | NGS | - | M | Drug-sensitive | 82 | 4 |  | 0.049 | Low-Moderate (2–4%) |
| c.988G>A | 10 | p.Asp330Asn | PGM & Miseq |  | M | Uncertain | 54 | 1 | Shen M et al.(36) | 0.019 | Low (<2%) |
| c.1036C>T | 10 | p.Pro346Ser | PGM & Miseq |  | M | Uncertain | 54 | 1 |  | 0.019 | Low (<2%) |
| c.2059C>T | 10 | p.Gln687Ter | PGM & Miseq |  | Stop_gained | Pathogenic | 54 | 1 |  | 0.019 | Low (<2%) |
| c.2566T>C | 10 | p.Tyr856His | PGM & Miseq |  | M |  | 54 | 15 |  | 0.278 | Moderate (5–29%) |
| c.2612C>T | 10 | p.Pro871Leu | PGM & Miseq |  | M |  | 54 | 33 |  | 0.611 | Extremely High (≥50%) |
| c.2623C>T | 10 | p.Pro875Ser | PGM & Miseq |  | M | Uncertain | 54 | 1 |  | 0.019 | Low (<2%) |
| c.3113A>G | 10 | p.Glu1038Gly | PGM & Miseq |  | M |  | 54 | 36 |  | 0.667 | Extremely High (≥50%) |
| c.3548A>G | 10 | p.Lys1183Arg | PGM & Miseq |  | M |  | 54 | 34 |  | 0.630 | Extremely High (≥50%) |
| c.4674A>G | 15 | (p.Leu1558=) | PGM & Miseq |  | Synonymous_variant | Uncertain | 54 | 1 |  | 0.019 | Low (<2%) |
| c.4837A>G | 16 | p.Ser1613Gly | PGM & Miseq |  | M |  | 54 | 32 |  | 0.593 | Extremely High (≥50%) |
| c.446A>C | 7 | p.Glu149Ala | PGM & Miseq |  | M |  | 54 | 1 |  | 0.019 | Low (<2%) |
| c.2635G>T | 11 | p.Glu879* | NGS | — | N |  | 72 | 4 | Zhang et al.(55) | 0.056 | Moderate (5–29%) |
| c.1252G>T |  |  | NGS |  | SNV |  | 2216 | 4 | Yu S et al. (56) | 0.002 | Low (<2%) |
| c.2740G>T |  |  | NGS |  | SNV |  | 2216 | 1 |  | 0.000 | Low (<2%) |
| c.3607C>T |  |  | NGS |  | SNV |  | 2216 | 3 |  | 0.001 | Low (<2%) |
| c.4819G>T |  |  | NGS |  | SNV |  | 2216 | 1 |  | 0.000 | Low (<2%) |
| c.5154G>A |  |  | NGS |  | SNV |  | 2216 | 3 |  | 0.001 | Low (<2%) |
| c.5566C>T |  |  | NGS |  | SNV |  | 2216 | 1 |  | 0.000 | Low (<2%) |

**Supplementary Table S5.3. List of BRCA1 filtered SNPs in the Black or African Descent**

| **Variant** | **Exon** | **Protein Change** | **Detection Method** | **Db_Snp150** | **Mutation Type** | **Variant**  **Impact** | **Cases Tested** | **Carriers** | **Reference#** | **Prevalence** | **Prevalence category** |
| --- | --- | --- | --- | --- | --- | --- | --- | --- | --- | --- | --- |
| c.181T>G | 4 | c.181T>G / p.Cys61Gly | NGS | - | M | Pathogenic | 108 | 1 | Francies FZ et al.(17) | 0.009 | Low (<2%) |
| c.212G>A | 4 | c.212G>A / p.Arg71Lys | NGS | - | M | Pathogenic | 108 | 1 |  | 0.009 | Low (<2%) |
| c.3593T>A | 10 | c.3593T>A / p.Leu1198* | NGS | - | N | Pathogenic | 108 | 1 |  | 0.009 | Low (<2%) |
| c.1155G>A | 10 | c.1155G>A / p.Trp385* | NGS | - | N | Pathogenic | 108 | 1 |  | 0.009 | Low (<2%) |
| c.3G>T | 2 | c.3G>T | MLPA |  | M |  | 369 | 1 | Pal T et al. (20) | 0.003 | Low (<2%) |
| c.182G>A | 5 | c.182G>A | MLPA |  | M |  | 369 | 1 |  | 0.003 | Low (<2%) |
| c.4484G>T | 14 | c.4484G>T | MLPA |  | M |  | 369 | 1 |  | 0.003 | Low (<2%) |
| c.5251C>T | 20 | c.5251C>T | MLPA |  | N |  | 369 | 3 |  | 0.008 | Low (<2%) |
| c.5324T>G | 21 | c.5324T>G | MLPA |  | M |  | 369 | 3 |  | 0.008 | Low (<2%) |
| c.5387C>A | 22 | c.5387C>A | MLPA |  | N |  | 369 | 2 |  | 0.005 | Low (<2%) |
| c.5123C>A | 17 | p.Ala1708Glu | NGS +  Sanger Sequencing +MLPA | rs28897696 | M | Pathogenic | 100 | 1 | Abdel-Razeq H et al. (30) | 0.010 | Low (<2%) |
| c.5513T>A | 24 | p.Val1838Glu | NGS | rs80357107 |  | Pathogenic | 6 | 5 | Rioki JN et al. (54) | 0.833 | Extremely High (≥50%) |
| c.5291T>C | 21 | p.Leu1764Pro | NGS | rs80357281 |  | Pathogenic | 6 | 4 |  | 0.667 | Extremely High (≥50%) |
| c.5297T>G | 21 | p.Ile1766Ser | NGS | rs80357463 |  | Pathogenic | 6 | 2 |  | 0.333 | High (30–49%) |
| c.110C>A | 3 | p.Thr37Lys | NGS | rs80356880 |  | Pathogenic | 6 | 5 |  | 0.833 | Extremely High (≥50%) |
| c.5212G>C | 20 | p.Gly1738Arg | NGS | rs80356937 |  | Pathogenic | 6 | 5 |  | 0.833 | Extremely High (≥50%) |
| c.122A>C | 3 | p.His41Pro | NGS | rs80357276 |  | Pathogenic | 6 | 5 |  | 0.833 | Extremely High (≥50%) |
| c.5117G>A | 18 | p.Gly1706Glu | NGS | rs80356860 |  | Pathogenic | 6 | 5 |  | 0.833 | Extremely High (≥50%) |
| c.5095C>T | 18 | p.Arg1699Trp | NGS | rs55770810 |  | Pathogenic | 6 | 5 |  | 0.833 | Extremely High (≥50%) |
| c.5054C>T | 17 | p.Thr1685Ile | NGS | rs80357043 |  | Pathogenic | 6 | 5 |  | 0.833 | Extremely High (≥50%) |
| c.5053A>G | 17 | p.Thr1685Ala | NGS | rs80356890 |  | Pathogenic | 6 | 5 |  | 0.833 | Extremely High (≥50%) |
| c.181T>C | 4 | p.Cys61Arg | NGS | rs28897672 |  | Pathogenic/likely pathogenic | 6 | 5 |  | 0.833 | Extremely High (≥50%) |
| c.131G>T | 3 | p.Cys44Phe | NGS | rs80357446 |  | Pathogenic | 6 | 5 |  | 0.833 | Extremely High (≥50%) |
| c.115T>A | 3 | p.Cys39Ser | NGS | rs80357164 |  | Pathogenic/likely pathogenic | 6 | 5 |  | 0.833 | Extremely High (≥50%) |
| c.5143A>G | 18 | p.Se1715Arg | NGS | rs80357222 |  | Pathogenic | 6 | 5 |  | 0.833 | Extremely High (≥50%) |
| c.130T>G | 3 | p.Cys44Gly | NGS | rs80357327 |  | Pathogenic | 6 | 5 |  | 0.833 | Extremely High (≥50%) |
| c.5359T>A | 22 | p.Cys1787Ser | NGS | rs80357065 |  | Conflicting interpretations of pathogenicity | 6 | 5 |  | 0.833 | Extremely High (≥50%) |
| c.5324T>C | 21 | p.Met1775Thr | NGS | rs41293463 |  | Uncertain significance | 6 | 5 |  | 0.833 | Extremely High (≥50%) |
| c.5123C>T | 18 | p.Ala1708Val | NGS | rs28897696 |  | Uncertain significance | 6 | 5 |  | 0.833 | Extremely High (≥50%) |
| c.4900A>G | 16 | p.Arg1634Gly | NGS | rs1597830733 |  | Uncertain significance | 6 | 2 |  | 0.333 | High (30–49%) |
| c.694G>A | - | Asp232Asn | NGS | rs55975699 | M | VUS | 31 | 1 | Ricks-Santi L et al. (24) | 0.032 | Low-Moderate (2–4%) |
| c.956A>G | - | Asn319Ser | NGS | rs397507258 | M | VUS | 31 | 1 |  | 0.032 | Low-Moderate (2–4%) |

**Supplementary Table S5.4. List of BRCA1 filtered SNPs in the European Population**

| **Variant** | **Exon** | **Protein Change** | **Detection Method** | **Db_Snp150** | **Mutation Type** | **Variant**  **Impact** | **Cases Tested** | **Carriers** | **Reference#** | **Prevalence** | **Prevalence category** |
| --- | --- | --- | --- | --- | --- | --- | --- | --- | --- | --- | --- |
| c.181T>G | 5 | p.Cys61Gly | Sanger | rs80357474 | M | Pathogenic | 200 | 2 | Dodova RI et al. (16) | 0.010 | Low (<2%) |
| c.4603G>T | 15 | p.Glu1535Ter | Sanger | rs80356898 | N | Pathogenic | 200 | 1 |  | 0.005 | Low (<2%) |
| c.1687C>T |  | p.Gln563Ter | NGS |  | N | Pathogenic | 75 | 1 | Szczerba E et al. (48) | 0.013 | Low (<2%) |
| c.4752C>G |  | p.Tyr1584Ter | NGS |  | N | Pathogenic | 75 | 2 |  | 0.027 | Low-Moderate (2–4%) |
| c.5186C>A |  | p.Ala1729Glu | NGS |  | N | Pathogenic | 75 | 1 |  | 0.013 | Low (<2%) |
| c.5242A>T |  | p.Lys1748Ter | NGS |  | N | Pathogenic | 75 | 1 |  | 0.013 | Low (<2%) |
| c.181T>G |  | p.(Cys61Gly) | NGS |  | SNV | Pathogenic | 376 | 1 | Stella S et al. (50) | 0.003 | Low (<2%) |
| c.1204G>T |  | p.(Glu402Ter) | NGS |  | SNV | Pathogenic | 376 | 1 |  | 0.003 | Low (<2%) |
| c.2536G>T |  | p.(Glu846Ter) | NGS |  | SNV | Pathogenic | 376 | 1 |  | 0.003 | Low (<2%) |
| c.4117G>T |  | p.(Glu1373Ter) | NGS |  | SNV | Pathogenic | 376 | 1 |  | 0.003 | Low (<2%) |
| c.4484G>T |  | p.(Arg1495Met) | NGS |  | SNV | Pathogenic | 376 | 1 |  | 0.003 | Low (<2%) |
| c.5509T>C |  | p.(Trp1837Arg) | NGS |  | SNV | Pathogenic | 376 | 1 |  | 0.003 | Low (<2%) |

**Supplementary Table S5.5. List of BRCA1 filtered SNPs in the Hispanic/Latino Population**

| **Variant** | **Exon** | **Protein Change** | **Detection Method** | **Db_Snp150** | **Mutation Type** | **Variant**  **Impact** | **Cases Tested** | **Carriers** | **Reference#** | **Prevalence** | **Prevalence category** |
| --- | --- | --- | --- | --- | --- | --- | --- | --- | --- | --- | --- |
| c.412C>A |  | p.Leu138Ile | NGS |  | M | Non-synonymous | 201 | 1 | Cortés C et al. (38) | 0.005 | Low (<2%) |
| c.2077G>A |  | p.Asp693Asn | NGS |  | M | Non-synonymous | 201 | 2 |  | 0.010 | Low (<2%) |
| c.2079G>A |  | p.Asp693Asp | NGS |  | S | Synonymous | 201 | 2 |  | 0.010 | Low (<2%) |
| c.2082C>T |  | p.Ser694Ser | NGS |  | S | Synonymous | 201 | 30 |  | 0.149 | Moderate (5–29%) |
| c.2146T>A |  | p.Ser716Arg | NGS |  | M | Non-synonymous | 201 | 2 |  | 0.010 | Low (<2%) |
| c.2311T>C |  | p.Leu771Leu | NGS |  | S | Synonymous | 201 | 13 |  | 0.065 | Moderate (5–29%) |
| c.2368A>G |  | p.Thr790Ala | NGS |  | M | Non-synonymous | 201 | 2 |  | 0.010 | Low (<2%) |
| c.2612C>T |  | p.Pro871Leu | NGS |  | M | Non-synonymous | 201 | 64 |  | 0.318 | High (30–49%) |
| c.2876G>A |  | p.Arg959Lys | NGS |  | M | Non-synonymous | 201 | 2 |  | 0.010 | Low (<2%) |
| c.3083G>A |  | p.Arg1028His | NGS |  | M | Non-synonymous | 201 | 3 |  | 0.015 | Low (<2%) |
| c.3113A>G |  | p.Glu1038Gly | NGS |  | M | Non-synonymous | 201 | 27 |  | 0.134 | Moderate (5–29%) |
| c.3506A>C |  | p.Asp1169Ala | NGS |  | M | Non-synonymous | 201 | 1 |  | 0.005 | Low (<2%) |
| c.3548A>G |  | p.Lys1183Arg | NGS |  | M | Non-synonymous | 201 | 20 |  | 0.100 | Moderate (5–29%) |
| c.3978T>G |  | p.His1326Gln | NGS |  | M | Non-synonymous | 201 | 1 |  | 0.005 | Low (<2%) |
| c.4033G>A |  | p.Glu1345Lys | NGS |  | M | Non-synonymous | 201 | 4 |  | 0.020 | Low (<2%) |
| c.4308T>C |  | p.Ser1436Ser | NGS |  | S | Synonymous | 201 | 8 |  | 0.040 | Low-Moderate (2–4%) |
| c.4837A>G |  | p.Ser1613Gly | NGS |  | M | Non-synonymous | 201 | 17 |  | 0.085 | Moderate (5–29%) |
| c.211A>G |  | p.Arg71Gly | NGS | rs80357382 | M | Pathogenic | 252 | 2 | Millan Catalan O et al.(41) | 0.008 | Low (<2%) |
| c.1960A>T |  | p.Lys654Ter | NGS | rs80357355 | N | Pathogenic | 252 | 2 |  | 0.008 | Low (<2%) |
| c.3598C>T |  | p.Gln1200Ter | NGS | rs62625307 | N | Pathogenic | 252 | 3 |  | 0.012 | Low (<2%) |
| c.4327C>T |  | p.Arg1443Ter | NGS | rs41293455 | N | Pathogenic | 252 | 1 |  | 0.004 | Low (<2%) |
| c.5095C>T |  | p.Arg1699Trp | NGS | rs55770810 | M | Pathogenic | 252 | 1 |  | 0.004 | Low (<2%) |
| c.5123C>A |  | p.Ala1708Glu | NGS | rs28897696 | M | Pathogenic | 252 | 3 |  | 0.012 | Low (<2%) |
| c.81-1G>A | 2i |  | NGS |  |  | Likely pathogenic/pathogenic | 443 | 1 | Solano AR et al. (47) | 0.002 | Low (<2%) |
| c.140G>T | 5 | Cys47Phe | NGS |  |  | Likely pathogenic/pathogenic | 443 | 1 |  | 0.002 | Low (<2%) |
| c.1480C>T | 5 | Cys494Ter | NGS |  | M | Likely pathogenic/pathogenic | 443 | 1 |  | 0.002 | Low (<2%) |
| c.181T>G | 5 | Cys61Gly | NGS |  | M | Likely pathogenic/pathogenic | 443 | 2 |  | 0.005 | Low (<2%) |
| c.191G>A | 5 | Cys64Tyr | NGS |  | M | Likely pathogenic/pathogenic | 443 | 1 |  | 0.002 | Low (<2%) |
| c.190T>C | 5 | Cys64Arg | NGS |  | M | Likely pathogenic/pathogenic | 443 | 1 |  | 0.002 | Low (<2%) |
| c.427G>T | 7 | Glu143 | NGS |  | M | Likely pathogenic/pathogenic | 443 | 1 |  | 0.002 | Low (<2%) |
| c.1687C>T | 11 | p.Gln563* | NGS |  | S | Likely pathogenic/pathogenic | 443 | 1 |  | 0.002 | Low (<2%) |
| c.4042G>T | 11 | p.Gly1348* | NGS |  | N | Likely pathogenic/pathogenic | 443 | 1 |  | 0.002 | Low (<2%) |
| c.4183C>T | 11 | p.Gln1395* | NGS |  | N | Likely pathogenic/pathogenic | 443 | 1 |  | 0.002 | Low (<2%) |
| c.4201C>T | 13 | p.Gln1401* | NGS |  | N | Likely pathogenic/pathogenic | 443 | 1 |  | 0.002 | Low (<2%) |
| c.4484G>T | 14 | p.Arg1495Met | NGS |  | M | Likely pathogenic/pathogenic | 443 | 1 |  | 0.002 | Low (<2%) |
| c.5282T>C | 21 | p.Phe1761Ser | NGS |  | M | Likely pathogenic/pathogenic | 443 | 1 |  | 0.002 | Low (<2%) |
| c.5431C>T | 23 | p.Gln1811* | NGS |  | N | Likely pathogenic/pathogenic | 443 | 1 |  | 0.002 | Low (<2%) |
| c.5445G>A | 23 | p.Trp1815* | NGS |  | N | Likely pathogenic/pathogenic | 443 | 1 |  | 0.002 | Low (<2%) |
| c.213C>G | 5 | R71G | PCR + Sanger | - | M | Pathogenic | 190 | 4 | Villarreal-Garza C et al. (19) | 0.021 | Low-Moderate (2–4%) |
| c.3598C>T | 16 | Q1200X | PCR + Sanger | - | N | Pathogenic | 190 | 1 |  | 0.005 | Low (<2%) |
| c.4327C>T | 13 | R1443X | PCR + Sanger | - | N | Pathogenic | 190 | 4 |  | 0.021 | Low-Moderate (2–4%) |
| c.5123C>A | 18 | A1708E | PCR + Sanger | - | M | Pathogenic | 190 | 1 |  | 0.005 | Low (<2%) |
| c.5503C>T | - | R1835X | Sequencing | - | N | Pathogenic | 853 | 1 | Briceño-Balcázar I et al. (27) | 0.001 | Low (<2%) |

**Supplementary Table S3.6. List of BRCA1 filtered SNPs in the Hispanic/Latino Population**

| **Variant** | **Exon** | **Protein Change** | **Detection Method** | **Db_Snp150** | **Mutation Type** | **Variant**  **Impact** | **Cases Tested** | **Carriers** | **Reference#** | **Prevalence** | **Prevalence category** |
| --- | --- | --- | --- | --- | --- | --- | --- | --- | --- | --- | --- |
| c.3607C>T | - | c.3607C>T / p.R1203X | Sequencing | - | N | Pathogenic | 3 | 1 | Walsh T et al. (25) | 0.333 | High (30–49%) |
| c.4480G>T | - | c.4480G>T / p.E1494X | Sequencing | - | N | Pathogenic | 3 | 1 |  | 0.333 | High (30–49%) |

**Supplementary Table S5.7. List of BRCA1 filtered SNPs in the Middle Eastern/North African Population**

| **Variant** | **Exon** | **Protein Change** | **Detection Method** | **Db_Snp150** | **Mutation Type** | **Variant**  **Impact** | **Cases Tested** | **Carriers** | **Reference#** | **Prevalence** | **Prevalence category** |
| --- | --- | --- | --- | --- | --- | --- | --- | --- | --- | --- | --- |
| c.34C>T | 2 | p.Gln12* | Sanger / NGS | - | N | Pathogenic | 250 | 1 | El Saghir NS et al. (18) | 0.004 | Low (<2%) |
| c.131G>T | 3 | p.Cys44Phe | Sanger / NGS | - | M | Pathogenic | 250 | 2 |  | 0.008 | Low (<2%) |
| c.2158G>T | 11 | p.Glu720* | Sanger / NGS | - | N | Pathogenic | 250 | 1 |  | 0.004 | Low (<2%) |
| c.4524G>A | 15 | p.Trp1508X | Capture/Sanger Sequencing | - | N | Pathogenic | 818 | 1 |  | 0.001 | Low (<2%) |
| c.5251C>T | 20 | p.Arg1751X | Capture/Sanger Sequencing | - | N | Pathogenic | 818 | 5 |  | 0.006 | Low (<2%) |
| c.1066C>T | - | - | NGS/Sanger | - | N | Pathogenic | 310 | 1 |  | 0.003 | Low (<2%) |
| c.4524A>T | - | p.Gly1508Gly | NGS/Sanger | - | Synonymous | Likely benign | 310 | 1 |  | 0.003 | Low (<2%) |
| c.4609C>T | - | p.Gln1537* | NGS/Sanger | - | N | Pathogenic | 310 | 1 |  | 0.003 | Low (<2%) |
| c.4609C>T | - | p.Gln1537* | NGS/Sanger | - | N | Pathogenic | 310 | 1 |  | 0.003 | Low (<2%) |
| c.3113A>G | 11 | p.Glu1038Gly | SSCP + Sequencing | rs16941 | M | Polymorphism | 40 | 13 | Khalili-Tanha G et al.(35) | 0.325 | High (30–49%) |
| c.3119G>A | 11 | p.Ser1040Asn | SSCP + Sequencing | rs4986852 | M | Polymorphism | 40 | 2 |  | 0.050 | Moderate (5–29%) |
| c.3548A>G | 11 | pLys1183Arg | SSCP + Sequencing | rs16942 | M | Polymorphism | 40 | 26 |  | 0.650 | Extremely High (≥50%) |
| c.4308T>C | 13 | Ser1436Ser | SSCP + Sequencing | rs1060915 | Synonymous | Polymorphism | 88 | 28 |  | 0.318 | High (30–49%) |
| c.4837A>G | 16 | Ser1613Gly | SSCP + Sequencing | rs1799966 | M | Polymorphism | 88 | 50 |  | 0.568 | Extremely High (≥50%) |
| c.5186C>A | 18 | p.Ala1729Glu | NGS | rs28897696 | M | Pathogenic | 33 | 1 | Abu-Helalah M et al. (46) | 0.030 | Low-Moderate (2–4%) |
| c.5158C>T | 18 | p.Arg1720Trp | NGS | rs55770810 | M | Pathogenic | 33 | 1 |  | 0.030 | Low-Moderate (2–4%) |
| c.121C>T | 3 | p.His41Tyr | NGS |  | M |  | 616 | 1 | Abdel-Razeq H et al. (30) | 0.002 | Low (<2%) |
| c.2761C>T | 12 | p.Gln921Ter | NGS |  | N |  | 616 | 1 |  | 0.002 | Low (<2%) |
| c.4117G>T | 12 | p.Glu1373Ter | NGS |  | N |  | 616 | 4 |  | 0.006 | Low (<2%) |
| c.4524G>A | 15 | p.Trp1508Ter | NGS |  | N |  | 616 | 1 |  | 0.002 | Low (<2%) |
| c.4117G>A | 12 | p.Ala1708Glu | NGS |  | M |  | 616 | 1 |  | 0.002 | Low (<2%) |
| c.5095C>T | 18 | p.Arg1699Trp | NGS |  | M |  | 616 | 1 |  | 0.002 | Low (<2%) |
| c.5161C>T | 19 | p.Gln1721Ter | NGS |  | N |  | 616 | 2 |  | 0.003 | Low (<2%) |
| c.5309G>T |  | (p.Gly1770Val) | NGS + Sanger |  | M | Pathogenic | 184 | 10 | Melki R et al. (53) | 0.054 | Moderate (5–29%) |
| c.3607C>T | 10 | c.3607C>T | NGS | rs62625308 | SNV/N | Pathogenic | 70 | 1 | Hassan AN et al. (57) | 0.014 | Low (<2%) |
| c.3544C>T | 10 | c.3544C>T | NGS | rs80357296 | SNV/N | Pathogenic | 70 | 1 |  | 0.014 | Low (<2%) |
| c.178C>T | 3 | p.Gln60Ter | NGS |  | N | Pathogenic | 1336 | 1 | Al Amri WS et al. (58) | 0.001 | Low (<2%) |
| c.971G>T | 10 | p.Ser324Ile | NGS |  | M | VUS | 1336 | 1 |  | 0.001 | Low (<2%) |
| c.398G>A | 6 | p.Arg133His | NGS |  | N |  | 1336 | 1 |  | 0.001 | Low (<2%) |
| c.4993G>C | 16 | p.Val665Leu | NGS |  | M |  | 1336 | 1 |  | 0.001 | Low (<2%) |
| c.5423T>C | 22 | p.Val1808Ala | NGS |  | M |  | 1336 | 1 |  | 0.001 | Low (<2%) |
| c.2123C>A | 10 | p.Ser708Tyr | NGS |  | N | VUS | 1336 | 1 |  | 0.001 | Low (<2%) |
| c.4236G>T | - | E1373X | NGS/Sanger | - | N | Deleterious | 100 | 1 | Rweyemamu LP et al.(52) | 0.010 | Low (<2%) |

**Supplementary Table S5.8. List of BRCA1 filtered SNPs in the Unclassified Population**

| **Variant** | **Exon** | **Protein Change** | **Detection Method** | **Db_Snp150** | **Mutation Type** | **Variant**  **Impact** | **Cases Tested** | **Carriers** | **Reference#** | **Prevalence** | **Prevalence category** |
| --- | --- | --- | --- | --- | --- | --- | --- | --- | --- | --- | --- |
| c.3607C>T |  | p.R1203 | NGS |  | N |  | 99 | 2 | Geredeli C et al. (37) | 0.020 | Low-Moderate (2–4%) |
| c.442-34C>T |  | IVS7 -34C>T | NGS |  |  |  | 99 | 1 |  | 0.010 | Low (<2%) |
| c.5444G>A |  | p.W1815 | NGS |  | M |  | 99 | 2 |  | 0.020 | Low-Moderate (2–4%) |
| c.962G>A |  | p.(Trp321Ter) | PCR + Sanger |  |  |  | 522 | 0 | Behl S et al. (43) | 0.000 | Low (<2%) |
| c.4327C>T |  | p.(Arg1443Ter) | PCR + Sanger |  |  |  | 552 | 3 |  | 0.005 | Low (<2%) |
| c.1054G>T |  | p.(Glu352Ter) | PCR + Sanger |  |  |  | 553 | 0 |  | 0.000 | Low (<2%) |
| c.5536C>T |  | p.(Gln1846Ter) | PCR + Sanger |  |  |  | 555 | 0 |  | 0.000 | Low (<2%) |

BRCA2.

**Supplementary Table S5.9. List of BRCA2 filtered SNPs in the Asian Population**

| **Variant** | **Exon** | **Protein Change** | **Detection Method** | **Db_Snp150** | **Mutation Type** | **Variant**  **Impact** | **Cases Tested** | **Carriers** | **Reference#** | **Prevalence** | **Prevalence category** |
| --- | --- | --- | --- | --- | --- | --- | --- | --- | --- | --- | --- |
| c.34C>T | 10 | p.Cys315Ser | Direct sequencing | rs79483201 | Missense | Unclassified | 328 | 3 | Yoon KA et al. (23) | 0.009 | Low (<2%) |
| c.1745T>C | 11 | p.Thr582Pro | Direct sequencing | rs80358457 | Missense | Unclassified | 328 | 6 |  | 0.018 | Low (<2%) |
| c.2350A>G | 11 | p.Met784Val | Direct sequencing | rs11571653 | Missense | Unclassified | 328 | 9 |  | 0.027 | Low-Moderate (2–4%) |
| c.3220A>T | 11 | p.Asp1074Val | Direct sequencing | rs14505603 | Missense | Unclassified | 328 | 2 |  | 0.006 | Low (<2%) |
| c.6020T>G | 11 | p.Val2010Gly | Direct sequencing | - | Missense | Unclassified | 328 | 2 |  | 0.006 | Low (<2%) |
| c.6325G>C | 11 | p.Val2109Leu | Direct sequencing | rs79456940 | Missense | Unclassified | 328 | 3 |  | 0.009 | Low (<2%) |
| c.6351G>T | 11 | p.Ala2151Gly | Direct sequencing | - | Missense | Unclassified | 328 | 3 |  | 0.009 | Low (<2%) |
| c.7522G>C | 15 | p.Gly2508Ser | Direct sequencing | - | Missense | Unclassified | 328 | 2 |  | 0.006 | Low (<2%) |
| c.8187G>T | 18 | p.Lys2729Asn | Direct sequencing | rs80359065 | Missense | Unclassified | 328 | 10 |  | 0.030 | Low-Moderate (2–4%) |
| c.7480C>T |  | p.Arg2494* | NGS |  | Nonsense |  | 4215 | 40 | Bang YJ et al. (49) | 0.009 | Low (<2%) |
| c.1399A>T |  | p.Lys467* | NGS |  | Nonsense |  | 4215 | 23 |  | 0.005 | Low (<2%) |
| c.8991T>G |  |  | NGS |  | Missense |  | 4215 | 6 |  | 0.001 | Low (<2%) |
| c.8910G>A | 3 | p.Trp2970Ter | NGS | rs886040799 | stop_gained | Pathogenic | 137 | 1 |  | 0.007 | Low (<2%) |
| c.8969G>A | 3 | p.Trp2990Ter | NGS | rs80359148 | stop_gained | Pathogenic | 137 | 1 |  | 0.007 | Low (<2%) |
| c.5800C>T | 11 | p.Gln1934Ter | NGS | rs886040610 | stop_gained | Pathogenic | 137 | 1 |  | 0.007 | Low (<2%) |
| c.6125A>G | 11 | p.Gln2042Arg | NGS | rs80358852 | Missense | Conficting of pathogenicity | 137 | 1 |  | 0.007 | Low (<2%) |
| c.9976A>T | 27 | p.Lys3326Ter |  | rs11571833 | SNP |  | 79 | 1 | Shah ND et al. (31) | 0.013 | Low (<2%) |
| c.2971A>G | 11 | p.Asn991Asp |  | rs1799944 | SNP |  | 79 | 7 |  | 0.089 | Moderate (5–29%) |
| c.4779A>C | 11 | p.Glu1593Asp |  | rs80358703 | SNP |  | 79 | 2 |  | 0.025 | Low-Moderate (2–4%) |
| c.865A>C | 10 | p.Asn289His |  | rs766173 | SNP |  | 79 | 9 |  | 0.114 | Moderate (5–29%) |
| c.5744C>T | 11 | p.Thr1915Met |  | - | SNP |  | 79 | 3 |  | 0.038 | Low-Moderate (2–4%) |
| c.7397T>C | 14 | p.Val2466Ala |  | - | SNP |  | 79 | 6 |  | 0.076 | Moderate (5–29%) |
| c.1114A>C | 11 | p.Asn372His |  | rs144848 | SNP |  | 79 | 4 |  | 0.051 | Moderate (5–29%) |
| c.8117A>G | 18 | p.Asn2706Ser |  | rs80359055 | SNP |  | 79 | 1 |  | 0.013 | Low (<2%) |
| c.9380G>A | 25 | p.Trp3127Ter |  | rs80359211 | Nonsense |  | 79 | 1 |  | 0.013 | Low (<2%) |
| c.125A>G | 3 | p.Try42Cys |  | rs4987046 | SNP |  | 79 | 1 |  | 0.013 | Low (<2%) |
| c.4258G>T | 11 | p.Asp1420Tyr |  | rs28897727 | SNP |  | 79 | 1 |  | 0.013 | Low (<2%) |
| c.943T>A | 10 | p.Cys315Ser |  | rs79483201 | SNP |  | 79 | 1 |  | 0.013 | Low (<2%) |
| c.6025C>T |  | W194X | Targeted Sequencing | rs80358810 | nonsense | pathogenic | 467 | 1 | Yang XR et al. (26) | 0.002 | Low (<2%) |
| c.3109C>T |  | Q1037X | Targeted Sequencing | rs80358557 | nonsense | pathogenic | 467 | 1 |  | 0.002 | Low (<2%) |

**Supplementary Table S5.10. List of BRCA2 filtered SNPs in the Chinese Population**

| **Variant** | **Exon** | **Protein Change** | **Detection Method** | **Db_Snp150** | **Mutation Type** | **Variant**  **Impact** | **Cases Tested** | **Carriers** | **Reference#** | **Prevalence** | **Prevalence category** |
| --- | --- | --- | --- | --- | --- | --- | --- | --- | --- | --- | --- |
| c.3883C>T | - | p.Q1295X | NGS | - | N | Pathogenic | 71 | 1 | Fang M et al.(28) | 0.014 | Low (<2%) |
| c.3109C>T | 11 | p.Gln1037Ter | NGS + Sanger | - | N | Pathogenic | 595 | 1 | Liang Y et al. (29) | 0.002 | Low (<2%) |
| c.5959C>T | 11 | p.Gln1987Ter | NGS + Sanger | - | N | Pathogenic | 595 | 1 |  | 0.002 | Low (<2%) |
| c.7480C>T | 15 | p.Arg2494Ter | NGS + Sanger | - | N | Pathogenic | 595 | 1 |  | 0.002 | Low (<2%) |
| c.3559G>T | 11 | p.Glu1187Ter | NGS + Sanger | - | N | Pathogenic | 595 | 1 |  | 0.002 | Low (<2%) |
| c.8827C>T | 22 | p.Gln2943Ter | NGS + Sanger | - | N | Pathogenic | 595 | 1 |  | 0.002 | Low (<2%) |
| c.8517C>A | 20 | p.Tyr2839Ter | NGS + Sanger | - | N | Pathogenic | 595 | 1 |  | 0.002 | Low (<2%) |
| c.9317G>A | 25 | p.Trp3106Ter | NGS + Sanger | - | N | Pathogenic | 595 | 1 |  | 0.002 | Low (<2%) |
| c.8951C>G | 22 | p.Ser2984Ter | NGS + Sanger | - | N | Pathogenic | 595 | 1 |  | 0.002 | Low (<2%) |
| c.6952C>T | 13 | p.Arg2318Ter | NGS + Sanger | - | N | Pathogenic | 595 | 1 |  | 0.002 | Low (<2%) |
| c.2971A>G | 11 | A>G | NGS | - | M | Good prognosis | 82 | 7 | Shen M et al.(36) | 0.085 | Moderate (5–29%) |
| c.10234A>G | 27 | p.Ile3412Val | PGM & Miseq | - | M |  | 54 | 4 | Shen M et al.(36) | 0.074 | Moderate (5–29%) |
| c.8187G>T | 18 | p.Lys2729Asn | PGM & Miseq | - | M |  | 54 | 1 |  | 0.019 | Low (<2%) |
| c.5852G>A | 11 | p.Ser1951Asn | PGM & Miseq | - | M | Uncertain | 54 | 1 |  | 0.019 | Low (<2%) |
| c.5785A>G | 11 | p.Ile1929Val | PGM & Miseq | - | M |  | 54 | 2 |  | 0.037 | Low-Moderate (2–4%) |
| c.2971A>G | 11 | p.Asn991Asp | PGM & Miseq | - | M |  | 54 | 15 |  | 0.278 | Moderate (5–29%) |
| c.1462A>G | 10 | p.Ile488Val | PGM & Miseq | - | M | Uncertain | 54 | 1 |  | 0.019 | Low (<2%) |
| c.1399A>T | 10 | p.Lys467Te | PGM & Miseq | - | Stop_gained | Pathogenic | 54 | 1 |  | 0.019 | Low (<2%) |
| c.1114A>C | 10 | p.Asn372His | PGM & Miseq | - | M |  | 54 | 31 |  | 0.574 | Extremely High (≥50%) |
| c.865A>C | 10 | p.Asn289His | PGM & Miseq | - | M |  | 54 | 15 |  | 0.278 | Moderate (5–29%) |
| c.461A>G | 5 | p.Gln154Arg | PGM & Miseq | - | M | Uncertain | 54 | 1 |  | 0.019 | Low (<2%) |
| c.10150C>G | 27 | p.Arg3384Gly | PGM & Miseq | - | M |  | 54 | 1 |  | 0.019 | Low (<2%) |
| c.9294C>G | 25 | p.Tyr3098Ter | PGM & Miseq | - | Stop_gained | Pathogenic | 54 | 1 |  | 0.019 | Low (<2%) |
| c.3445A>G | 11 | p.Met1149Val | PGM & Miseq | - | M | Pathogenic | 54 | 1 |  | 0.019 | Low (<2%) |
| c.439C>T |  | p.Arg147Trp | NGS | - | S | Pathogenic | 2216 | 1 | Yu S et al. (56) | 0.000 | Low (<2%) |
| c.2471T>G |  | p.Leu824Arg | NGS | - | S | Pathogenic | 2216 | 1 |  | 0.000 | Low (<2%) |
| c.3109C>T |  | p.Arg1037Cys | NGS | - | S | Pathogenic | 2216 | 3 |  | 0.001 | Low (<2%) |
| c.5682C>G |  | p.Tyr1894* (Stop codon) | NGS | - | S | Pathogenic | 2216 | 5 |  | 0.002 | Low (<2%) |
| c.7090G>T |  | p.Gly2364Cys | NGS | - | S | Pathogenic | 2216 | 1 |  | 0.000 | Low (<2%) |
| c.8377G>T |  | p.Glu2793* (Stop codon) | NGS | - | S | Pathogenic | 2216 | 2 |  | 0.001 | Low (<2%) |
| c.10150C>T |  | p.Arg3384Cys | NGS | - | S | Pathogenic | 2216 | 1 |  | 0.000 | Low (<2%) |

**Supplementary Table S5.11. List of BRCA2 filtered SNPs in the Black or African Descent**

| **Variant** | **Exon** | **Protein Change** | **Detection Method** | **Db_Snp150** | **Mutation Type** | **Variant**  **Impact** | **Cases Tested** | **Carriers** | **Reference#** | **Prevalence** | **Prevalence category** |
| --- | --- | --- | --- | --- | --- | --- | --- | --- | --- | --- | --- |
| c.1103C>G | 10 | p.Pro368Arg | MLPA |  | N |  | 369 | 1 | Pal T et al. (20) | 0.003 | Low (<2%) |
| c.6137C>A | 11 | p.Ser2046* (Stop codon) | MLPA |  | N |  | 369 | 1 |  | 0.003 | Low (<2%) |
| c.8777T>A | 22 | p.Leu2926His | MLPA |  | N |  | 369 | 1 |  | 0.003 | Low (<2%) |
| c.8969G>A | 23 | p.Arg2990His | MLPA |  | N |  | 369 | 1 |  | 0.003 | Low (<2%) |
| c.9382C>T | 25 | p.Arg3128Cys | MLPA |  | N |  | 369 | 1 |  | 0.003 | Low (<2%) |
| c.7878G>A | 17 | p.Trp2626Ter | NGS | rs80359013 |  | Pathogenic | 6 | 5 | Rioki JN et al. (54) | 0.833 | Extremely High (≥50%) |
| c.9154C>T | 24 | p.Arg3052Trp | NGS | rs45580035 |  | Pathogenic | 6 | 3 |  | 0.500 | Extremely High (≥50%) |
| c.8243G>A | 18 | p.Gly2748Asp | NGS | rs80359071 |  | Pathogenic | 6 | 5 |  | 0.833 | Extremely High (≥50%) |
| c.7976G>A | 17 | p.Arg2659Lys | NGS | rs80359027 |  | Pathogenic | 6 | 5 |  | 0.833 | Extremely High (≥50%) |
| c.8165C>G | 18 | p.Thr2722Arg | NGS | rs80359062 |  | Pathogenic | 6 | 5 |  | 0.833 | Extremely High (≥50%) |
| c.8167G>C | 18 | p.Asp2723His | NGS | rs41293511 |  | Pathogenic | 6 | 5 |  | 0.833 | Extremely High (≥50%) |
| c.8168A>T | 18 | p.Asp2723Val | NGS | rs41293513 |  | Pathogenic/likely pathogenic | 6 | 5 |  | 0.833 | Extremely High (≥50%) |
| c.7988A>G | 18 | p.Glu2663Gly | NGS | rs80359031 |  | Uncertain significance | 6 | 5 |  | 0.833 | Extremely High (≥50%) |
| c.7879A>G | 17 | p.Ile2627Val | NGS | rs80359014 |  | Uncertain significance | 6 | 5 |  | 0.833 | Extremely High (≥50%) |
| c.1773G>A | - | Met591Ile | NGS | rs80359304 | Frameshift | Pathogenic | 31 | 1 | Ricks-Santi L et al. (24) | 0.032 | Low-Moderate (2–4%) |
| c.1786G>C | - | Asp596His | NGS | rs56328701 | Missense | VUS | 31 | 1 |  | 0.032 | Low-Moderate (2–4%) |
| c.8917C>T | - | Arg2973Cys | NGS | rs45469092 | Missense | VUS | 31 | 1 |  | 0.032 | Low-Moderate (2–4%) |
| c.6513G>C | - | Val2171Val | NGS | rs206076 | Synonymous | VUS | 31 | 31 |  | 1.000 | Extremely High (≥50%) |

**Supplementary Table S3.12. List of BRCA2 filtered SNPs in the European Population**

| **Variant** | **Exon** | **Protein Change** | **Detection Method** | **Db_Snp150** | **Mutation Type** | **Variant**  **Impact** | **Cases Tested** | **Carriers** | **Reference#** | **Prevalence** | **Prevalence category** |
| --- | --- | --- | --- | --- | --- | --- | --- | --- | --- | --- | --- |
| c.8191C>T |  | p.Gln2731Ter | NGS |  | N | Pathogenic | 75 | 1 | Szczerba E et al. (48) | 0.013 | Low (<2%) |
| c.5645C>A |  | p.Ser1882Ter | NGS |  | N | Pathogenic | 75 | 1 |  | 0.013 | Low (<2%) |
| c.7758G>A |  | p.Trp2586Ter | NGS |  | N | Pathogenic | 75 | 1 |  | 0.013 | Low (<2%) |
| c.631G>A |  | p.(Val211Ile) | NGS |  | SNV | Pathogenic | 376 | 1 | Stella S et al. (50) | 0.003 | Low (<2%) |
| c.9004G>A |  | p.(Glu3002Lys) | NGS |  | SNV | Pathogenic | 376 | 1 |  | 0.003 | Low (<2%) |

**Supplementary Table S5.13. List of BRCA2 filtered SNPs in the Hispanic/Latino Population**

| **Variant** | **Exon** | **Protein Change** | **Detection Method** | **Db_Snp150** | **Mutation Type** | **Variant**  **Impact** | **Cases Tested** | **Carriers** | **Reference#** | **Prevalence** | **Prevalence category** |
| --- | --- | --- | --- | --- | --- | --- | --- | --- | --- | --- | --- |
| c.2147A>G |  | p.Gln716Arg | NGS |  | M | Non-synonymous | 231 | 1 | Cortés C et al. (38) | 0.004 | Low (<2%) |
| c.2229T>C |  | p.His743His | NGS |  | Synonymous | Synonymous | 231 | 2 |  | 0.009 | Low (<2%) |
| c.2313A>T |  | p.Leu771Phe | NGS |  | M | Non-synonymous | 231 | 1 |  | 0.004 | Low (<2%) |
| c.2386G>A |  | p.Asp796Asn | NGS |  | M | Non-synonymous | 231 | 18 |  | 0.078 | Moderate (5–29%) |
| c.2454T>A |  | p.Asn818Lys | NGS |  | M | Non-synonymous | 231 | 1 |  | 0.004 | Low (<2%) |
| c.2971A>G |  | p.Asn991Asp | NGS |  | M | Non-synonymous | 231 | 7 |  | 0.030 | Low-Moderate (2–4%) |
| c.3095A>T |  | p.Lys1032Ile | NGS |  | M | Non-synonymous | 231 | 1 |  | 0.004 | Low (<2%) |
| c.3396T>G |  | p.Lys1132Lys | NGS |  | Synonymous | Synonymous | 231 | 23 |  | 0.100 | Moderate (5–29%) |
| c.3807T>C |  | p.Val1269Val | NGS |  | Synonymous | Synonymous | 231 | 8 |  | 0.035 | Low-Moderate (2–4%) |
| c.4563A>G |  | p.Leu1521Leu | NGS |  | Synonymous | Synonymous | 231 | 80 |  | 0.346 | High (30–49%) |
| c.6513C>G |  | p.Val2171Val | NGS |  | Synonymous | Synonymous | 231 | 75 |  | 0.325 | High (30–49%) |
| c.145G>T |  | p.Glu49Ter | NGS | rs80358435 | N | Pathogenic | 252 | 1 | Millan Catalan O et al.(41) | 0.004 | Low (<2%) |
| c.3166C>T |  | p.Gln1056Ter | NGS | rs79728106 | N | Pathogenic | 252 | 1 |  | 0.004 | Low (<2%) |
| c.8219T>G |  | p.Leu2740Ter | NGS | rs80359070 | N | Pathogenic | 252 | 1 |  | 0.004 | Low (<2%) |
| c.214A>C | 3 | p.Asn72His | NGS |  | M | Likely pathogenic/pathogenic | 443 | 1 | Solano AR et al. (47) | 0.002 | Low (<2%) |
| c.1337T>A | 10 | p.Leu446* | NGS |  | N | Likely pathogenic/pathogenic | 443 | 1 |  | 0.002 | Low (<2%) |
| c.2830A>T | 11 | p.Lys944* | NGS |  | N | Likely pathogenic/pathogenic | 443 | 1 |  | 0.002 | Low (<2%) |
| c.4928T>C | 11 | p.Val1643Ala | NGS |  | M | Likely pathogenic/pathogenic | 443 | 1 |  | 0.002 | Low (<2%) |
| c.5682C>G | 11 | p.Tyr1894* | NGS |  | N | Likely pathogenic/pathogenic | 443 | 1 |  | 0.002 | Low (<2%) |
| c.7480C>T | 15 | p.Arg2494* | NGS |  | N | Likely pathogenic/pathogenic | 443 | 1 |  | 0.002 | Low (<2%) |
| c.7985C>T | 18 | p.Thr2662Met | NGS |  | M | Likely pathogenic/pathogenic | 443 | 1 |  | 0.002 | Low (<2%) |
| c.8351G>A | 19 | p.Arg2784Gln | NGS |  | M | Likely pathogenic/pathogenic | 443 | 1 |  | 0.002 | Low (<2%) |
| c.8942A>G | 22 | p.Glu2981Gly | NGS |  | M | Likely pathogenic/pathogenic | 443 | 1 |  | 0.002 | Low (<2%) |
| c.9481A>T | 25 | p.Lys3161* | NGS |  | N | Likely pathogenic/pathogenic | 443 | 1 |  | 0.002 | Low (<2%) |
| c.8878C>T |  |  | NGS | rs80359140 | N | Pathogenic | 12 | 1 |  | 0.083 | Moderate (5–29%) |
| c.8878C>T |  |  | NGS | rs80359140 | N | Pathogenic | 12 | 1 |  | 0.083 | Moderate (5–29%) |
| c.936G>A | - | W312X | Sequencing | - | N | Pathogenic | 853 | 1 | Briceño-Balcázar I et al. (27) | 0.001 | Low (<2%) |
| c.2254C>T | 11 | Q742X | Sanger / NGS | - | N | Pathogenic | 190 | 1 | Villarreal-Garza C et al. (19) | 0.005 | Low (<2%) |
| c.936G>A | - | W312X | Sequencing | - | N | Pathogenic | 853 | 1 | Briceño-Balcázar I et al. (27) | 0.001 | Low (<2%) |

**Supplementary Table S5.14. List of BRCA2 filtered SNPs in the Middle Eastern/North African Population**

| **Variant** | **Exon** | **Protein Change** | **Detection Method** | **Db_Snp150** | **Mutation Type** | **Variant**  **Impact** | **Cases Tested** | **Carriers** | **Reference#** | **Prevalence** | **Prevalence category** |
| --- | --- | --- | --- | --- | --- | --- | --- | --- | --- | --- | --- |
| c.6025C>T | 11 | p.Gln2009X | Capture/Sanger Sequencing | - | N | Pathogenic | 818 | 3 | Bu R et al. (22) | 0.004 | Low (<2%) |
| c.7007G>A | 13 | p.Arg2336His | Capture/Sanger Sequencing | - | M | Pathogenic | 818 | 1 |  | 0.001 | Low (<2%) |
| c.9502_131G>A | - | - | NGS/Sanger | - | M | Pathogenic | 310 | 1 | Abulkhair O et al. (32) | 0.003 | Low (<2%) |
| c.6685G>T | 11 | p.Glu2229Ter | NGS |  | N |  | 616 | 3 | Abdel-Razeq H et al. (30) | 0.005 | Low (<2%) |
| c.2677C>T | 11 | p.Gln893Ter | NGS |  | N |  | 616 | 1 |  | 0.002 | Low (<2%) |
| c.6193C>T | 11 | p.Gln2065Ter | NGS |  | N |  | 616 | 1 |  | 0.002 | Low (<2%) |
| c.6022A>T | 11 | p.Tyr2008Ter | NGS |  | N |  | 616 | 1 |  | 0.002 | Low (<2%) |
| c.7007G>A | 13 | p.Arg2336His | NGS |  | M |  | 616 | 1 |  | 0.002 | Low (<2%) |
| c.8140C>T | 16 | p.Gln2714Ter | NGS |  | N |  | 616 | 1 |  | 0.002 | Low (<2%) |
| c.8876C>G | 22 | p.Gln2960Ter | NGS |  | N |  | 616 | 1 |  | 0.002 | Low (<2%) |
| c.8760T>G | 23 | p.Tyr2920Ter | NGS |  | N |  | 616 | 1 |  | 0.002 | Low (<2%) |
| c.100G>T | 3 | c.100G>T | NGS | rs80358391 | N | Pathogenic | 70 | 1 | Hassan AN et al. (57) | 0.014 | Low (<2%) |
| c.3318C>G | 11 | c.3318C>G | NGS | rs1298550035 | M | Conflicting interpretations of pathogenicity | 70 | 1 |  | 0.014 | Low (<2%) |
| c.6966G>T | 13 | c.6966G>T | NGS | rs80358924 | M | Uncertain significance | 70 | 1 |  | 0.014 | Low (<2%) |
| c.1819A>T | 10 | p.Lys607Ter | NGS |  | N | Pathogenic | 1336 | 1 | Al Amri WS et al. (58) | 0.001 | Low (<2%) |
| c.1574C>G | 10 | p.Thr525Ser | NGS |  | N |  | 1336 | 1 |  | 0.001 | Low (<2%) |
| c.1694C>T | 10 | p.Ala565Val | NGS |  | M |  | 1336 | 1 |  | 0.001 | Low (<2%) |
| c.800G>A | 10 | p.Gly267Glu | NGS |  | M |  | 1336 | 1 |  | 0.001 | Low (<2%) |
| c.9586A>G | 26 | p.Lys3196Glu | NGS |  | M |  | 1336 | 1 |  | 0.001 | Low (<2%) |
| c.1423G>T | 10 | p.Glu475Ter | NGS |  | N |  | 1336 | 1 |  | 0.001 | Low (<2%) |
| c.8530G>A | 20 | p.Glu2844Lys | NGS |  | N | VUS | 1336 | 1 |  | 0.001 | Low (<2%) |
| c.6344T>A | - | L2039X | NGS/Sanger | - | N | Deleterious | 100 | 1 | Rweyemamu LP et al.(52) | 0.010 | Low (<2%) |
| c.6913G>T | - | E2229X | NGS/Sanger | - | N | Deleterious | 100 | 1 |  | 0.010 | Low (<2%) |
| c.730C>G | - | P168A | NGS/Sanger | - | M | VUS | 100 | 1 |  | 0.010 | Low (<2%) |
| c.980C>G | - | T251R | NGS/Sanger | - | M | VUS | 100 | 1 |  | 0.010 | Low (<2%) |
| c.7144G>C | - | A2306P | NGS/Sanger | - | M | VUS | 100 | 1 |  | 0.010 | Low (<2%) |
| c.9002A>G | - | Q2925R | NGS/Sanger | - | M | VUS | 100 | 2 |  | 0.020 | Low-Moderate (2–4%) |
| c.6805G>A | - | E2193K | NGS/Sanger | - | M | VUS | 100 | 1 |  | 0.010 | Low (<2%) |
| c.290A>G | - | K21R | NGS/Sanger | - | M | FP | 100 | 0 |  | 0.000 | Low (<2%) |
| c.10474A>G | - | K3416E | NGS/Sanger | - | M | FP | 100 | 2 |  | 0.020 | Low-Moderate (2–4%) |

**Supplementary Table S5.15. List of BRCA2 filtered SNPs in the Unclassified Population**

| **Variant** | **Exon** | **Protein Change** | **Detection Method** | **Db_Snp150** | **Mutation Type** | **Variant**  **Impact** | **Cases Tested** | **Carriers** | **Reference#** | **Prevalence** | **Prevalence category** |
| --- | --- | --- | --- | --- | --- | --- | --- | --- | --- | --- | --- |
| c.3318C>G |  | p.S1106R | NGS |  | M |  | 99 | 1 | Geredeli C et al. (37) | 0.010 | Low (<2%) |
| c.9317G>A |  | P.W3106X | NGS |  | M |  | 99 | 1 |  | 0.010 | Low (<2%) |
